# How multiple air pollutants affect hand, foot, and mouth disease incidence in children: assessing effect modification by geographical context in multicity of Sichuan, southwest China

**DOI:** 10.1186/s12889-023-17484-9

**Published:** 2024-01-23

**Authors:** Caiying Luo, Yue Ma, Kai Lu, Ying Li, Yaqiong Liu, Tao Zhang, Fei Yin, Tiejun Shui

**Affiliations:** 1 West China School of Public Health and West China Fourth Hospital, Sichuan University, Chengdu, China; 2 Yunnan Center for Disease Control and Prevention, Kunming, China; 3 Sichuan Center for Disease Control and Prevention, Chengdu, China

**Keywords:** Hand, Foot and mouth disease, Air pollution, Attributable risk, Modification effect, Multicity analysis

## Abstract

**Background:**

Several studies have suggested a significant association of hand, foot, and mouth disease (HFMD) with ambient air pollutants. Existing studies have characterized the role of air pollutants on HFMD using only risk ratio measures while ignoring the attributable burden. And whether the geographical context (i.e., diverse topographic features) could modulate the relationships is unclear.

**Methods:**

Daily reported childhood HFMD counts, ambient air pollution, and meteorological data during 2015–2017 were collected for each of 21 cities in Sichuan Province. A multistage analysis was carried out in different populations based on geographical context to assess effect modification by topographic conditions. We first constructed a distributed lag nonlinear model (DLNM) for each city to describe the relationships with risk ratio measures. Then, we applied a multivariate meta-regression to estimate the pooled effects of multiple air pollutants on HFMD from the exposure and lagged dimensions. Finally, attributable risks measures were calculated to quantify HFMD burden by air pollution.

**Results:**

Based on 207554 HFMD cases in Sichuan Province, significant associations of HFMD with ambient air pollutants were observed mainly at relatively high exposure ranges. The effects of ambient air pollutants on HFMD are most pronounced on lag0 or around lag7, with relative risks gradually approaching the reference line thereafter. The attributable risks of O_3_ were much greater than those of other air pollutants, particularly in basin and mountain regions.

**Conclusions:**

This study revealed significant pooled relationships between multiple air pollutants and HFMD incidence from both exposure and lag dimensions. However, the specific effects, including RRs and ARs, differ depending on the air pollution variable and geographical context. These findings provide local authorities with more evidence to determine key air pollutants and regions for devising and implementing targeted interventions.

**Supplementary Information:**

The online version contains supplementary material available at 10.1186/s12889-023-17484-9.

## Background

Hand, foot, and mouth disease, an acute intestinal infectious disease, is prevalent among children below 5 years old worldwide [1]. During recent decades, multiple HFMD epidemics have occurred mostly in the Southeast regions, with considerable numbers of infections. Most HFMD cases are self-limiting and characterized by common symptoms such as fever and rash, while a minority of infected children, especially those under the age of 3, may experience severe complications of the central nervous system and circulatory system, which can even lead to death [2]. A variety of human enteroviruses can cause HFMD through respiratory droplets, the fecal–oral route, and close contact. Human enterovirus A71 (EV-71) and coxsackievirus A16 (CVA16) are the primary pathogenic serotypes that lead to HFMD outbreaks [3]. However, in recent years, the dominant causative agent of HFMD has undergone a shift with the rapid surge in cases caused by CV-A6 and CV-A10 [4, 5]. Regrettably, effective antiviral drugs for the prevention and treatment of HFMD are still unavailable. In 2016, several monovalent EV-A71 vaccines were launched in China. These vaccines exhibited high effectiveness in preventing HFMD associated with EV-A71 and mitigating the severity of HFMD in infected patients [6, 7]. Nonetheless, individuals susceptible to HFMD must pay the full price of the vaccines to acquire protection exclusively against EV-A71 without efficient cross-protection against other enterovirus serotypes [6, 8]. Even after the approval of EV-A71 vaccines, the HFMD remains a high prevalent disease which poses an important public health threat to child health [9–11]. HFMD persists with a much higher incidence rate than other notifiable infectious diseases in mainland China, with more than one million infected cases each year [9–13].

HFMD exhibits distinct seasonal patterns [14]. Previous epidemiological studies have demonstrated the relationship between environmental variables, such as temperature, relative humidity, and other meteorological factors, and HFMD incidence [15, 16]. In recent years, with the rapid development of economic growth, urbanization, and industrialization, the effects of ambient air pollutants on health have gained considerable attention from researchers [17, 18]. Exploring the impacts of air pollutants on HFMD can help clarify the potential factors, which further provide environmental evidence for targeted policy recommendations and prevention strategies. Nonetheless, several studies have quantified the associations of HFMD with multiple air pollutants, which showed regional inconsistency. For example, a study in Hefei revealed a linear relationship between SO_2_ and HFMD, with the highest cumulative effect at lag day 0–1 among scattered children [19]. In contrast, Gu et al.proposed a heterogeneous J-shape relationship between HFMD and SO_2_ in Ningbo [20]. In addition, O_3_ has been found to have an inverse V-shape relationship with HFMD in Ningbo [20], while an M-shape pattern was observed in Shenzhen [21]. In addition, studies were usually carried out in a single city with moderate or mild air pollution and applying methods with different relationship assumptions or model specifications. Hence, the generalizability and comparability of the findings were limited across multiple locations. Furthermore, previous studies relied solely on risk ratio measures such as RR and OR to evaluate the impact of ambient air pollution variables on HFMD, which provided only partial information about the excess burden of HFMD caused by air pollution. To date, there is no evidence on the excess burden of HFMD attributable to air pollution. Therefore, it is necessary to rely on multiple insights viewing the role of air pollutants in HFMD through appropriate multicity strategies and attributable indices (i.e., AF and AN) to help elucidate the possible influencing factors and supplement the evidence for health effect estimation.

Epidemiological studies have shown that a few city-specific characteristics, such as socioeconomic status and climatic conditions, could modify the associations of HFMD with ambient air pollution [22, 23]. The topographic condition of a city is a critical characteristic that can reflect its unique natural environment and have direct and indirect impacts on the social and economic conditions of the city. However, whether topography acts as a comprehensive factor affecting the association of ambient air pollutants with HFMD has not yet been investigated. Sichuan Province, located in Southwest China, encompasses a variety of topographic conditions, including basins, plateaus, and mountains. Meanwhile, Sichuan Province has imbalanced socioeconomic development and diverse climate conditions across different topographies [24]. Consequently, the distributions of air pollution concentrations vary greatly across its topographies in Sichuan Province [25]. Sichuan Basin, in particular, has emerged as a most heavily air polluted region in China [26]. Therefore, Sichuan Province could serve as a representative region to investigate and compare the complex associations between air pollution and HFMD across different geographical areas under the combined impacts of the environment and social economy, particularly to provide novel quantitative evidence in heavily polluted areas.

This study aimed to explore and compare the relationships in the context of heavy air pollution utilizing DLNM in three geographical regions (i.e., Sichuan Basin, West Sichuan Plateau, and Southwest Sichuan Mountain): a) the pooled cumulative effects of air pollution on HFMD; b) the pooled lag effects of air pollution on HFMD; c) the attributable risk of air pollution on HFMD.

## Material and methods

### Setting and data

**Fig. 1:**
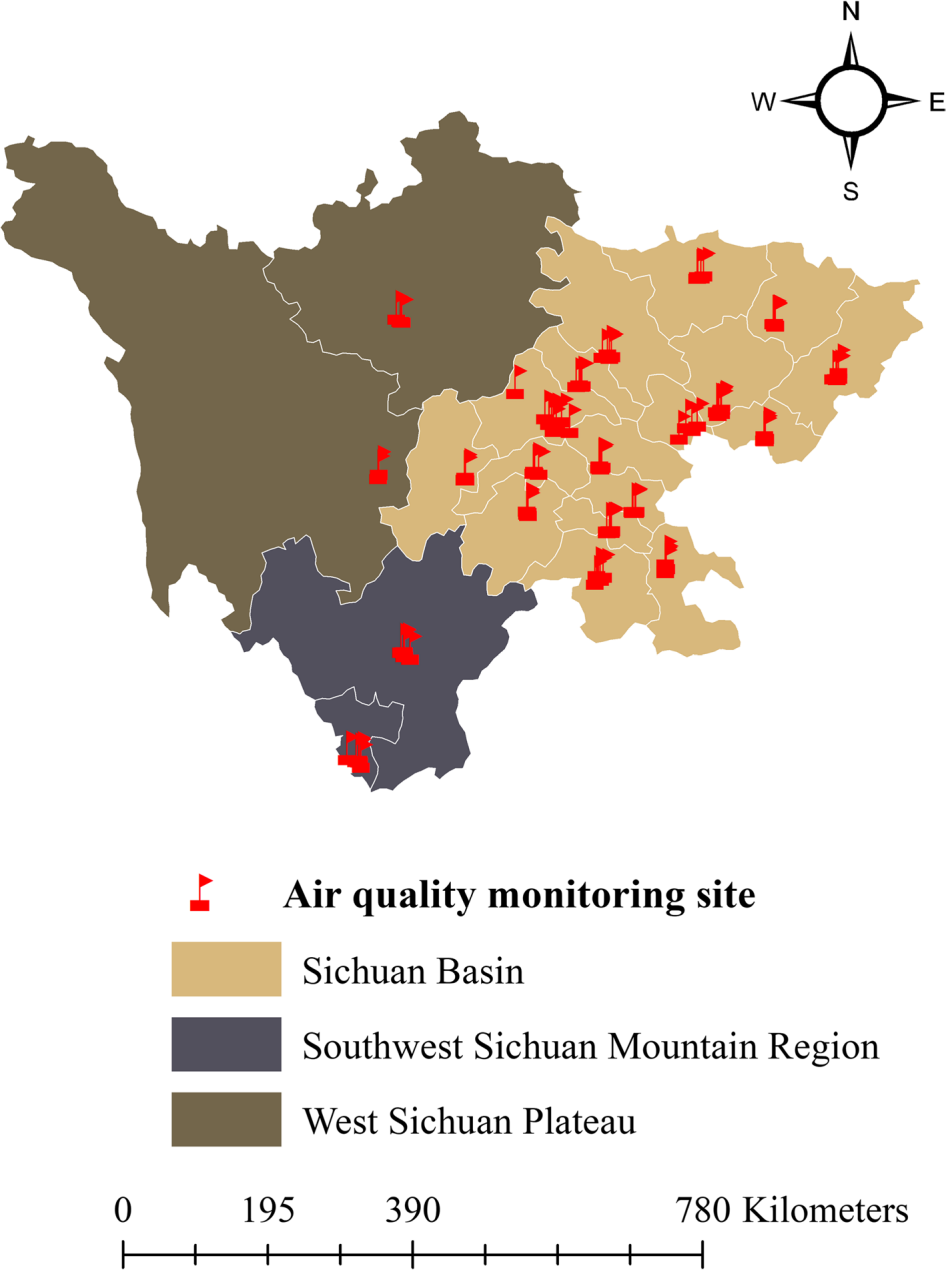
Geographic locations for the 21 cities and air quality monitoring stations in Sichuan Province

Sichuan Province, composed of 21 cities, covers a total area of more than 486,000 square kilometers in Southwest China. Sichuan Province has a diverse and complex topography with five typical landforms, including basins, plains, hills, mountains, and plateaus. Additionally, Sichuan Province exhibits persistent regional variations in climate. The eastern Sichuan Basin is located in the subtropical humid climate zone. The southwestern Mountain and western Plateau are located in the subtropical and high-altitude cold-climate zones, respectively [27]. Considering the unique climatic and geographical characteristics, the national guide of divisions published by the China Meteorological Administration [28] divides Sichuan Province into 3 regions: the Sichuan Basin (Chengdu, Zigong, Deyang, and 14 other cities), West Sichuan Plateau (Aba and Ganzi), and Southwest Sichuan Mountain (Liangshan and Panzhihua) (Fig. 1).

On May 2, 2008, HFMD was cataloged into a notifiable infectious disease classified C under the unified report and standard management. The surveillance data for daily HFMD cases during 2015–2017 were retrieved from the national information system for disease control and prevention managed by the China CDC. Each reported patient can be extracted following information, including demographic value (gender, date of birth, and address), case classification, and date of onset. We compiled all of the case information into daily time series data of HFMD counts for each of the 21 cities in Sichuan Province. As previous studies demonstrated that children below 5 years old were the most vulnerable group to infect HFMD, we only included patients aged 0–5, which accounted for an overwhelming majority of HFMD cases (> 96%) according to the preliminary analysis.

Daily air pollution data, including PM_10_ (μg/m^3^), PM_2.5_ (μg/m^3^), SO_2_ (μg/m^3^), NO_2_ (μg/m^3^), O_3_ (μg/m^3^), CO (mg/m^3^), and air quality index (AQI), for the same period in 21 cities were downloaded from the Sichuan Environmental Monitoring Center. The monitoring data are of high quality with a proportion of missing data less than 1%. Linear interpolation was used to fill in missing values.

Daily meteorological data from 41 monitoring stations in Sichuan Province were provided by the official website of National Meteorological Data Sharing Center. Seven meteorological indicators, namely, mean temperature (°C), minimum and maximum temperature (°C), relative humidity (%), air pressure (hPa), sunshine duration (h), and wind velocity (m/s), were considered in this study. The proportion of valid data for each station is greater than 99%. To fill the missing values, we used different methods according to the nature of each meteorological indicator. Missing values of sunshine duration were replaced with zero. For the other six variables, we used linear interpolation to replace missing values. To estimate daily meteorological exposures at the city level in Sichuan Province, we implemented the kriging interpolation method, allowing us to make full use of the information from all meteorological stations.

Ultimately, daily series of HFMD counts, air pollution concentrations, and meteorological data of each of the 21 cities in Sichuan Province were matched through unified city-specific codes for further analysis.

### Statistical analysis

In this study, a multistage and multicity time series regression was conducted to describe the effects of ambient air pollution variables on HFMD from multiple perspectives. In the first stage, the estimated effects of air pollution from two dimensions (i.e. exposure–response and lag-response) were quantified for each of the 21 cities in Sichuan Province. In the second stage, pooled exposure–response and lag-response relationships at the regional level were described by a multivariate meta-analysis based on the city-specific estimates from the first stage [29, 30]. In the last stage, the attributable risks of HFMD associated with air pollution were calculated based on the backward perspective [31].

#### DLNM construction

Due to the frequent occurrence of sporadic HFMD cases or outbreaks, the count data were overdispersion. Therefore, we employed the distributed lag nonlinear model assumed a quasi-Poisson distribution to quantify the impacts of multiple air pollutants on HFMD. According to our initial analysis, we found a strong correlation between PM_10_, PM_2.5_, and AQI, which could affect the accuracy of the results. To improve the comparability with previous studies and consider the possible hypotheses on biological mechanisms, only PM_10_ was retained in this study for further analysis among these highly correlated variables. We established uniform model structures and parameters for each city by considering prior knowledge and conducting a series of sensitivity analyses based on the multi-pollutant model (see more details in Additional file). The final model for each city is expressed as follows:

$$Y_{it}\sim Quasi-Poisson\left(\mu_{it}\right)$$

$$\log\left(\mu_{it}\right)\;=\alpha\;+\sum\;cb\left(\mathrm{air}\;{\mathrm{pollutants}}_{it}\right)\;+\;ns{(SMA\left(Temp\right),3)}\mathit\;+\;\sum\;\delta_{oc}SMA\left(Other\;confounders\right)\;+\;ns\left(day,\;8^\ast3\right)\;+\;\eta auto_{it}\;+\;\beta dow_t\;+\;\gamma holiday_t\;+\;offset\;\left(\log\left(Childpopu_{im}\right)\right)$$

where α is the intercept; *i* is the city code (*i* = 1,2,3,…,21); *Y*_*it*_ represents the number of daily HFMD counts in city *i* corresponding to day *t* (*t* = 1,2,3,…,1096); *Quasi-Poisson* refers to the quasi-Poisson model constructed; and *cb*(air pollutants_*it*_) is the cross basis function of PM_10_, SO_2_, NO_2_, CO, and O_3_ associated with HFMD. Specifically, the two dimensions (i.e. exposure–response and lag-response) were described by the natural cubic spline (ns) with the degrees of freedom (df) setting to 3, and the lag period was set to 0–14 days to capture the lag effects of air pollution. We defined a natural cubic spline using 8 dfs per year expressed as *ns*(*day*,8*3) in the model to remove the confounding of seasonality and long-term trend. A nonlinear term of temperature was incorporated by first summarizing multiple lags (0–14) using a simple moving average (*SMA*) and then applying a ns with 3 dfs to account for the confounding of temperature. Other meteorological factors (relative humidity, wind velocity, and sunshine duration) were included in the model by a simple-moving-average variable as *SMA(Other confounder)*. The respective coefficient of the other confounder was denoted as *δ*_oc_. We used the logarithm scale of HFMD counts at lag1 and lag2 as the autoregressive terms to adjust the residual autocorrelation. The day of week (*dow*_*t*_) and holidays (*holiday*_*t*_) were included as dummy variables. And *η*, *β*, and *γ* are coefficients of corresponding terms. *Childpop*_*im*_ was an offset term for the child population of the city *i* on year *m*. The median values of PM_10_, SO_2_, NO_2_, CO, and O_3_ across all the cities (i.e., 60.0 μg/m^3^, 13.0 μg/m^3^, 27.0 μg/m^3^, 0.8 mg/m^3^, 57.0 μg/m^3^) were defined as the reference to calculate relative risks.

#### Multivariate meta-regression

The exposure-lag-response associations of HFMD with air pollution variables were estimated in the first stage and then reduced to unidimensional vectors for the following multivariate meta-analysis. For each air pollutant, the lag-response relationships at specific air pollution levels, including the 25_th_ and 75_th_ percentiles, were extracted. In addition, the delayed effects at each air pollution level were summed to represent the cumulative air pollutants-HFMD relationship. Taking the region as the unit, a intercept model of the multivariate meta-regression was used to meta-analyze the city-specific effect estimates after dimensionality reduction to obtain the region-specific pooled lag-response relationship and cumulative exposure–response relationship.

#### Attributable risks calculation

The air pollution with the lowest RR, derived from the city-specific pooled air pollutant-HFMD curve ranging from the 5_th_ to 95_th_ percentile of the air pollutant, was taken as the counterfactual condition to calculate the attributable risk [32, 33]. For each day of the series, the cumulative RR corresponding to the air pollutant concentration obtained from the first stage was used to calculate the attributable HFMD cases and fraction in each city [31]. To quantify and compare the impacts of each air pollutant at different exposure levels, the 5_th_, 10_th_, 25_th_, 50_th_, 75_th_, 90_th_, and 95_th_ percentiles were selected as the cutoff points to calculate the ARs in low exposure intervals (*P*_0_~*P*_5,_ *P*_0_~*P*_10,_ *P*_0_~*P*_25,_ *P*_0_~*P*_50_) and high exposure intervals (*P*_50_~*P*_100,_ *P*_75_~*P*_100,_* P*_90_~*P*_100,_ *P*_95_~*P*_100_) [34]. The numbers of HFMD cases attributable to high and low exposure levels of air pollution were calculated by summing the components for all of the days in the time series that met the requirements. The region-specific ANs were further calculated and compared by summing the subsets corresponding to cities located in Sichuan Basin, West Sichuan Plateau, or Southwest Sichuan Mountain. The attributable fraction was produced by dividing the AN by the corresponding total number of HFMD cases. Monte Carlo simulations were utilized to obtain the empirical confidence intervals for AFs and ANs under the assumption of multivariate normal distribution.

All statistical analyses were performed in R 3.5.1. We adopted R packages "dlnm" and "mvmeta" to fit the DLNMs and multivariate meta-regression models, respectively. Two-tailed *P* < 0.05 were defined as statistically significant for all statistical tests.

## Results

### Descriptive statistics of research data

**Fig. 2:**
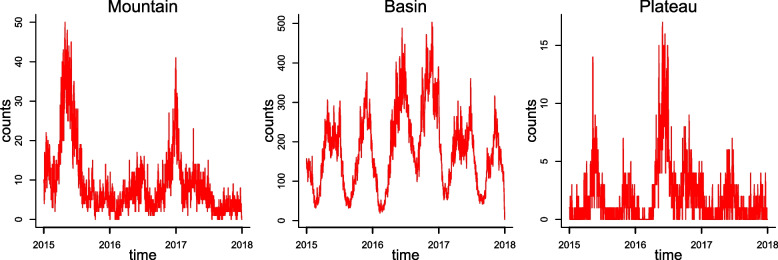
Daily distribution of HFMD cases from three regions in Sichuan Province, 2015–2017

The dataset included 207554 cases under 5 years old with HFMD in Sichuan Province during 2015–2017, with yearly totals of 62613, 86864, and 58077 cases, respectively. The HFMD cases were mostly clustered in the eastern basin, which has a high population density and relatively developed economy. We found a similar seasonal distribution of HFMD in both the Sichuan Basin and the West Sichuan Plateau. Two peaks were observed during spring (from April to June) and early winter (from November to December) (Fig. 2). In 2016, both the annual total number and the peak number of cases were higher than those in the other two years. Furthermore, we found that PM_2.5_, PM_10_, and AQI had similar distribution patterns across the different regions. High concentrations of particulate matter typically accumulated in the Sichuan Basin, while the average concentrations of SO_2_, CO, NO_2_, and O_3_ were higher in Southwest Sichuan Mountain (Table 1).

**Table 1:** Descriptive statistics of air pollution variables and HFMD cases from three regions in Sichuan Province, 2015–2017

	Mountain				Basin				Plateau			
	‾*x* ± *s*	P_25_	P_50_	P_75_	‾*x* ± *s*	P_25_	P_50_	P_75_	‾*x* ± *s*	P_25_	P_50_	P_75_
cases	4.7 ± 6.0	1	3	6	10.5 ± 20.7	2	5	10	1.0 ± 1.7	0	0	1
PM_2.5_(μg/m^3^)	28.3 ± 12.8	19	26	36	49.9 ± 35.7	25	40	65	18 ± 8.4	12	17	22
PM_10_(μg/m^3^)	52.4 ± 24.1	34	47	67	79.8 ± 49.1	45	68	103	33.6 ± 15.7	23	31	41
SO_2_(μg/m^3^)	29.6 ± 14.9	19	27	37	14.5 ± 8.5	9	13	18	13.2 ± 7.7	8	12	16
NO_2_(μg/m^3^)	27.4 ± 10.8	20	26	33	30.5 ± 12.6	21	29	38	18.7 ± 21.3	9	14	23
CO(mg/m^3^)	1.3 ± 0.5	0.9	1.2	1.5	0.9 ± 0.3	0.6	0.8	1	0.6 ± 0.2	0.4	0.5	0.7
O_3_(μg/m^3^)	67 ± 30.6	43	63	87.2	62.4 ± 35.0	37	56	81	56.7 ± 21.1	40.8	56	70
AQI	54.7 ± 16	43	53	64	78.1 ± 41.3	51	67	94	40.2 ± 11.6	33	39	46

### Pooled effects of ambient air pollution variables on HFMD in the exposure dimension

**Fig. 3:**
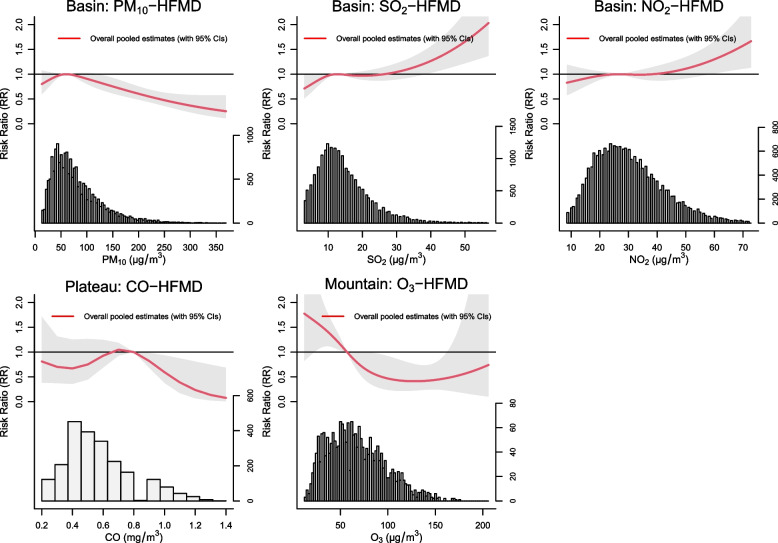
Significant overall pooled relationships pattern between multiple air pollutants and HFMD at the regional level

Our results showed significant associations of air pollutants on HFMD in three geographical divisions. In Sichuan Basin, we found cutoff values of 38.4 μg/m^3^ and 58.3 μg/m^3^ for the association of HFMD with SO_2_ and NO_2_, which correspond to the slopes of the curves nearest to zero. After that, the cumulative relative risk increased approximately linearly with SO_2_ and NO_2_. In contrast, PM_10_ displayed an approximately linear negative correlation with pediatric HFMD when the concentration exceeded the median. Similarly, in West Sichuan Plateau, once CO exceeded the median, the relative risk of HFMD began to decrease linearly. In Southwest Sichuan Mountain, we observed a significant nonlinear association between O_3_ and HFMD. The risk decreased with increasing O_3_ initially, reaching the lowest RR of 0.412 (95% *CI*: 0.224–0.759) at 127.8 μg/m^3^ of O_3_, and then the curve began to flatten (Fig. 3). In summary, significant associations were observed among all the air pollutants with HFMD in three geographical divisions, but the detailed shape patterns for the air pollutant-HFMD relationships differed across geographical divisions.

### Pooled effects of ambient air pollution variables on HFMD in the lag dimension

**Fig. 4:**
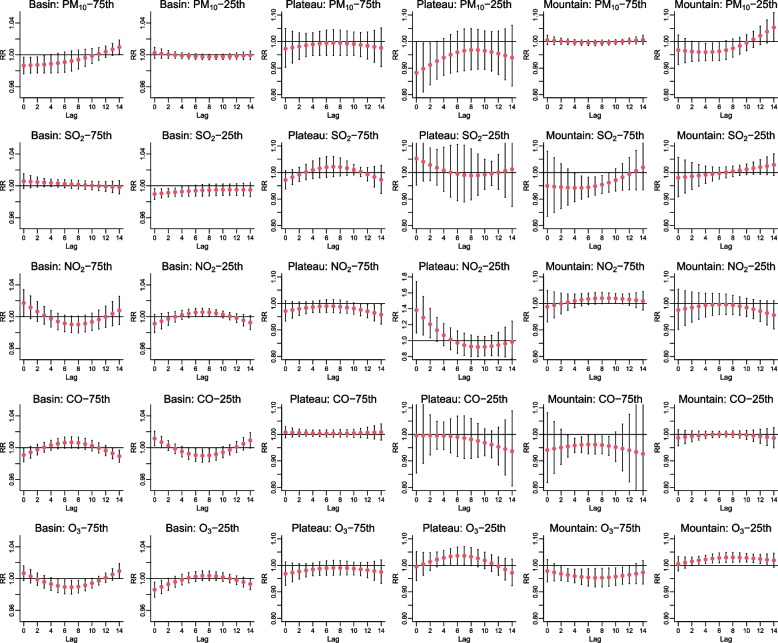
The regional pooled lagged effects of air pollutants on HFMD in three geographical regions

The results of the lag effects analysis for air pollutants at the 75_th_ percentile were different from those at the 25_th_ percentile. For example, the lag-specific curves of NO_2_ and O_3_ in the basin were V-shaped at the 75_th_ percentile, but they were opposite at the 25_th_ percentile. In Sichuan Basin, the most significant RR values were shared at lag0, with 0.986 (95%*CI*: 0.976–0.997) for the PM_10_ effect (75_th_), 0.990(95%*CI*: 0.983–0.996) for SO_2_ effect (25th), 1.011(95%*CI*: 1.002–1.020) for CO effect (25_th_), and 0.986(95%*CI*: 0.977–0.996) for O_3_ effect (25_th_). While in West Sichuan Plateau, only NO_2_ presented significant lag effects on HFMD, with the largest RR of 1.382 (95% *CI*: 1.096–1.742) at the 25_th_ percentile of NO_2_ at lag0. In Southwest Sichuan Mountain, different from other regions, prominent effects were observed at lag7, with RRs of 0.948 (95% *CI*: 0.915–0.983) for SO_2_ (75_th_), 0.953 (95% *CI*: 0.919–0.989) for O_3_ (75_th_), and 1.029 (95% *CI*: 1.010–1.049) for O_3_ (25_th_). Furthermore, significant effects of O_3_ on HFMD in Southwest Sichuan Mountain were shown from lag3 to lag12 (Fig. 4).

### Comparison of the attributable risks of air pollutants on HFMD

**Fig. 5:**
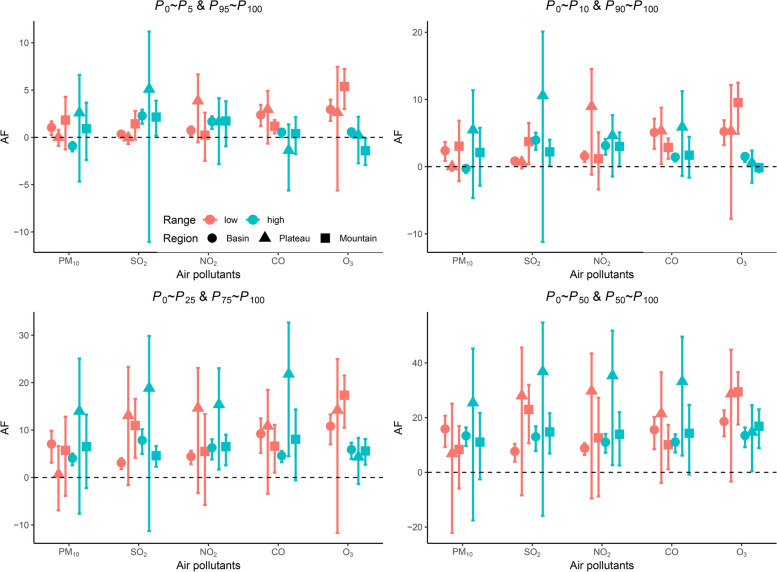
Attributable fractions of HFMD incidence by multiple air pollutants at different concentration ranges in three geographical divisions in Sichuan Province

Initially, we compared the attributable fractions for air pollutants at different concentration ranges in three geographical divisions (Fig. 5). We found that significant AFs of PM_10_, SO_2_, and NO_2_ mostly in Sichuan Basin. The health effects of O_3_ were much greater than those of other air pollutants, particularly in basin and mountain regions. Detailedly, the estimated AFs at the low-level PM_10_ concentration intervals ranged from 1.04%* (*95% *CI*: 0.24%-1.69%) to 15.88%* (*95% *CI*: 9.3%-20.72%) in the Sichuan Basin. Likewise, the AFs for the low-level CO concentration intervals were 2.38% (95% *CI*: 1.19%-3.43%), 5.10% (95% *CI*: 2.65%-7.14%), 9.19% (95% *CI*: 5.17%-12.46%), and 15.55% (95% *CI*: 8.48%-20.21%), higher than those for the corresponding heavily polluted intervals. However, in the West Sichuan Plateau, significantly high AFs were observed for the high-level CO with concentrations above the 50_th_ and the 75_th_ percentiles. The AFs for the low-level O_3_ showed the highest values as 5.38% (95% *CI*: 3%-7.24%), 9.54% (95% *CI*: 4.90%-12.49%), 17.35% (95% *CI*: 10.5%-21.48%), and 29.43% (95% *CI*: 17.46%-36.62%) in Southwest Sichuan Mountain, whereas the fractions attributed to high O_3_ intervals were much smaller (Fig. 5).

Due to the large population and a huge number of HFMD infections in Sichuan Basin, the ANs in this region for each air pollutant were also much higher than those in West Sichuan Plateau and Southwest Sichuan Mountain (Table 2 and 3). In Sichuan Basin, the ANs for high-level SO_2_ and NO_2_ account for most of the HFMD burden compared with other air pollutants. Compared with the counterfactual condition, the extreme high-level PM_10_ concentration range showed a protected effect from HFMD with an AN of 1778 (Table 2). In West Sichuan Plateau, significant ANs were only observed for high-level NO_2_ and CO concentration intervals above the 50_th_ and 75_th_ percentiles for low-level NO_2_ and CO concentration intervals below the 10_th_ percentile. In Southwest Sichuan Mountain, O_3_ was the most remarkable air pollutant with the highest ANs for low-level O_3_ concentration intervals ranging from 550 (95% *CI*: 307–741) to 3011 (95% *CI*: 1786–3747).

**Table 2:** Numbers of HFMD incidence attributable to multiple air pollutants at high concentration ranges in three geographical divisions in Sichuan Province

Air pollutants	> P_95_	> P_90_	> P_75_	> P_50_
Basin				
PM_10_	-1778(-2903, -968)	-526(-1848, 306)	7918(5045, 9881)	26128(18816, 32044)
SO_2_	4475(2826, 5724)	7682(4928, 9869)	15303(9708, 19857)	25357(15157, 32859)
NO_2_	3253(1738, 4328)	6103(3484, 8050)	12247(7431, 15723)	21529(14031, 27378)
CO	1081(37, 1751)	2799(1502, 3604)	8939(6314, 10919)	21661(14163, 27175)
O_3_	1130(109, 1805)	2973(1393, 3914)	11499(7567, 14335)	26268(17940, 32049)
Plateau				
PM_10_	55(-99, 140)	116(-99, 241)	296(-162, 531)	539(-373, 959)
SO_2_	108(-234, 237)	224(-237, 426)	399(-239, 632)	780(-336, 1160)
NO_2_	35(-60, 88)	98(-31, 162)	326(36, 488)	749(56, 1097)
CO	-29(-119, 29)	125(-29, 238)	462(95, 692)	703(130, 1050)
O_3_	5(-58, 46)	12(-51, 51)	92(-29, 177)	311(9, 520)
Mountain				
PM_10_	94(-244, 376)	217(-290, 591)	665(-228, 1357)	1133(-260, 2224)
SO_2_	220(19, 397)	225(11, 410)	469(232, 678)	1511(706, 2209)
NO_2_	177(-97, 391)	309(5, 522)	665(263, 924)	1424(259, 2253)
CO	40(-180, 218)	175(-167, 453)	825(-60, 1467)	1462(-81, 2525)
O_3_	-144(-300, -9)	-15(-83, 43)	572(276, 830)	1722(906, 2356)

**Table 3:** Numbers of HFMD incidence attributable to multiple air pollutants at low concentration ranges in three geographical divisions in Sichuan Province

Air pollutants	< P_5_	< P_10_	< P_25_	< P_50_
Basin				
PM_10_	2026(467, 3300)	4672(1665, 7165)	13810(6115, 19240)	30993(18157, 40443)
SO_2_	664(-46, 1250)	1637(565, 2442)	6048(3460, 7950)	14970(7600, 20334)
NO_2_	1436(350, 2268)	3068(1385, 4436)	8652(5455, 10982)	17233(12402, 20712)
CO	4646(2320, 6690)	9960(5175, 13937)	17936(10093, 24321)	30361(16561, 39460)
O_3_	5755(3403, 7772)	10204(6239, 13481)	21130(13622, 25991)	36314(25701, 44278)
Plateau				
PM_10_	0(-19, 17)	0(-13, 10)	14(-147, 140)	146(-468, 532)
SO_2_	-1(-15, 9)	14(-5, 28)	277(-34, 494)	593(-178, 966)
NO_2_	82(-11, 141)	190(-25, 308)	310(-69, 489)	630(-202, 920)
CO	63(-14, 104)	113(8, 186)	230(-73, 391)	456(-82, 774)
O_3_	56(-119, 158)	112(-165, 258)	301(-247, 529)	608(-71, 950)
Mountain				
PM_10_	187(-130, 439)	312(-219, 700)	585(-397, 1311)	855(-602, 1734)
SO_2_	146(-23, 287)	382(41, 666)	1120(427, 1696)	2351(1088, 3275)
NO_2_	24(-254, 267)	121(-346, 524)	562(-592, 1369)	1293(-901, 2789)
CO	121(43, 187)	294(119, 431)	675(104, 1136)	1031(112, 1773)
O_3_	550(307, 741)	976(501, 1278)	1775(1074, 2198)	3011(1786, 3747)

## Discussion

In recent years, air pollution, one of the key environmental problems affecting human health, has received wide attention from researchers. Air pollutants can increase the risk of morbidity of infectious and noninfectious diseases in the respiratory, circulatory, and nervous systems [35–37]. In this study, a multistage strategy based on DLNMs was applied to comprehensively explore the possible nonlinear and delayed associations and quantify the attributable risks between air pollutants and HFMD. The results suggested significant pooled nonlinear associations of PM_10_, CO, and O_3_ with HFMD and approximately linear relationships of SO_2_ and NO_2_ with HFMD. The pooled effects of air pollution variables on HFMD are most pronounced on lag0 or around lag7. These effects, including RRs and ARs, were closely related to geographical divisions and exposure levels. To the best of our knowledge, this study is the first to characterize the pooled lag effects of air pollution at the regional level and quantify the burden of air pollution on HFMD by calculating attributable risks.

This study suggests that further attention should be given to the effects of air pollutants in areas with high exposure concentrations. It was observed that the overall effects of PM_10_, SO_2_, and NO_2_ on HFMD were significant only in the basin area, while those of CO and O_3_ on HFMD were significant only in the plateau and mountain areas, respectively. Specifically, the risk of HFMD gets higher with increasing concentrations of SO_2_ and NO_2_, which shows consistency with previous studies. For instance, a study in Ningbo suggested that high concentrations of SO_2_ and NO_2_ elevated the risk of HFMD [20]. Shao et al. indicated that HFMD incidence is related to cytokines (e.g., TNF-α, IFN-β, IL-4, IL-12, IL-18) and chemokines, which are usually affected by SO_2_ [38]. As a result, high SO_2_ remarkably weakens the body resistance of children to viral infection. Particularly under the typical topography of the Sichuan Basin with high humidity throughout the year, due to the high solubility of SO_2_ in water, inhaled SO_2_ and its in *vivo* derivatives may rapidly enter the bloodstream and produce toxic effects in the respiratory system, decreasing the host defense responses to enteroviruses [39, 40]. Many studies have shown that NO_2_ influences the incidence of respiratory diseases in children, such as low lung function in school children [41] and childhood asthma [42, 43]. However, research on the biological mechanism and epidemiological relationship between NO_2_ and HFMD remains limited. As one of the key components of air pollution, NO_2_ is closely related to vehicle exhaust emissions. With the rapid pace of urbanization, car ownership in all cities has expanded dramatically, especially in the Sichuan Basin, which leads in both level and speed of development. The average concentration of NO_2_ in the basin is 30.5 μg/m^3^, which is much higher than that in other regions in Sichuan Province. Therefore, in the basin area, it is necessary to comprehensively consider the exposure concentrations of NO_2_ and SO_2_, along with the specific curve pattern of HFMD with air pollutants, to formulate targeted health guidance strategies.

This study found that high concentrations of PM_10_, O_3_, and CO were related to a lower risk of HFMD, with each significant air pollutant being identified in a specific geographical region. It is believed that particulate matter may facilitate the adhesion and transmission of enterovirus and that it may also influence the inflammatory response, leading to increased susceptibility to infection [44, 45]. However, due to poor visibility on polluted days with high concentrations of particulate matter, outdoor activities for children may be limited to reduce the risk of contact with enterovirus and particulate matter in the environment, ultimately leading to a reduction in the risk of HFMD associated with high particulate matter [46, 47]. Some studies conducted in Ningbo [48] and Shenzhen [21] found no significant relationship between PM_10_ and HFMD, which differs from the results of this study. This may be due to the fact that Sichuan Basin is a typical region with a high incidence of HFMD and much higher concentrations of particulate matter than the coastal cities, making the effect of PM_10_ more likely to be identified. Furthermore, evidence from another heavily polluted city, Shijiazhuang, suggests that high particulate matter may have a protective effect [49]. Therefore, we can reasonably speculate that the average concentrations of air pollutants in cities might be a potential factor that could impact the relationship pattern of HFMD with air pollution. Similarly, a significant negative association of O_3_ with HFMD was found in the mountainous area with high O_3_ concentrations. Ozone can inhibit the survival and replication of bacteria and viruses in the external environment. Some studies found that the inactivation of enterovirus EV-A71 affected by O_3_ is related to the dissolution dynamics of O_3_ [50, 51]. In contrast, the protective effect of high CO was identified in the plateau area with lower concentrations than other regions. Some studies have indicated that chronic exposure to CO may increase the risk of cardiovascular disease [52], while others have suggested that short-term exposure to low-level CO presents a protective effect and reduces the risk of hospital admission for respiratory infectious diseases [53]. The discrepancies among these results might be related to the differences in disease outcomes, geographic locations, and socioeconomic conditions. Further evidence is still needed to detailedly explore the potential reasons and better characterize the relationship between CO and HFMD.

Our findings reveal that the effects of ambient air pollution indicators on HFMD are most pronounced on lag0 or around lag7, with relative risks gradually approaching the reference line thereafter. Utilizing the lag distribution of the effects could prove advantageous in directing the prevention and control of HFMD. It is noteworthy that the effect of O_3_ was observed on the third lag day and lasted for over a week. However, single-day lag effects provided limited information. To quantify and compare the effects of air pollutants, we further calculated attributable fractions and attributable numbers under various exposure ranges and observed that the attributable risks of PM_10_, SO_2_, and NO_2_ tended to be higher in the Sichuan Basin. This may be related to the differences in the distribution of air pollutants. A comparison of the attributable fractions of air pollutants revealed that the health effects of O_3_ were much greater than those of other air pollutants, particularly in basin and mountain regions. In previous studies, temperature and relative humidity, the two most popular environmental factors, were commonly identified to have significant impacts on HFMD risks [33, 54, 55]. The attributable fractions of high temperature and relative humidity in previous studies reached 39.55% [54] and 14.56% [33], respectively. The latter was relatively close to the AFs for the high levels of PM_10_ (13.38%) and O_3_ (16.83%) that were calculated in this study. More attention should be given to the associations between HFMD and air pollutants, especially PM_10_ and O_3_, next only to temperature. In summary, in addition to the bi-dimensional pooled exposure-lag-response relationships, the measurement of attributable risk can provide new insights of association from a quantitative perspective, particularly with advantages in the comparison of HFMD burden attributed to different air pollutants.

Several limitations should be noted in this study. First, the intrinsic nature of this ecological study precludes the establishment of a causal relationship between ambient air pollution and HFMD, despite the identification of short-term associations and attributable risks. Nonetheless, the results may serve as the basis for future research hypotheses. Second, to leverage the air pollution variables to their fullest extent, data were collected only after 2015. Despite this, this study still possessed sufficient test power given the daily data that were utilized. Third, some measurement errors or inaccuracies such as under-reporting HFMD incidence may have been present in the passive surveillance data, which potentially leads to underestimation of the effects [56]. Fourth, only partial available and measurable meteorological and air pollution variables were considered as confounders in the models. There may have been additional potential confounders or external factors that are worth considering in further research.

## Conclusions

This study revealed a significant two-dimension relationship (i.e. exposure–response and lag-response) between several air pollution indicators and HFMD incidence. However, the specific effects, including RRs and ARs, differ depending on the air pollution variable and topographic division. Children living in the Sichuan Basin are more vulnerable to PM_10_-related HFMD compared with SO_2_ and NO_2_. The health effects of O_3_ were much greater than those of other air pollutants, particularly in basin and mountain regions. These findings could furnish local authorities with more evidence to determine key air pollutants and regions for devising and implementing targeted interventions.

### Supplementary Information

**Additional file 1.**

## Data Availability

The data that generated and analyzed during the current study are available from Sichuan CDC but restricted to apply to the availability of these data, which were used under license for the current study, and so are not publicly available. But data are available from the corresponding author upon reasonable request with permission of Sichuan CDC.

## Abbreviations

HFMD: Hand, foot, and mouth disease
DLNM: Distributed lag non-linear model
RR: Relative risk
AR: Attributable risk
AF: Attributable fraction
AN: Attributable number
CI: Confidence interval
