# Supplementary material for: How multiple air pollutants affect hand, foot, and mouth disease incidence in children: assessing effect modification by geographical context in multicity of Sichuan, southwest China
Source: BMC Public Health. 2024 Jan 23;24:263. doi: 10.1186/s12889-023-17484-9 (PMC10804470; doi:10.1186/s12889-023-17484-9)
Supplement: Supplementary file 1 — Additional file 1. [file 12889_2023_17484_MOESM1_ESM.docx]

**How multiple air pollutants affect hand, foot, and mouth disease incidence in children: assessing effect modification by geographical context in multicity of Sichuan, southwest China**

**Figures**

**Fig. S1. City-specific relationship of HFMD counts with air pollution in 21 prefecture-level cities in Sichuan Province.**

**Fig. S2. Contour plots of the city-specific relationship between the risk of HFMD and PM_10_ at different time lags.**

**Fig. S3. Contour plots of the city-specific relationship between the risk of HFMD and SO_2_ at different time lags.**

**Fig. S4. Contour plots of the city-specific relationship between the risk of HFMD and NO_2_ at different time lags.**

**Fig. S5. Contour plots of the city-specific relationship between the risk of HFMD and CO at different time lags.**

**Fig. S6. Contour plots of the city-specific relationship between the risk of HFMD and O_3_ at different time lags.**


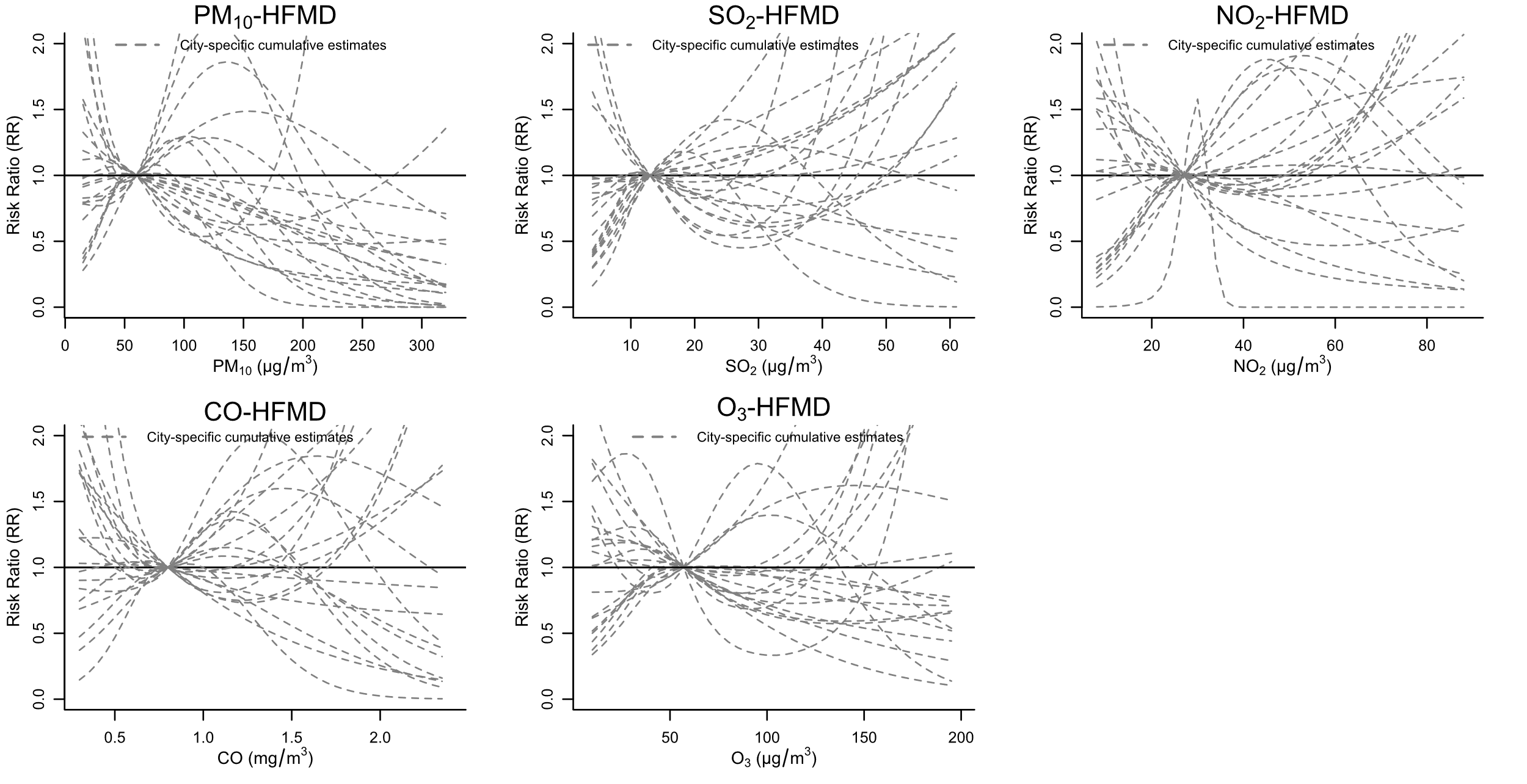


Fig. S1. City-specific relationship of HFMD counts with air pollution in 21 prefecture-level cities in Sichuan Province.


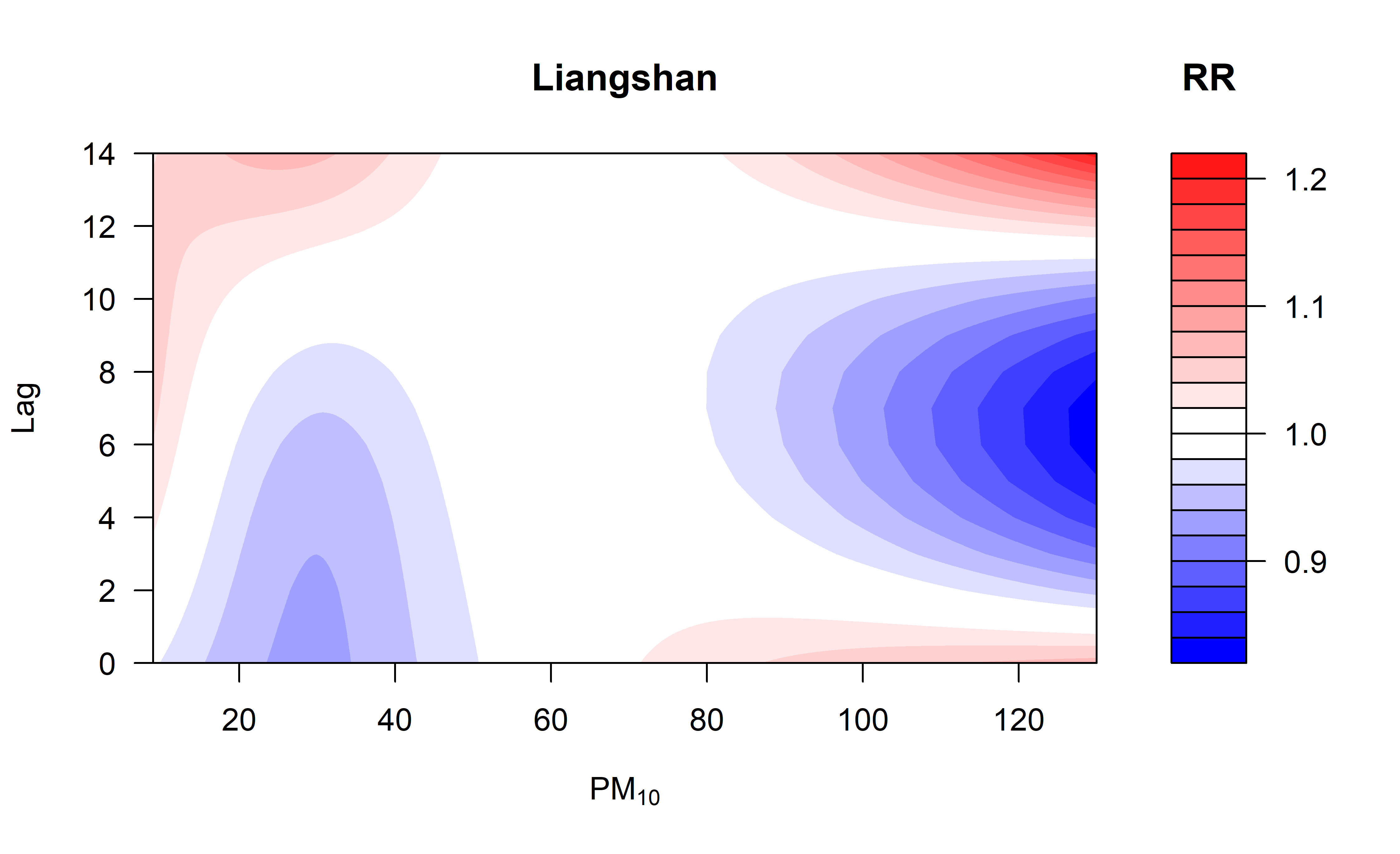

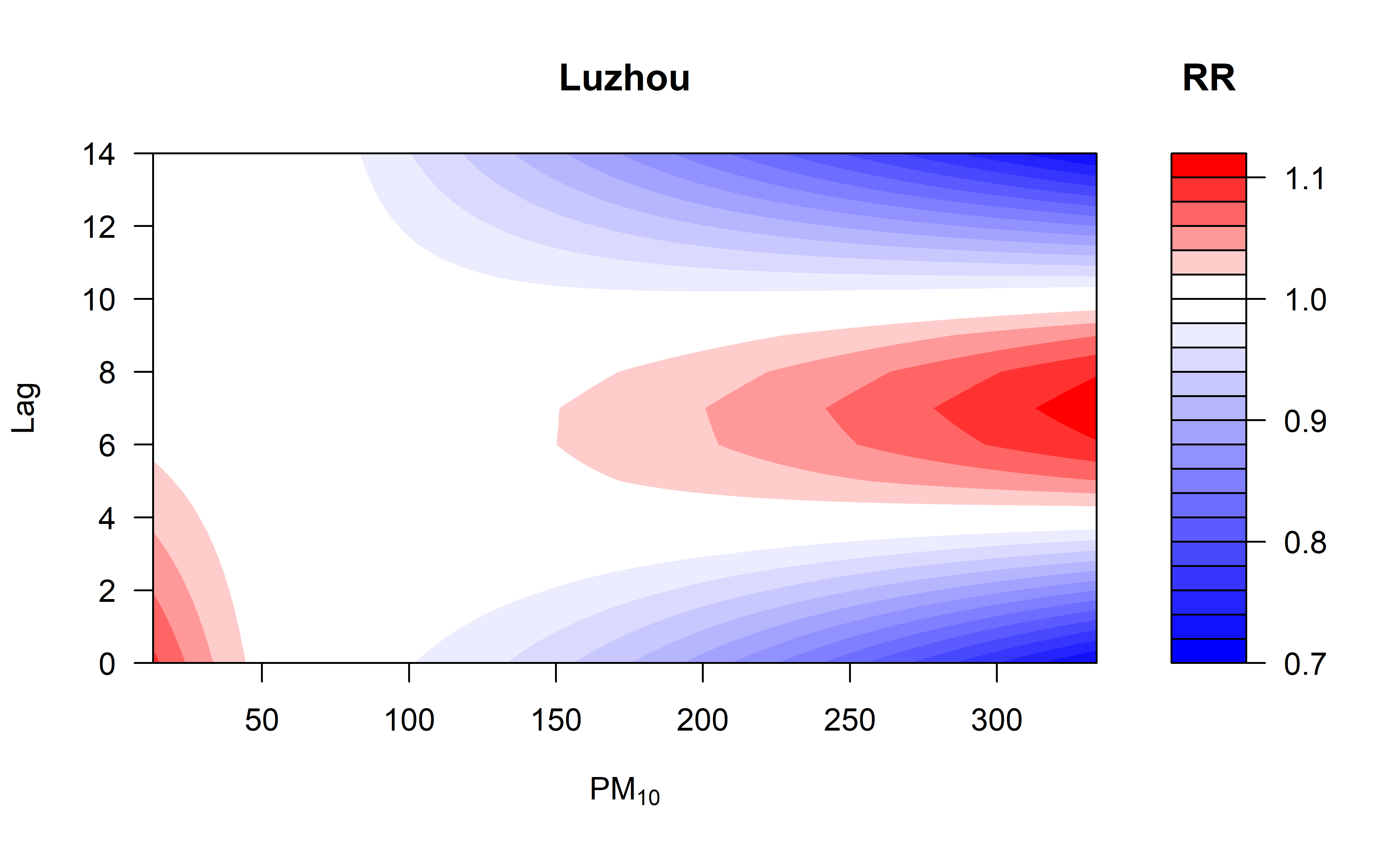

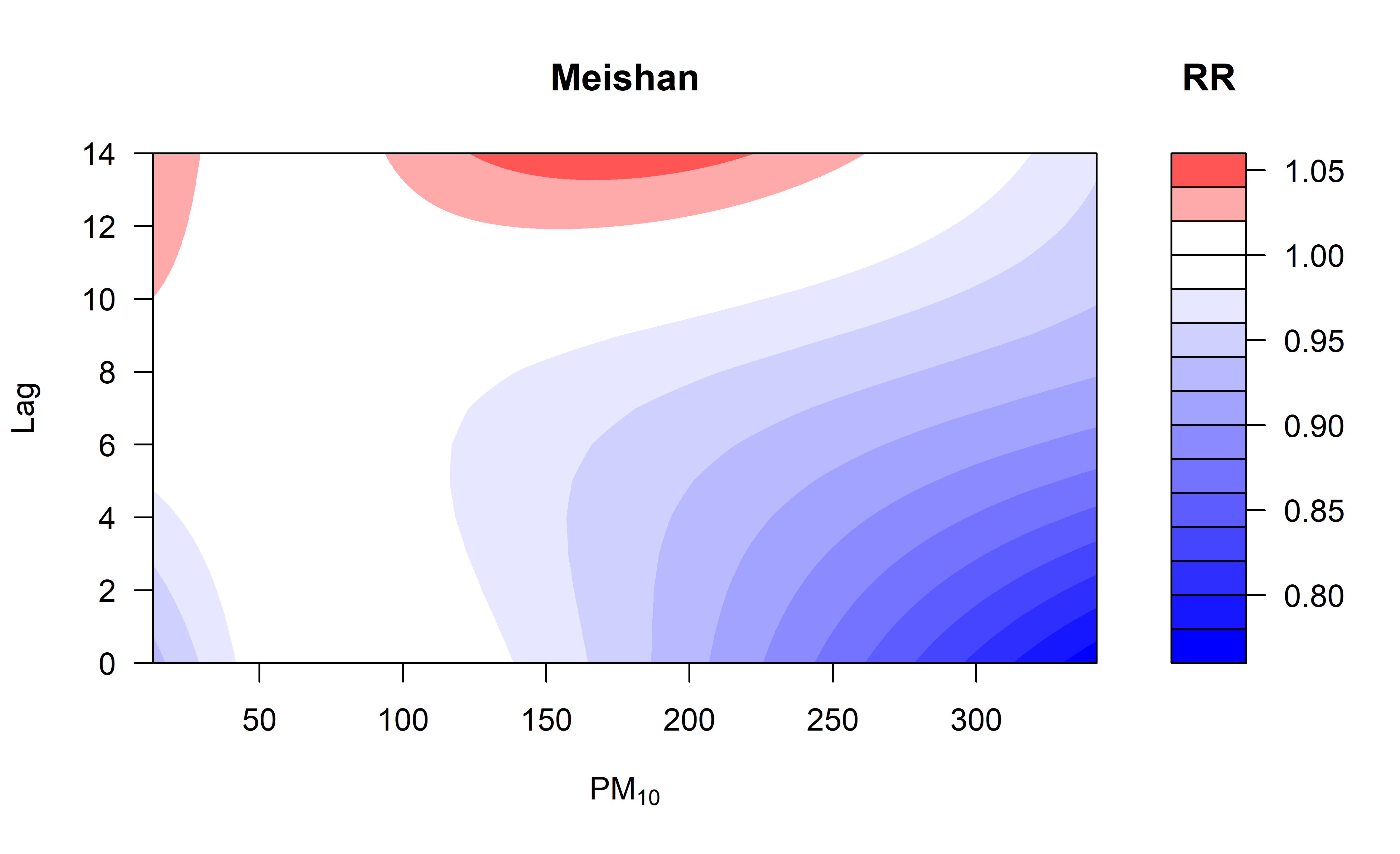

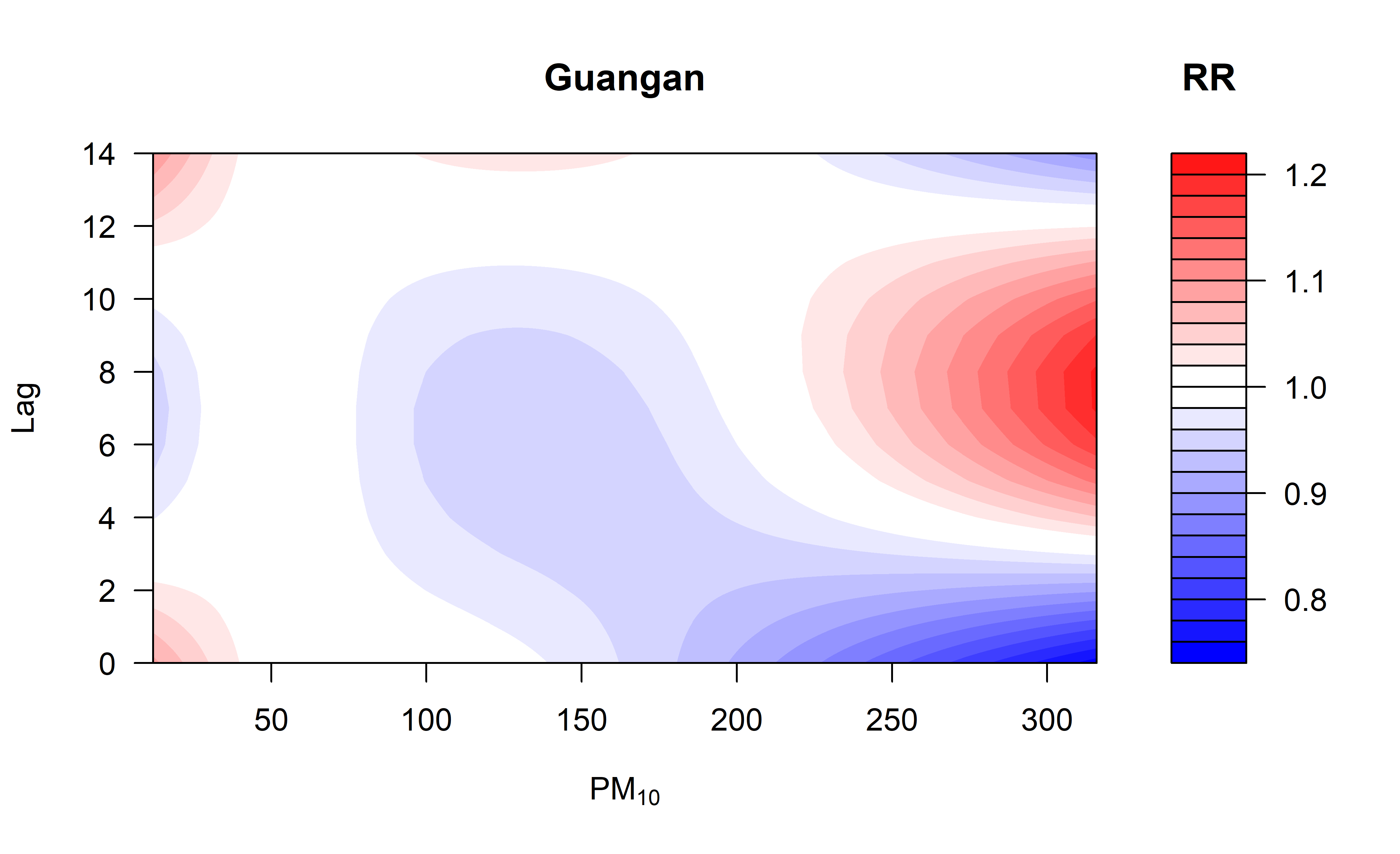

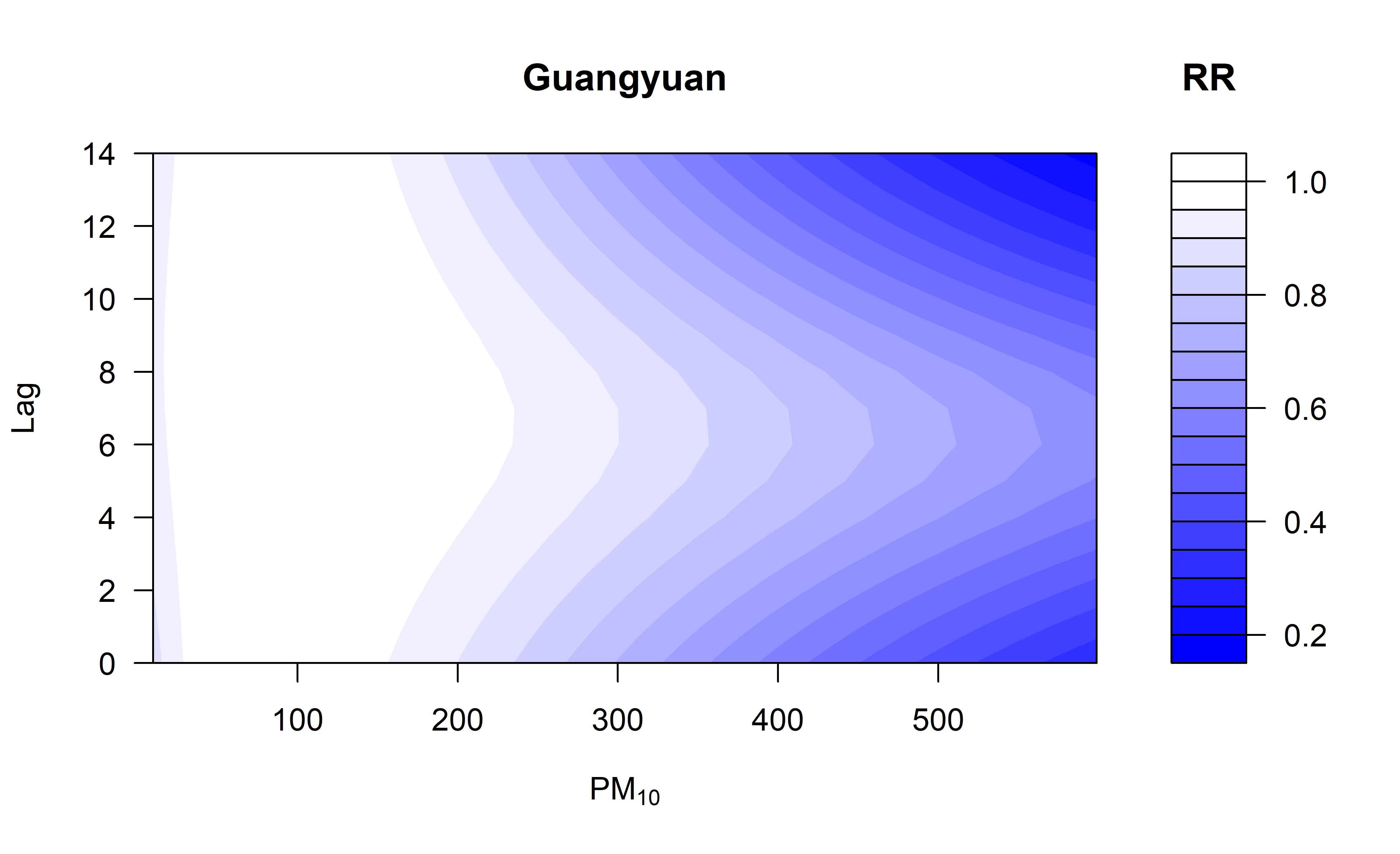

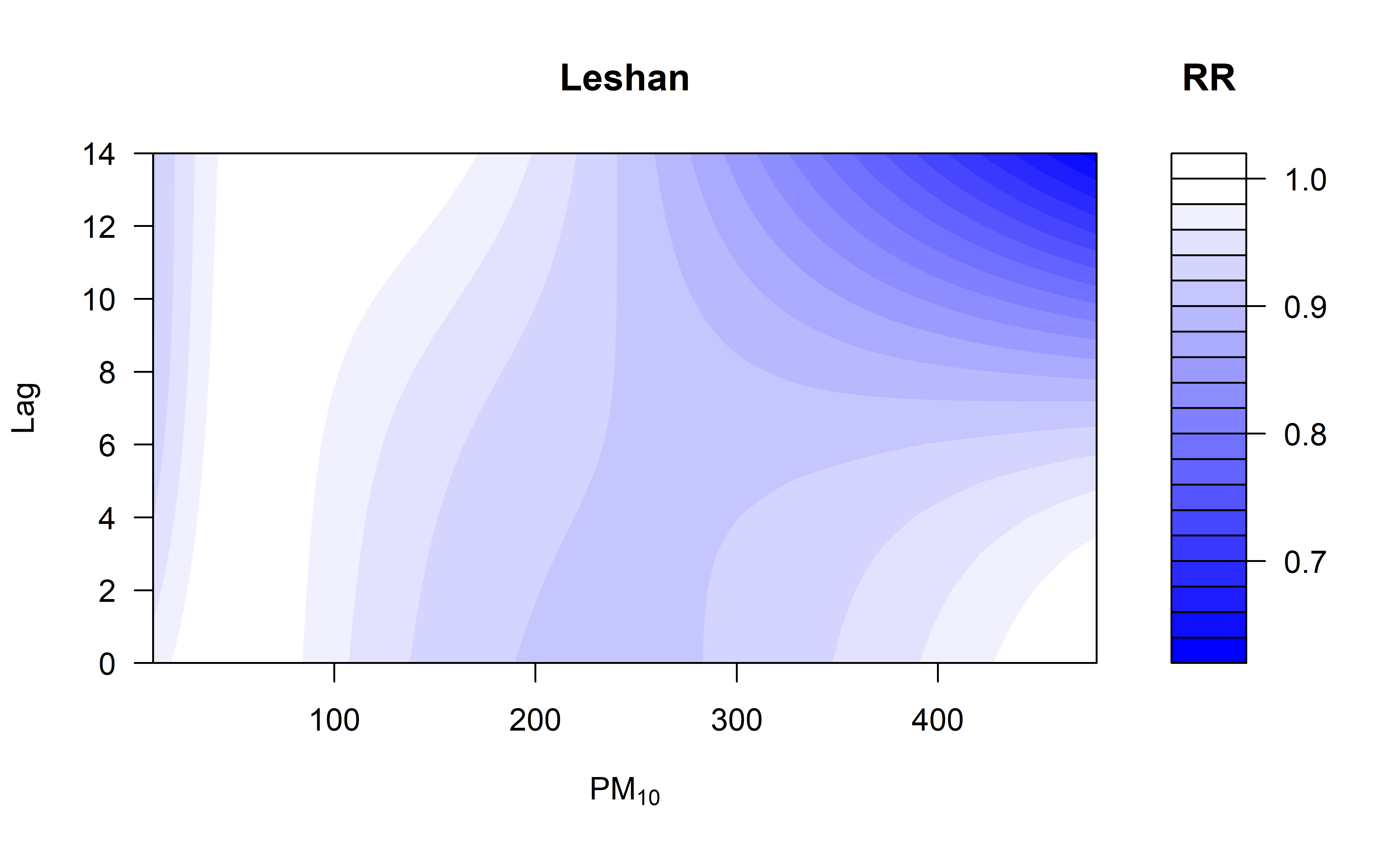

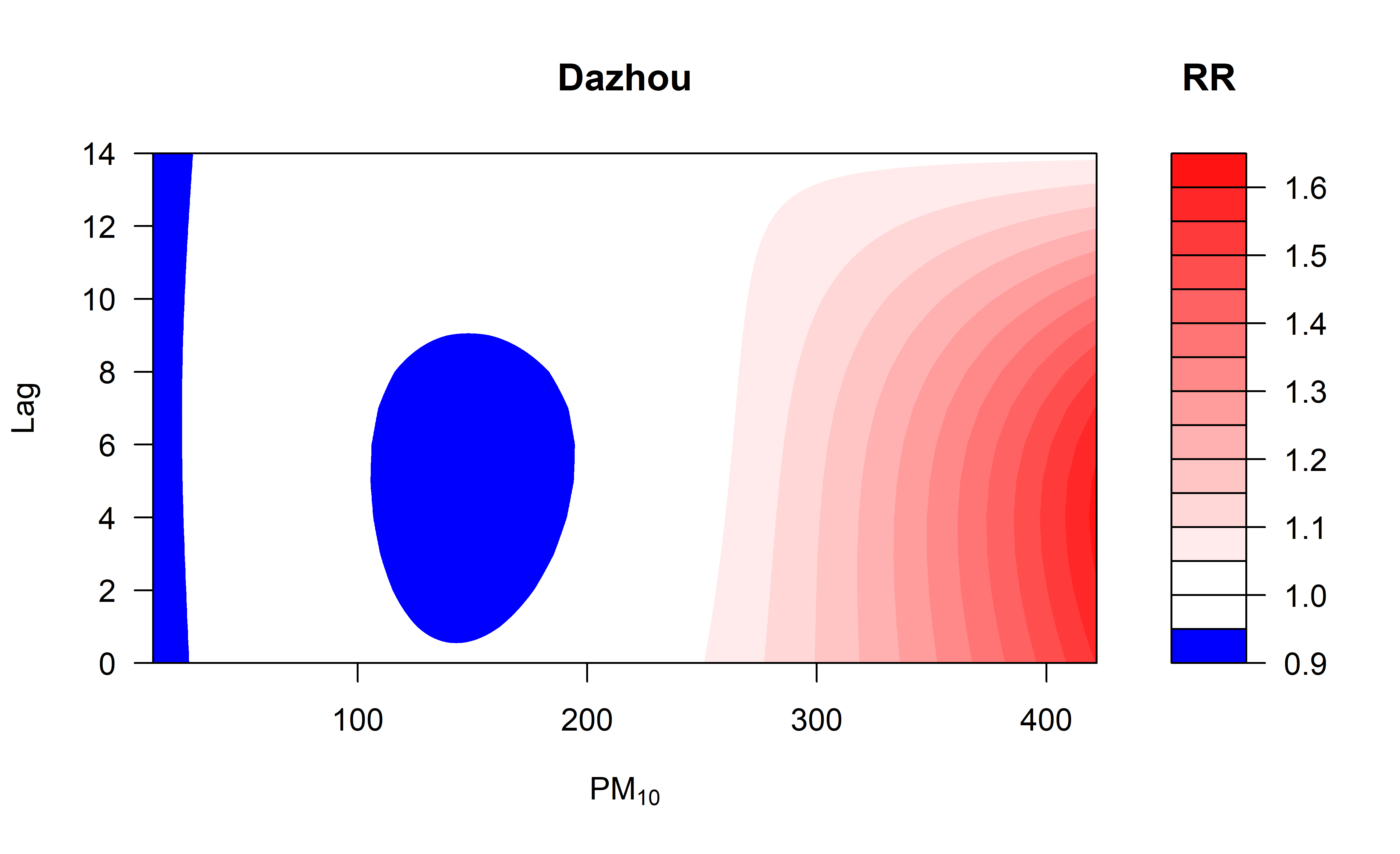

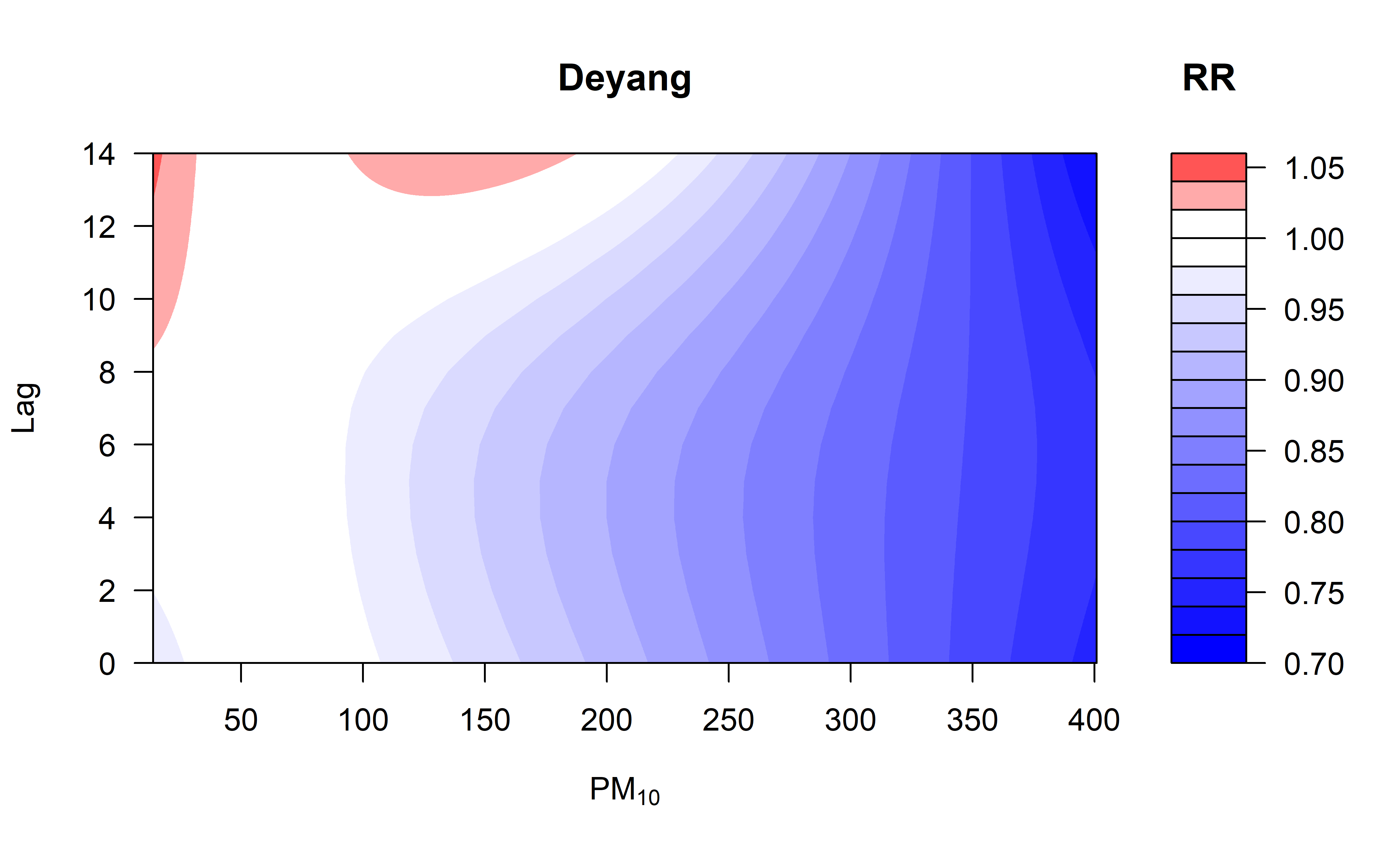

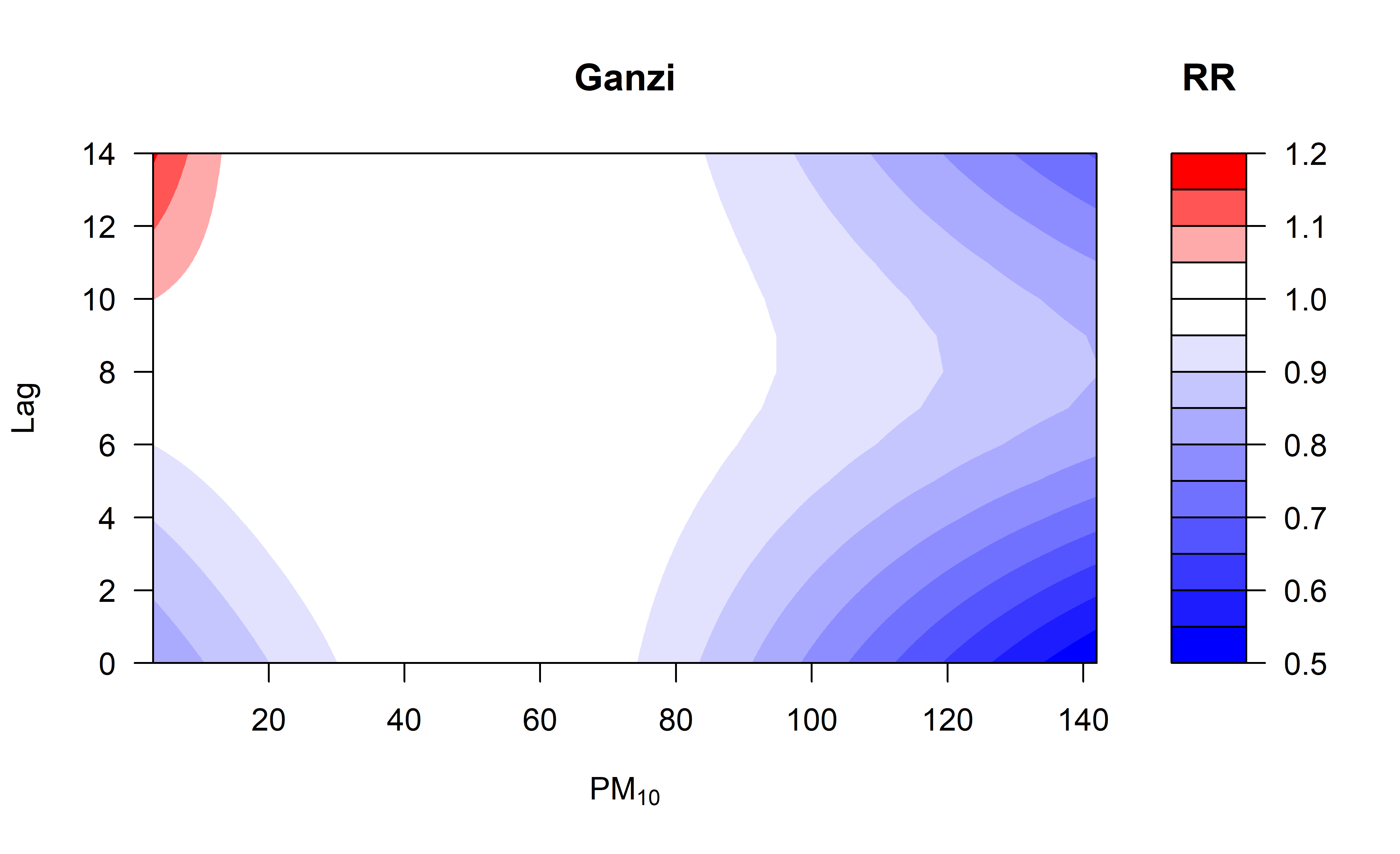

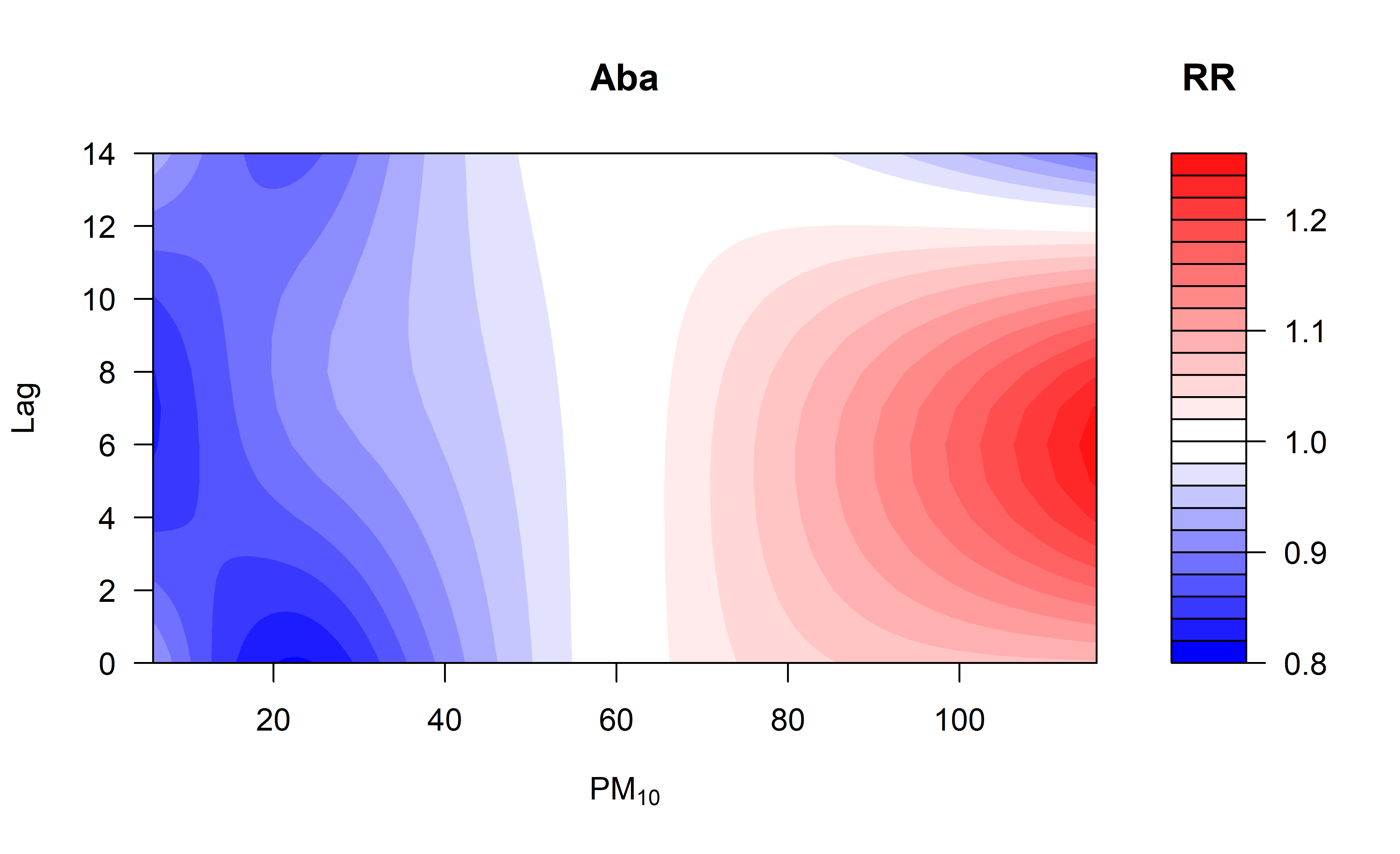

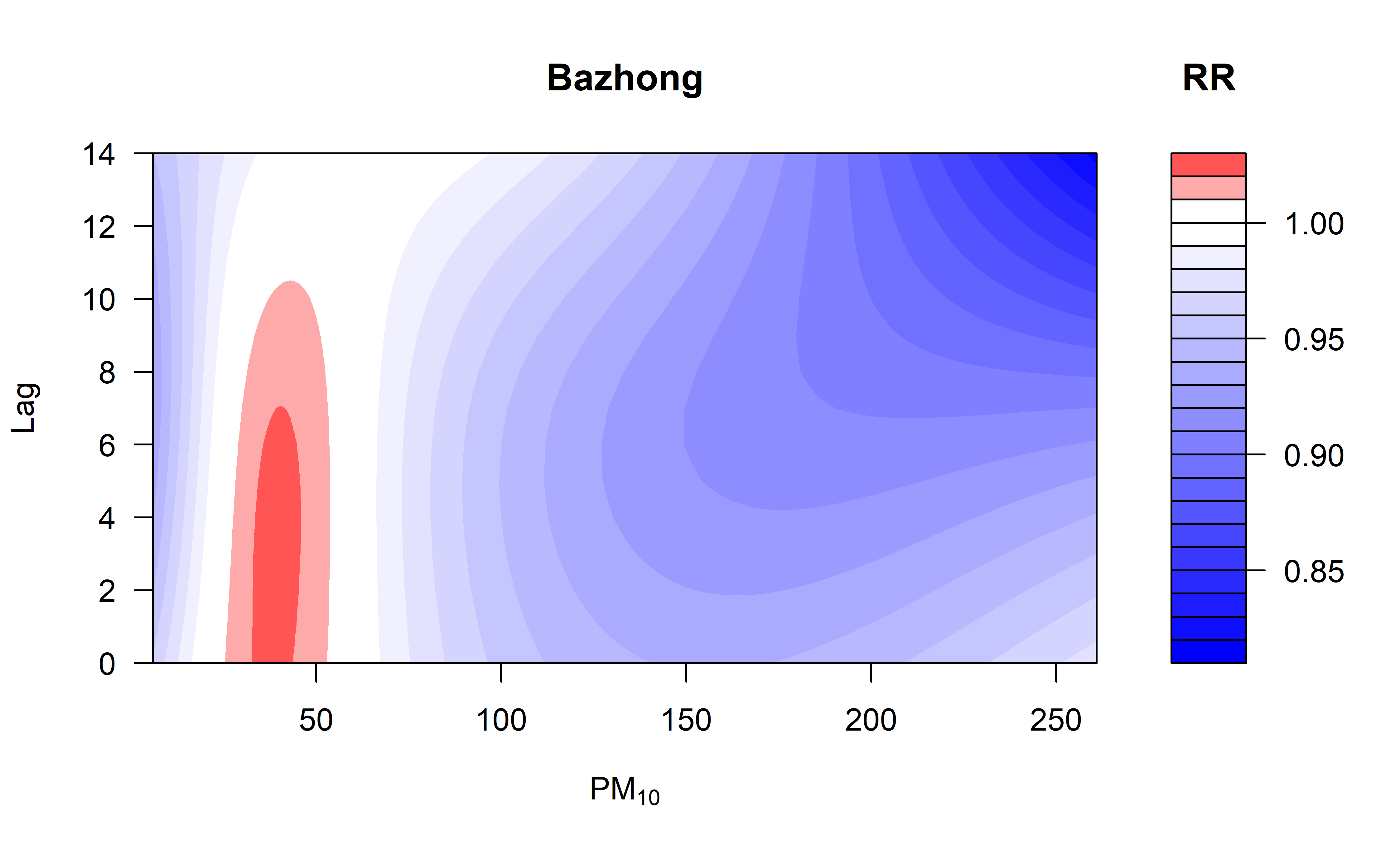

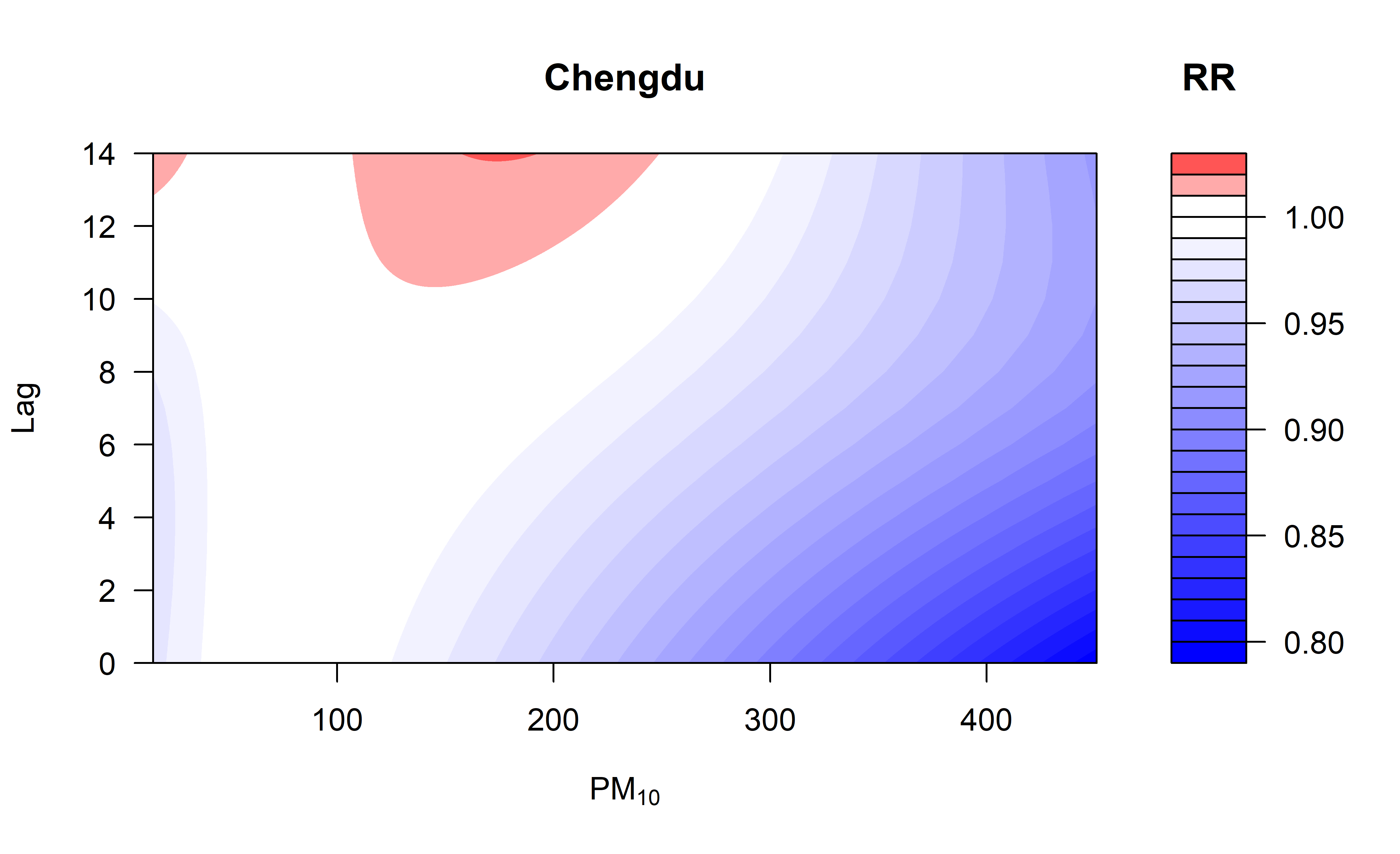

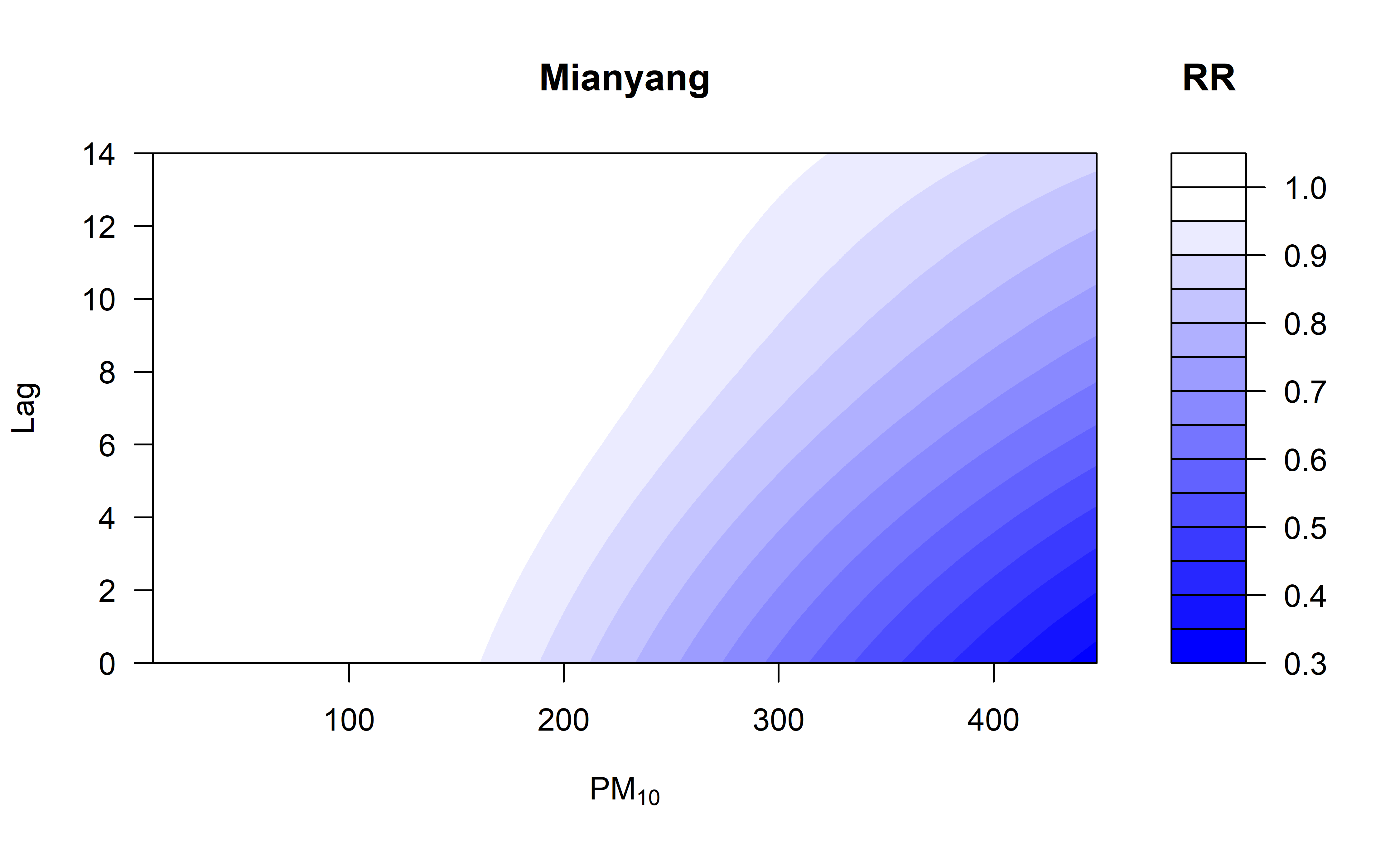

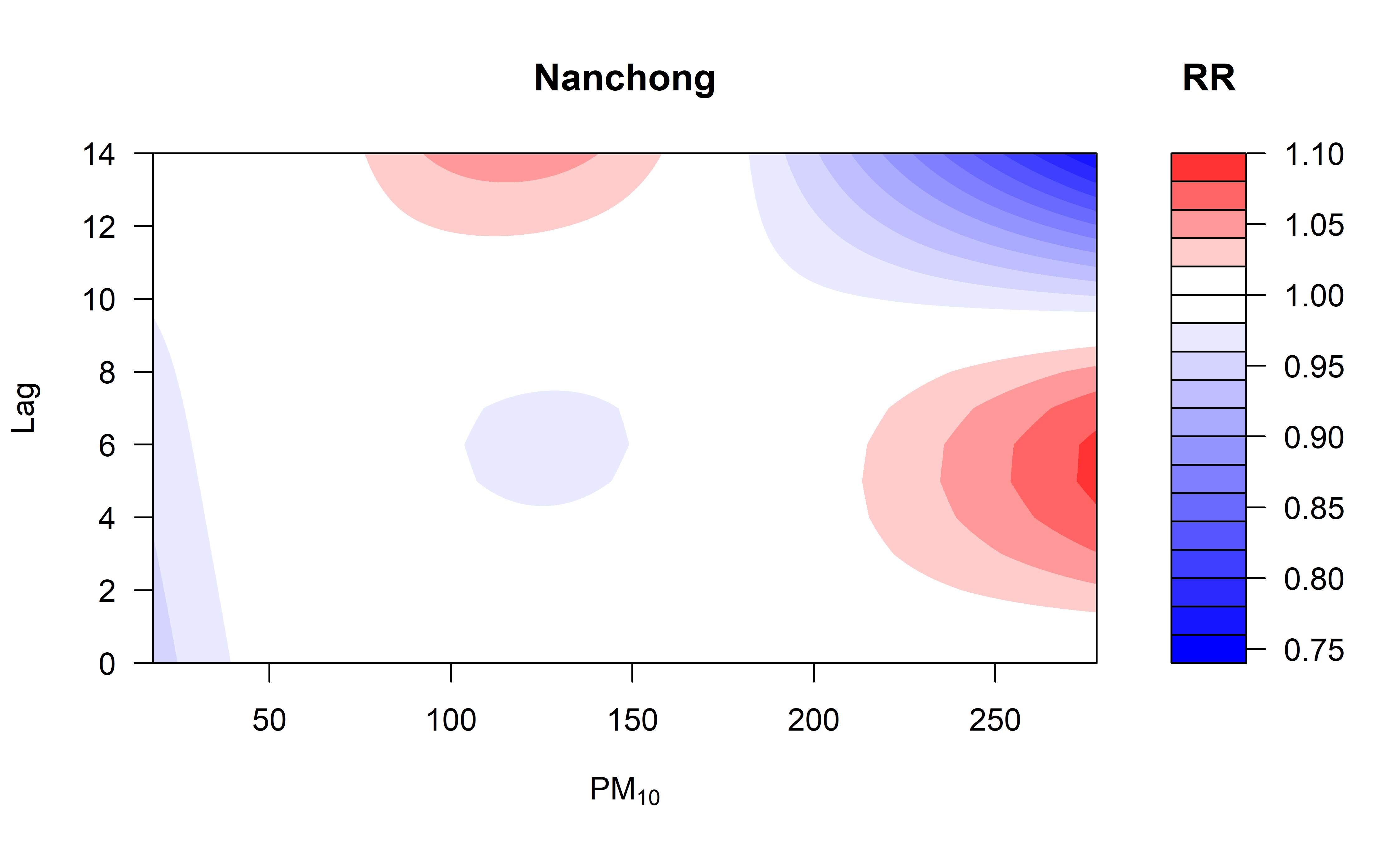

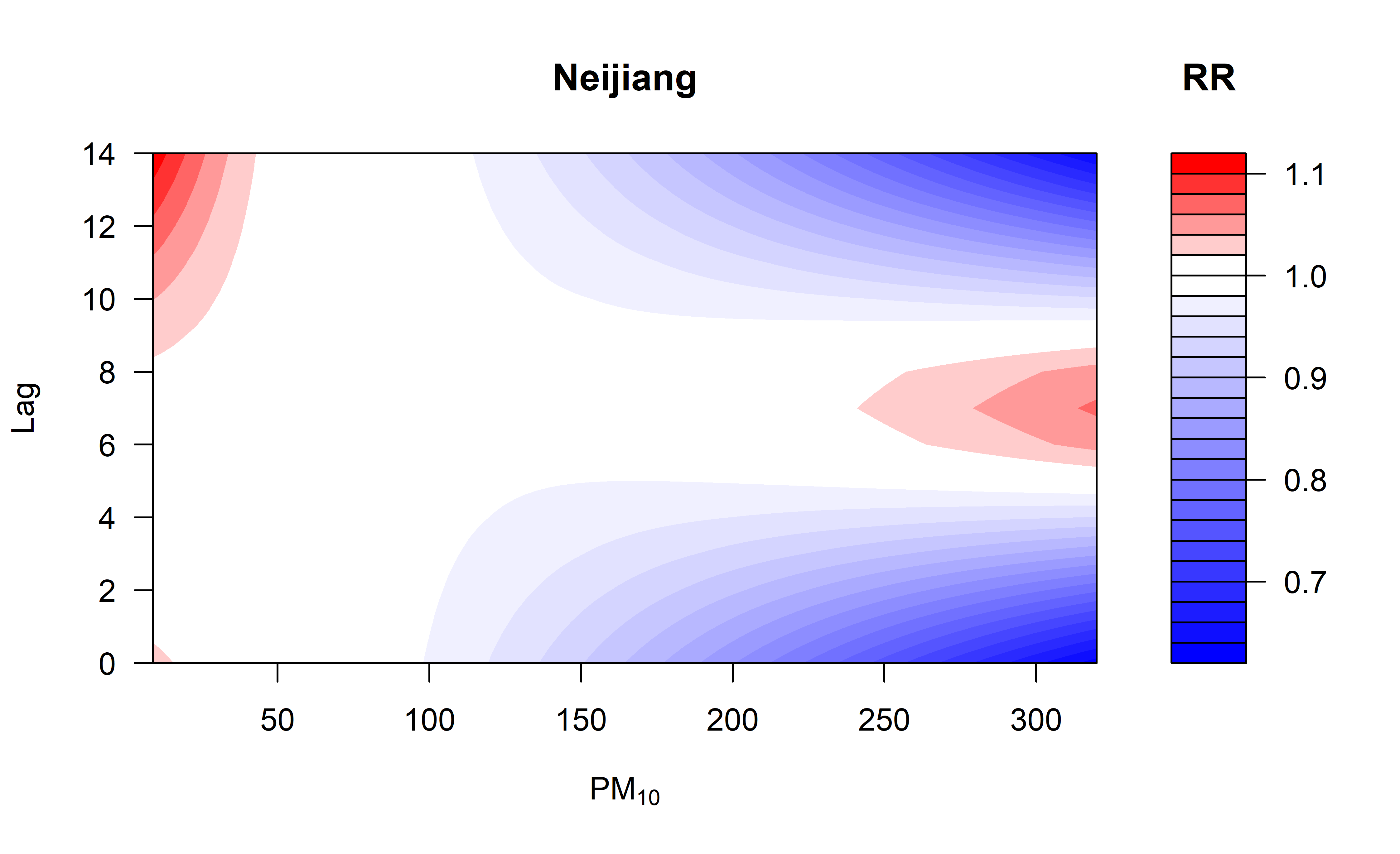

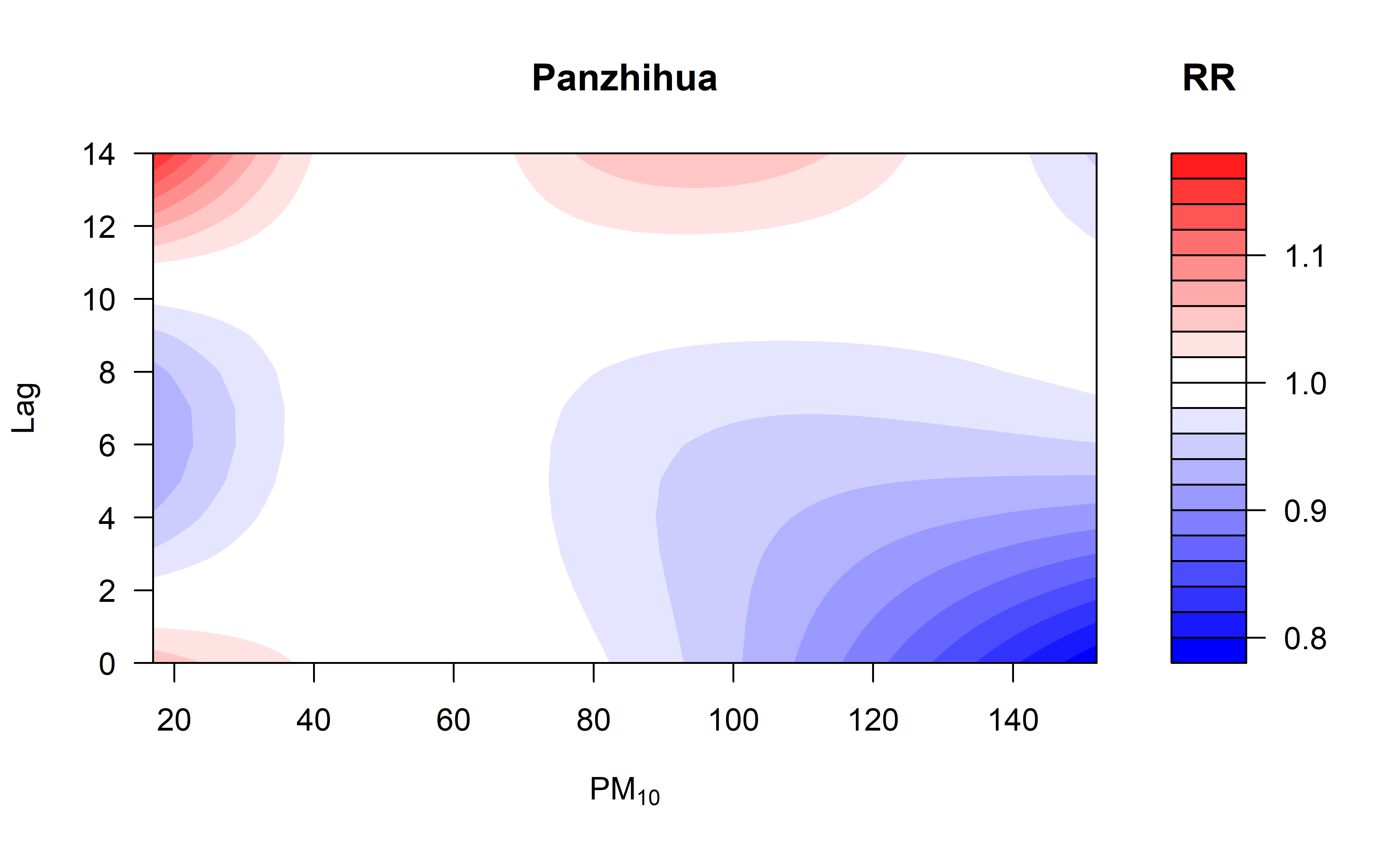

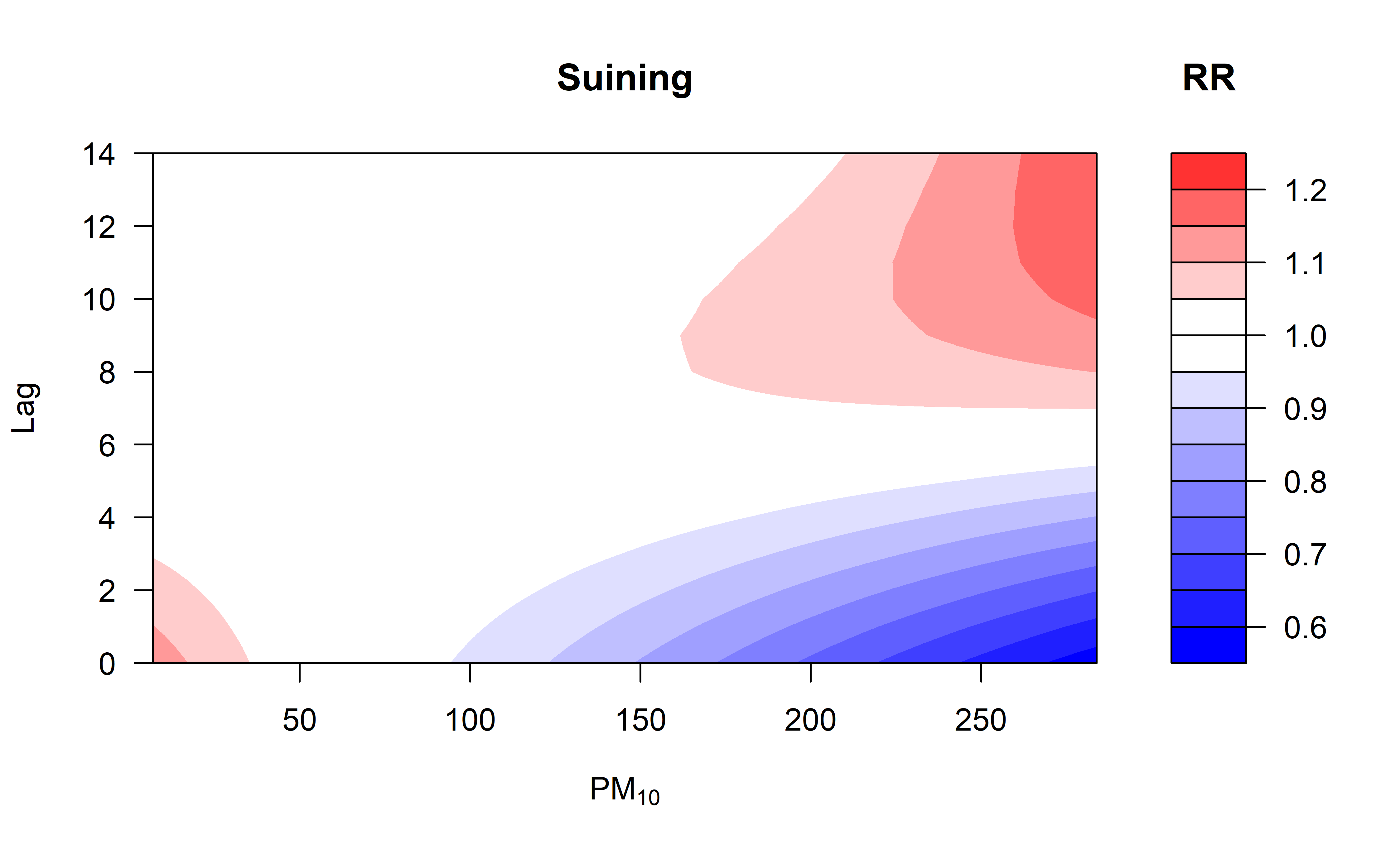

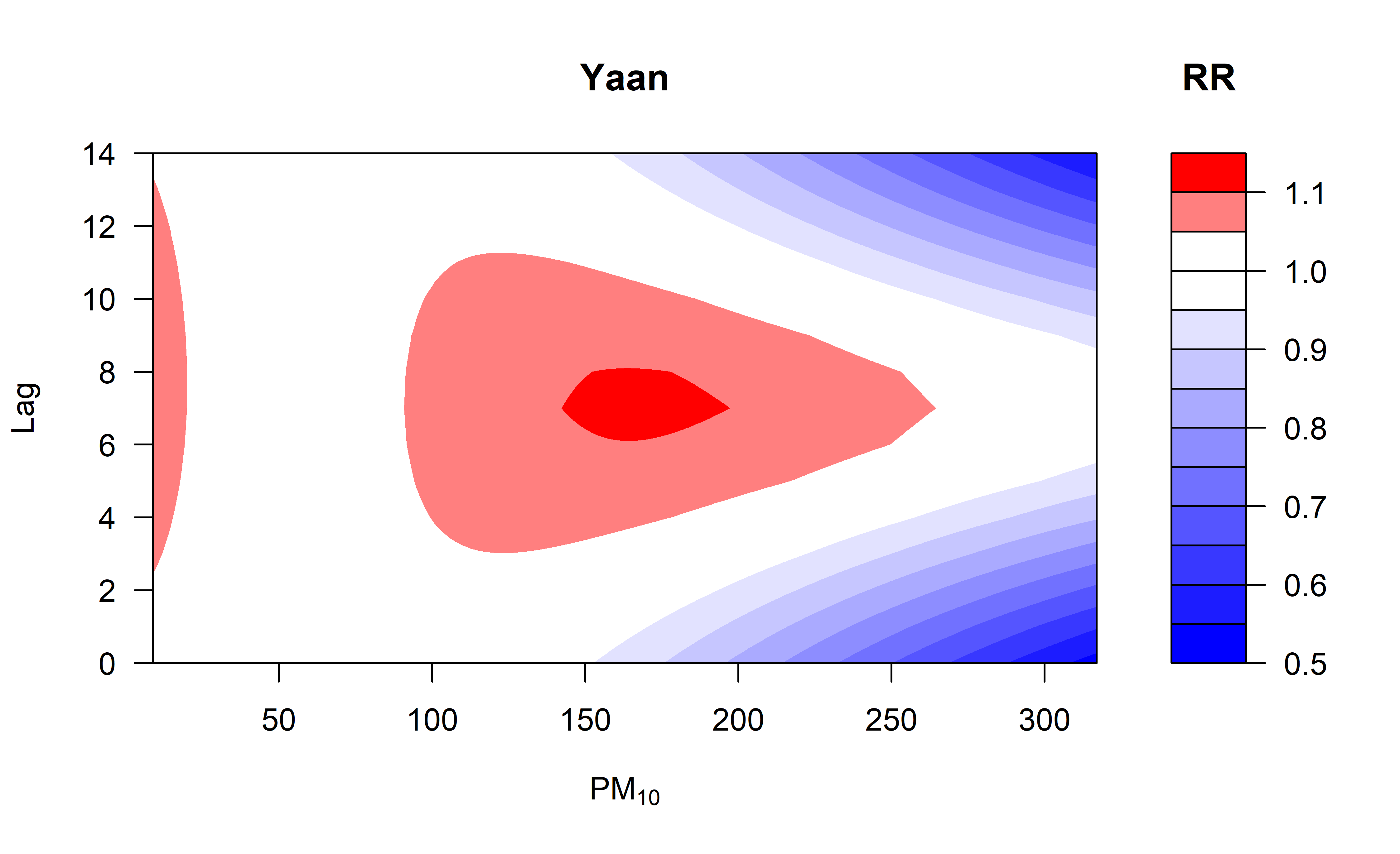

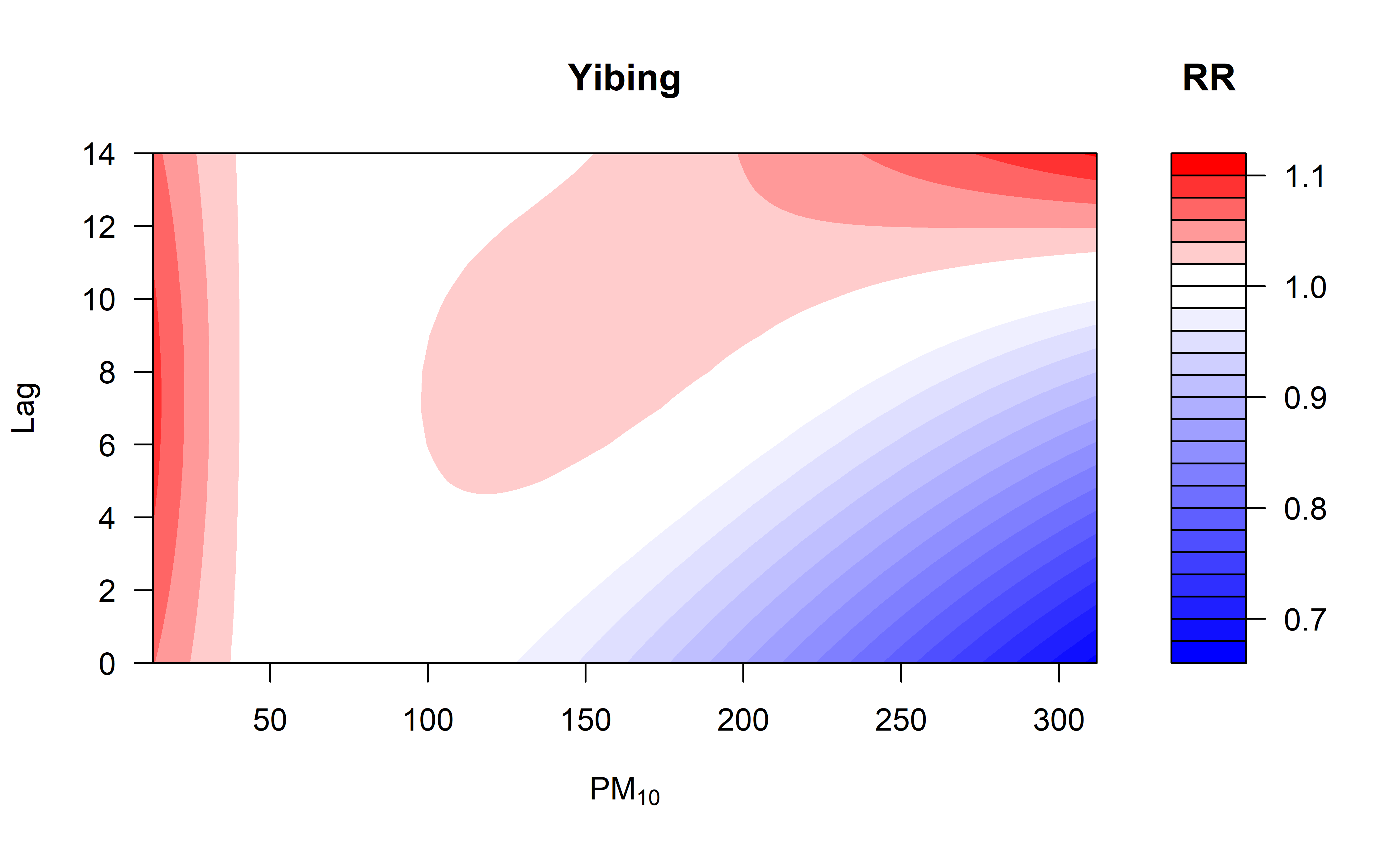

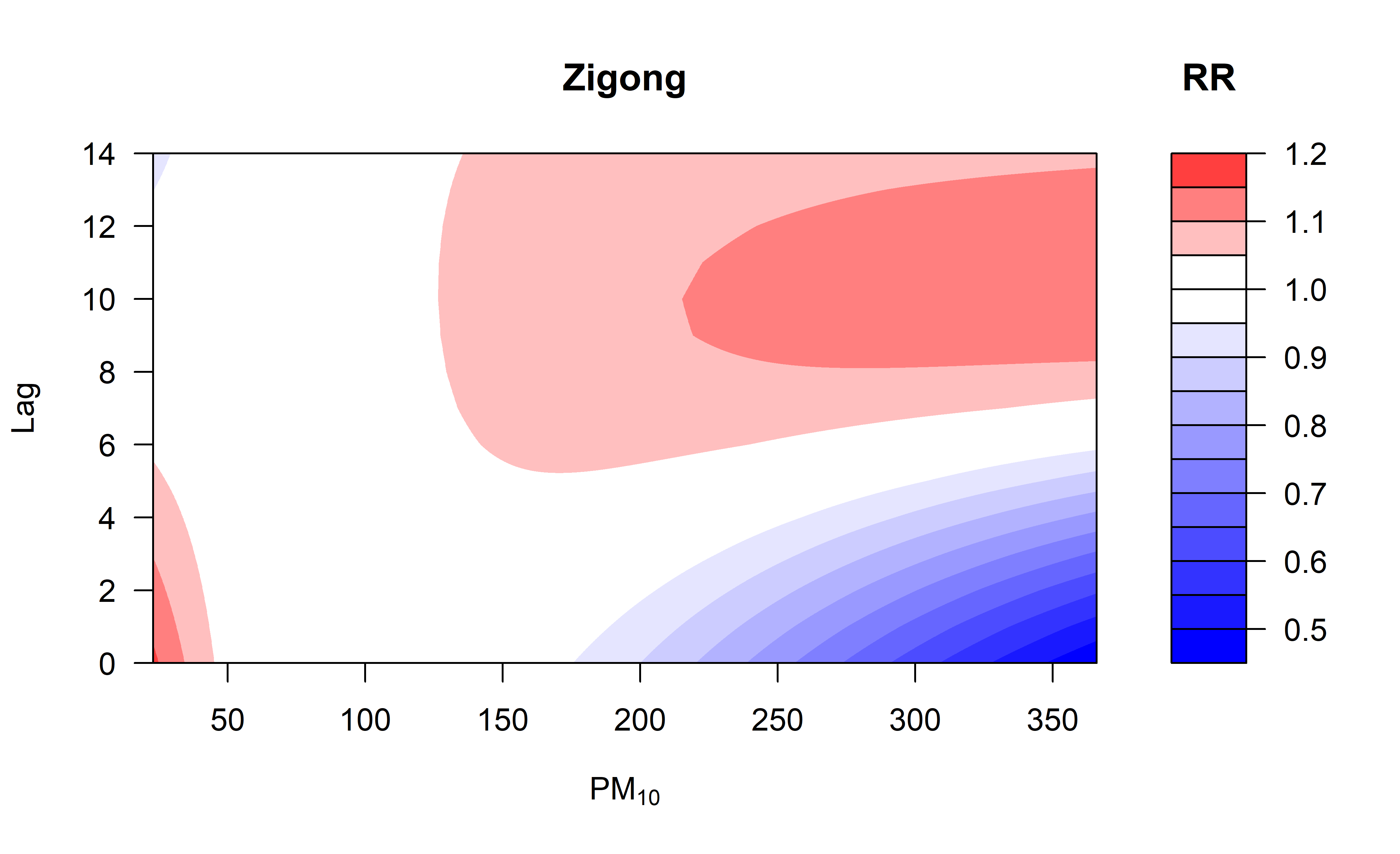

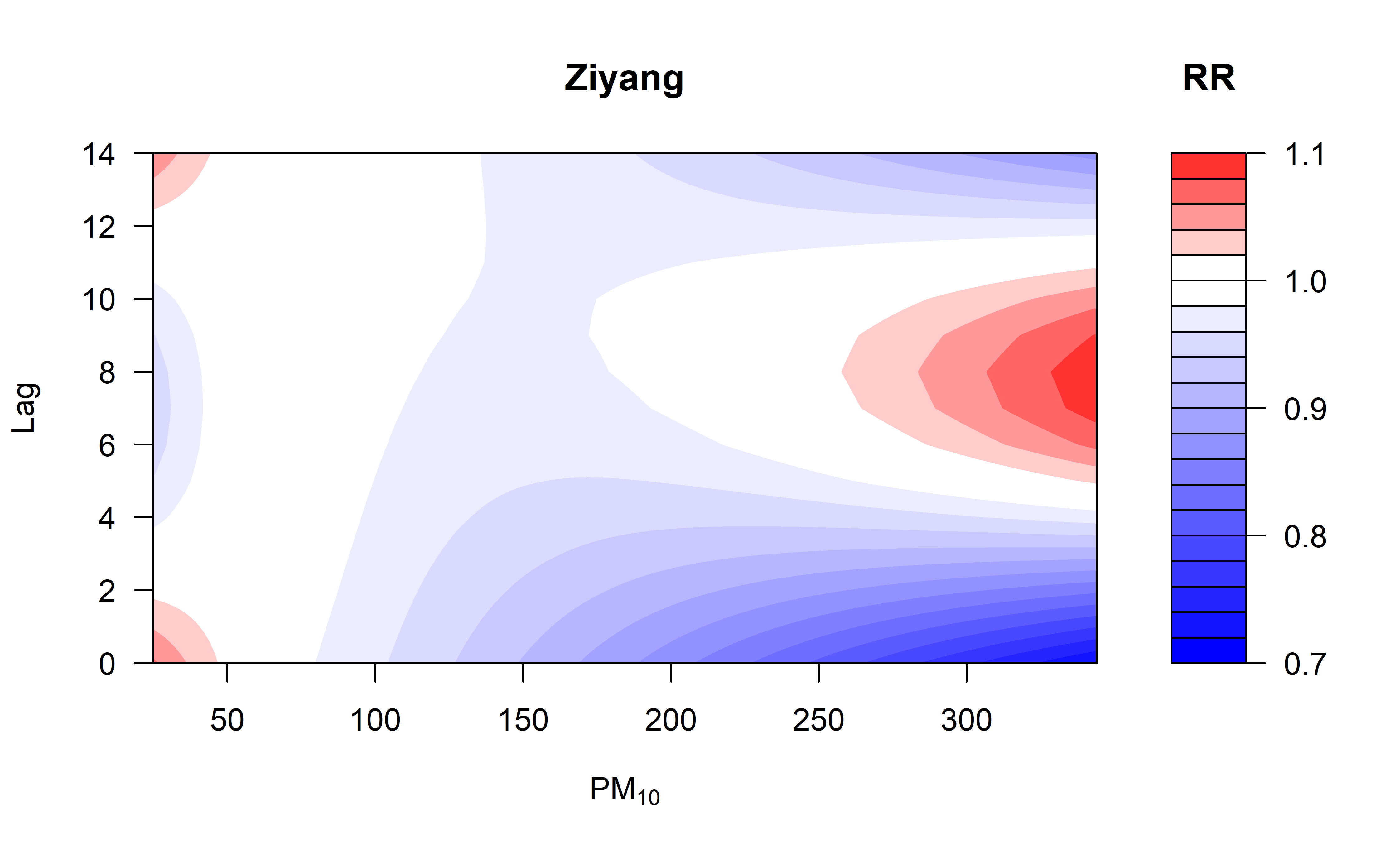


Fig. S2. Contour plots of the city-specific relationship between the risk of HFMD and PM_10_ at different time lags.


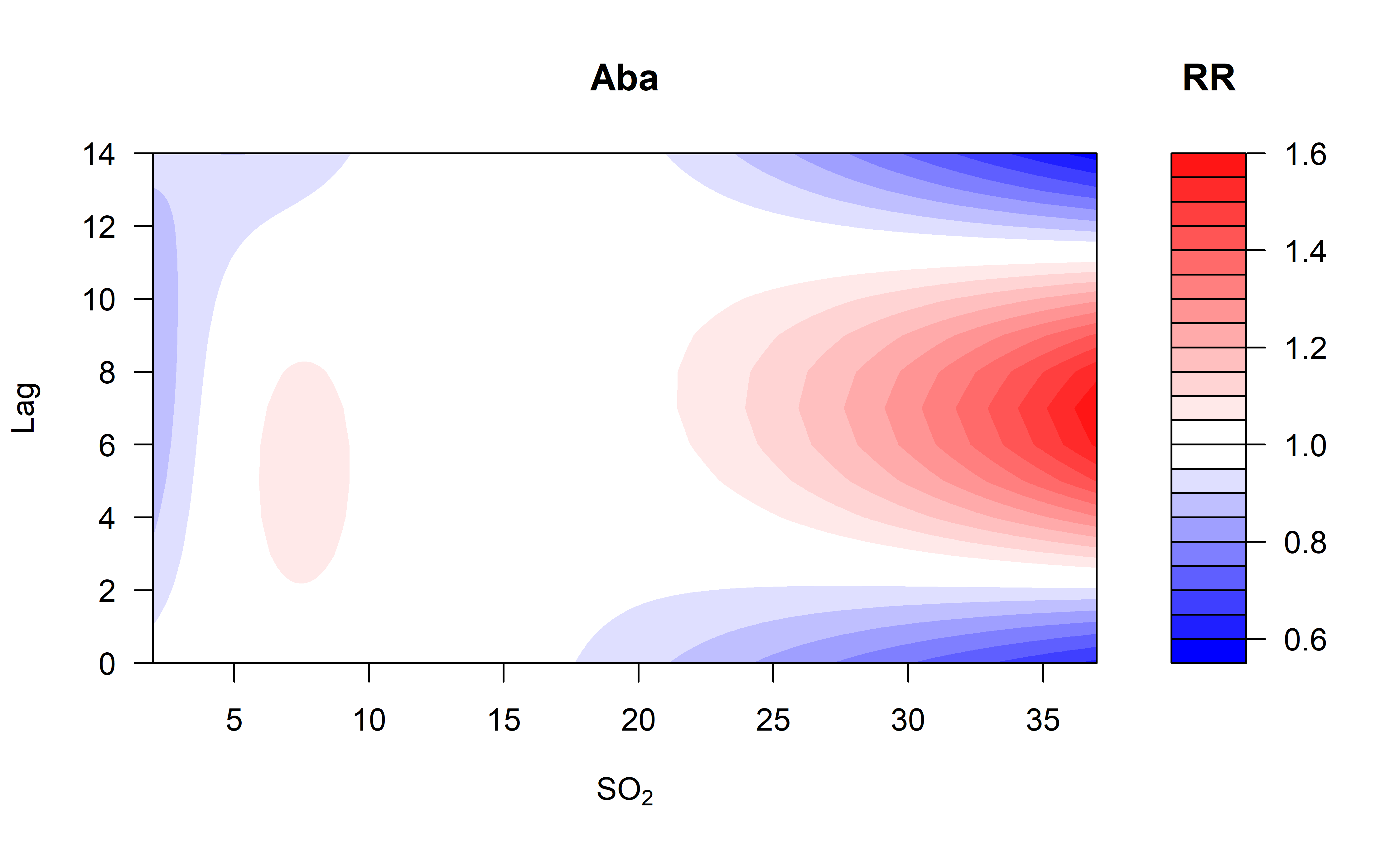

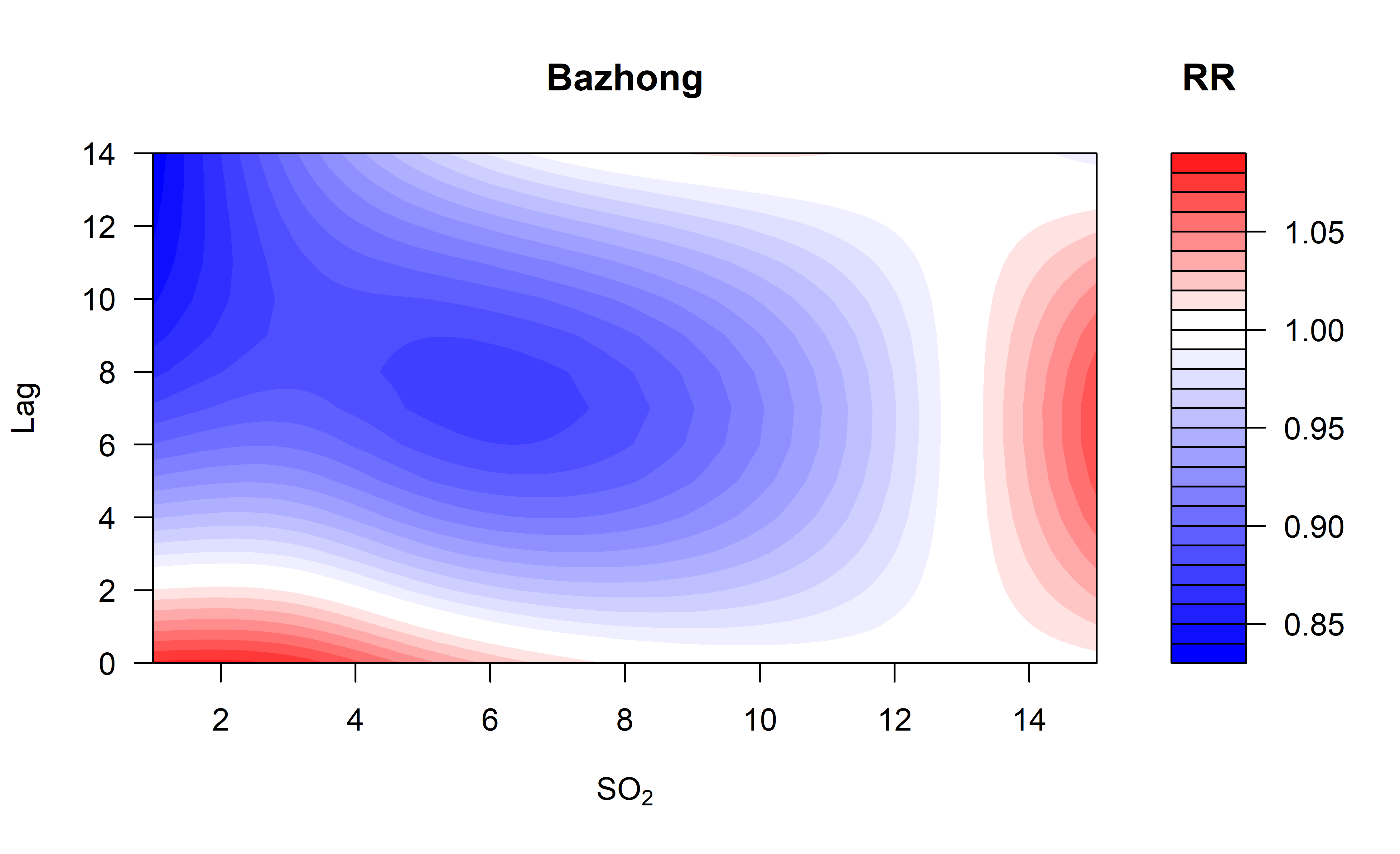

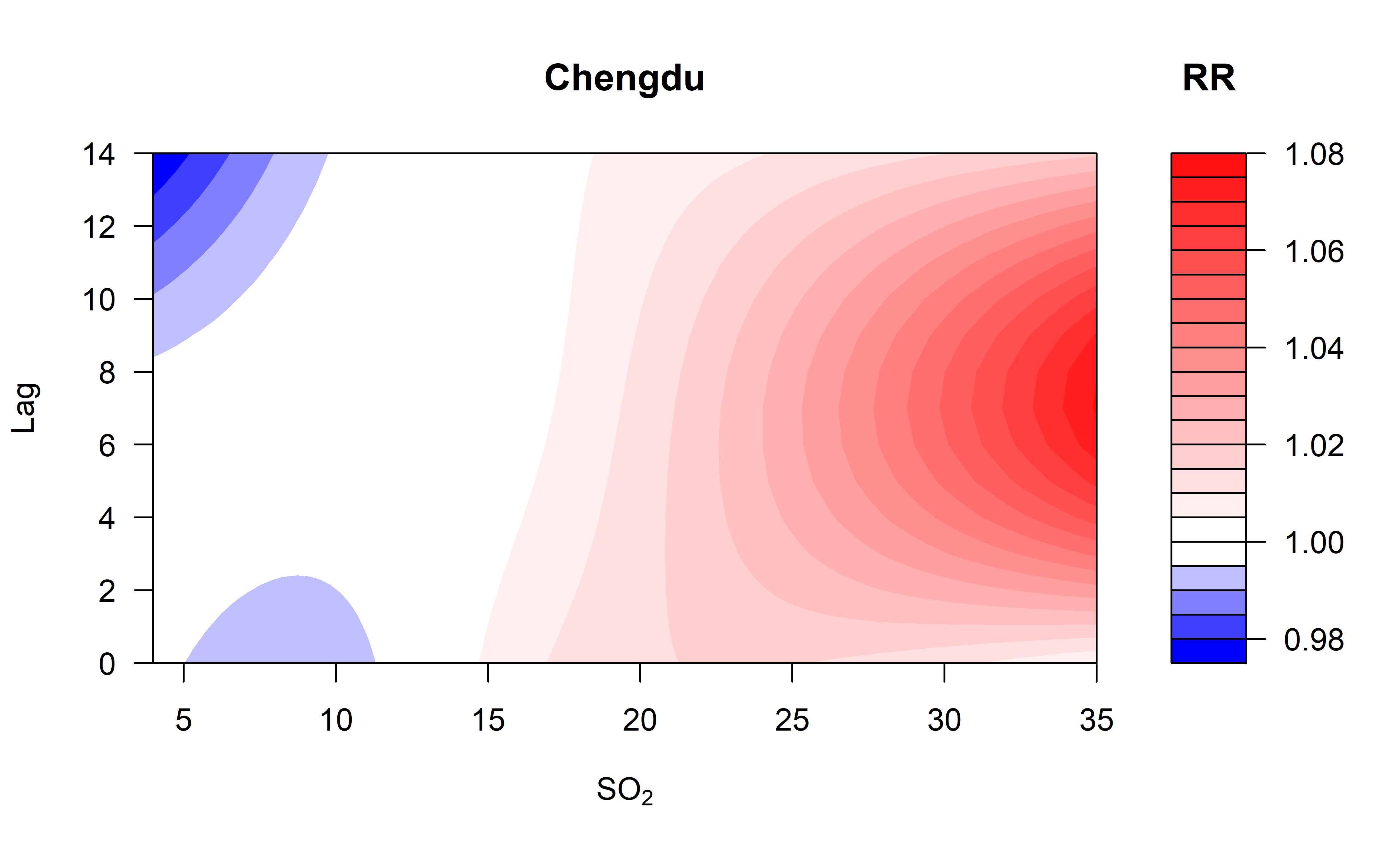

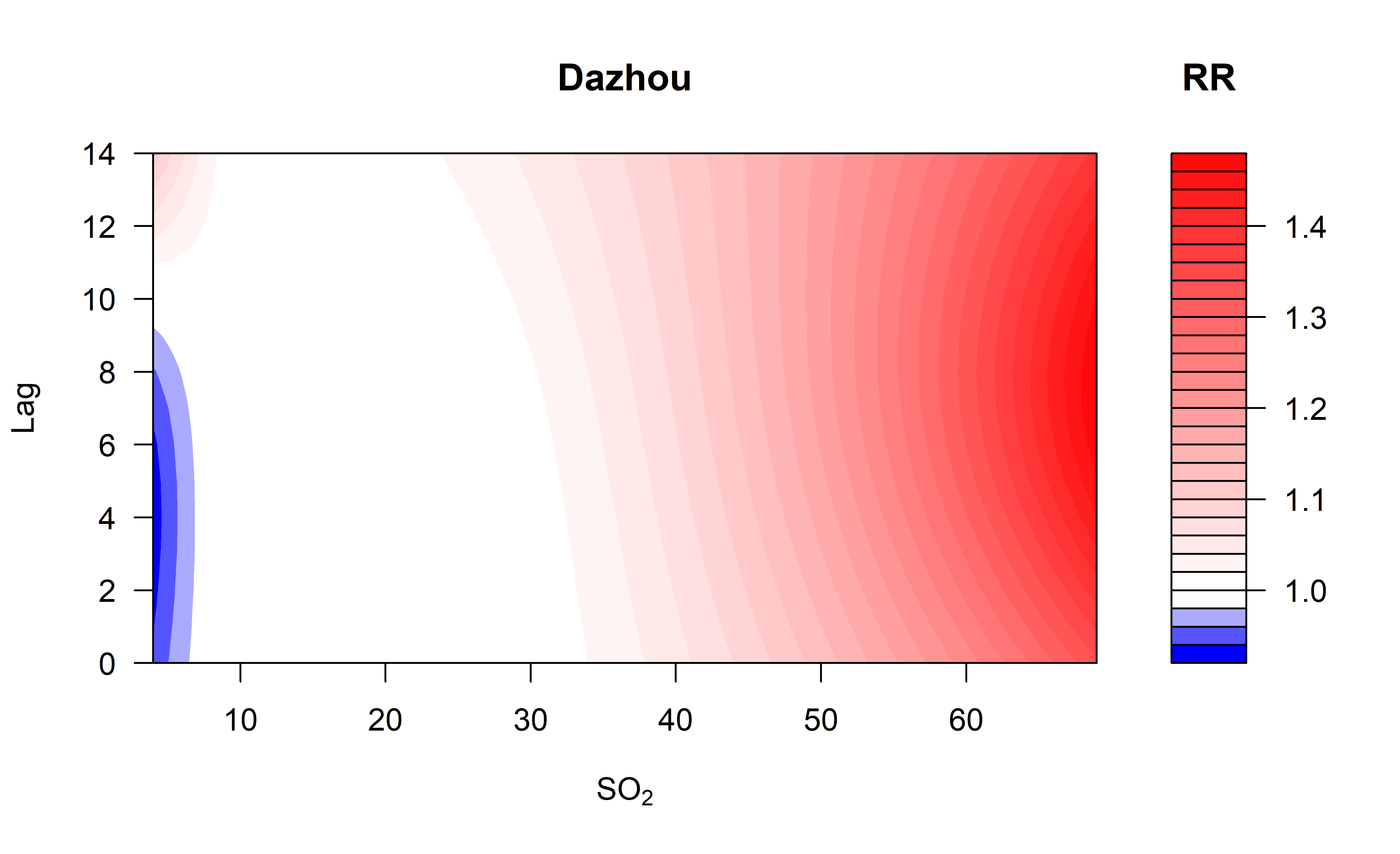

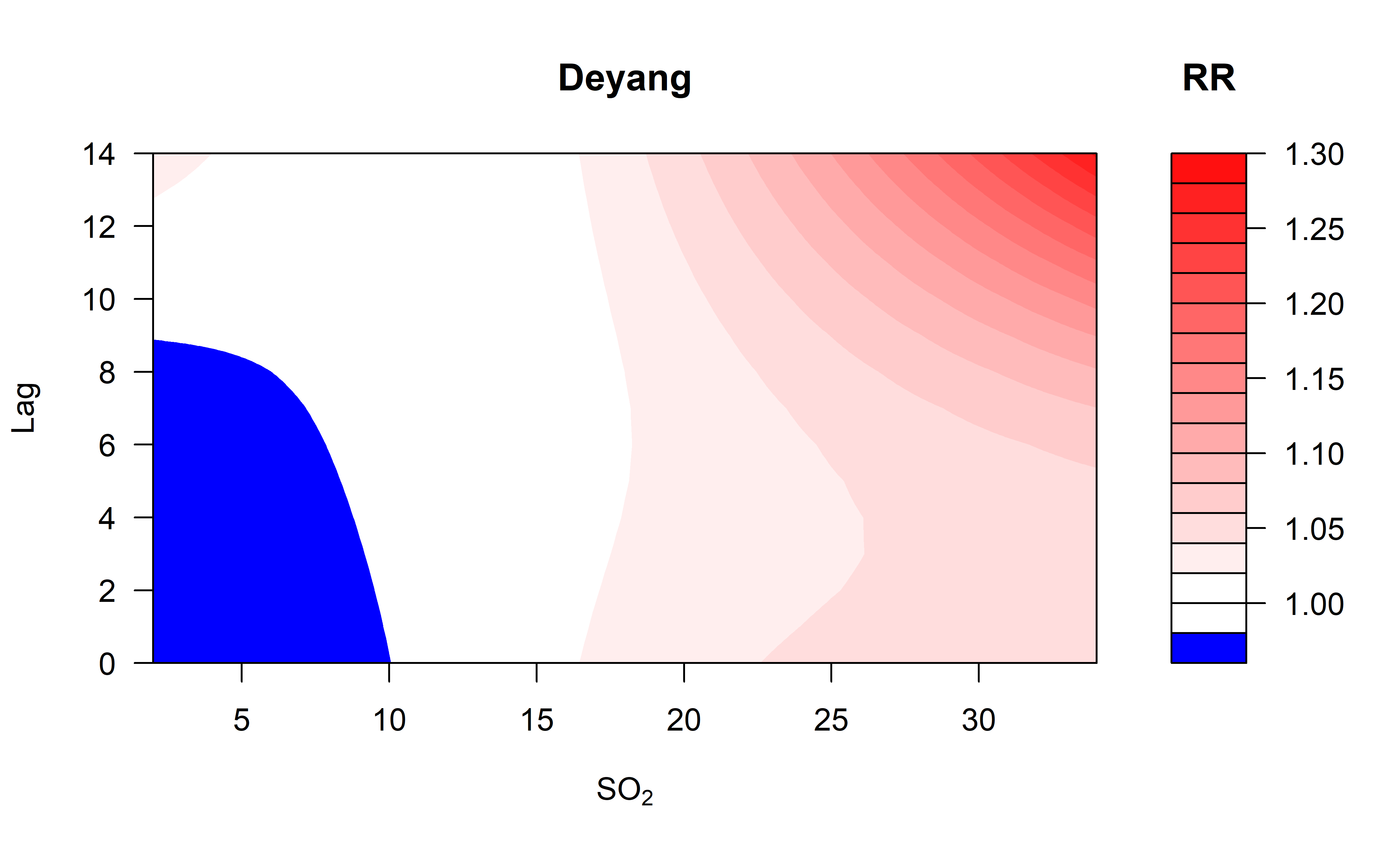

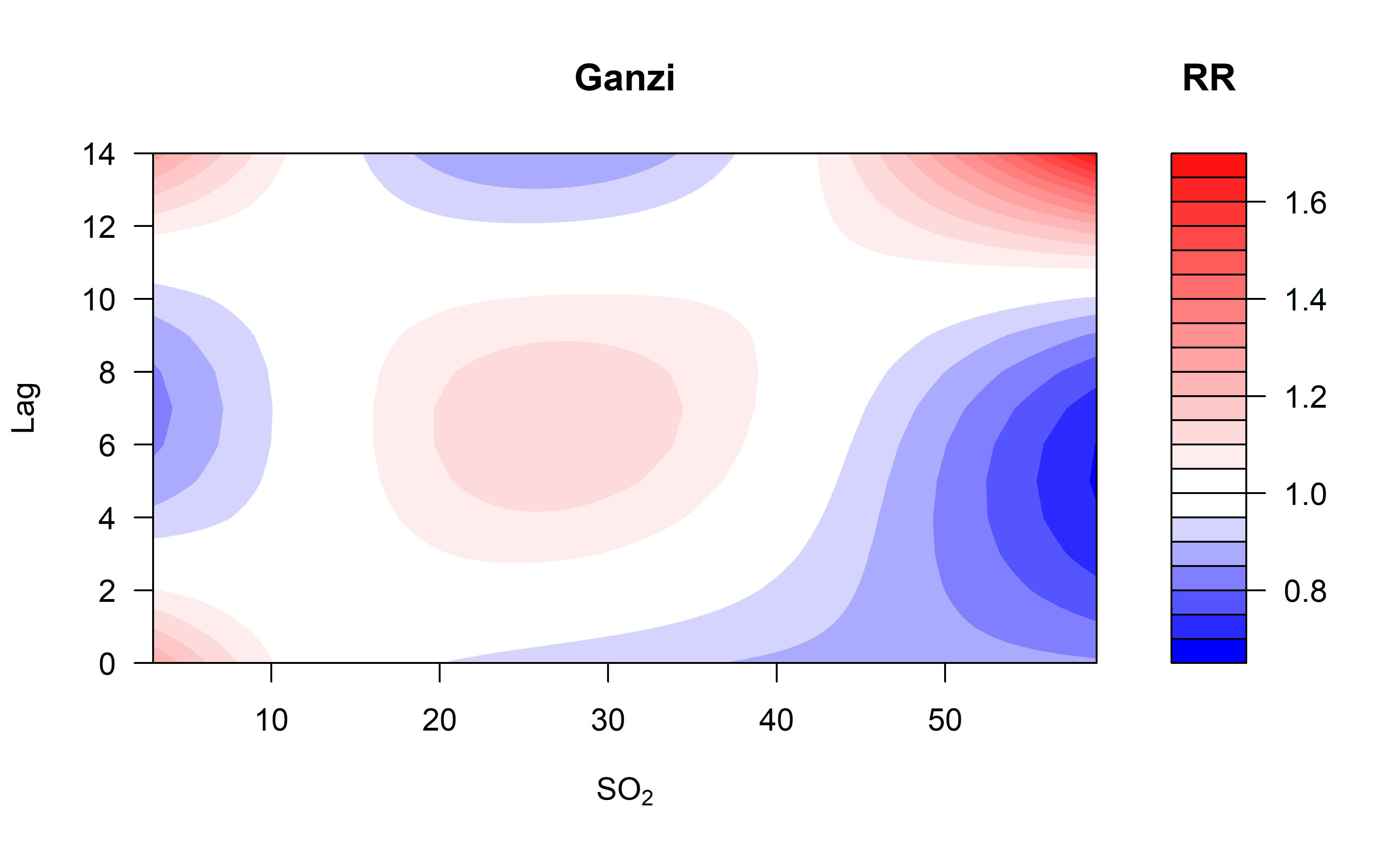

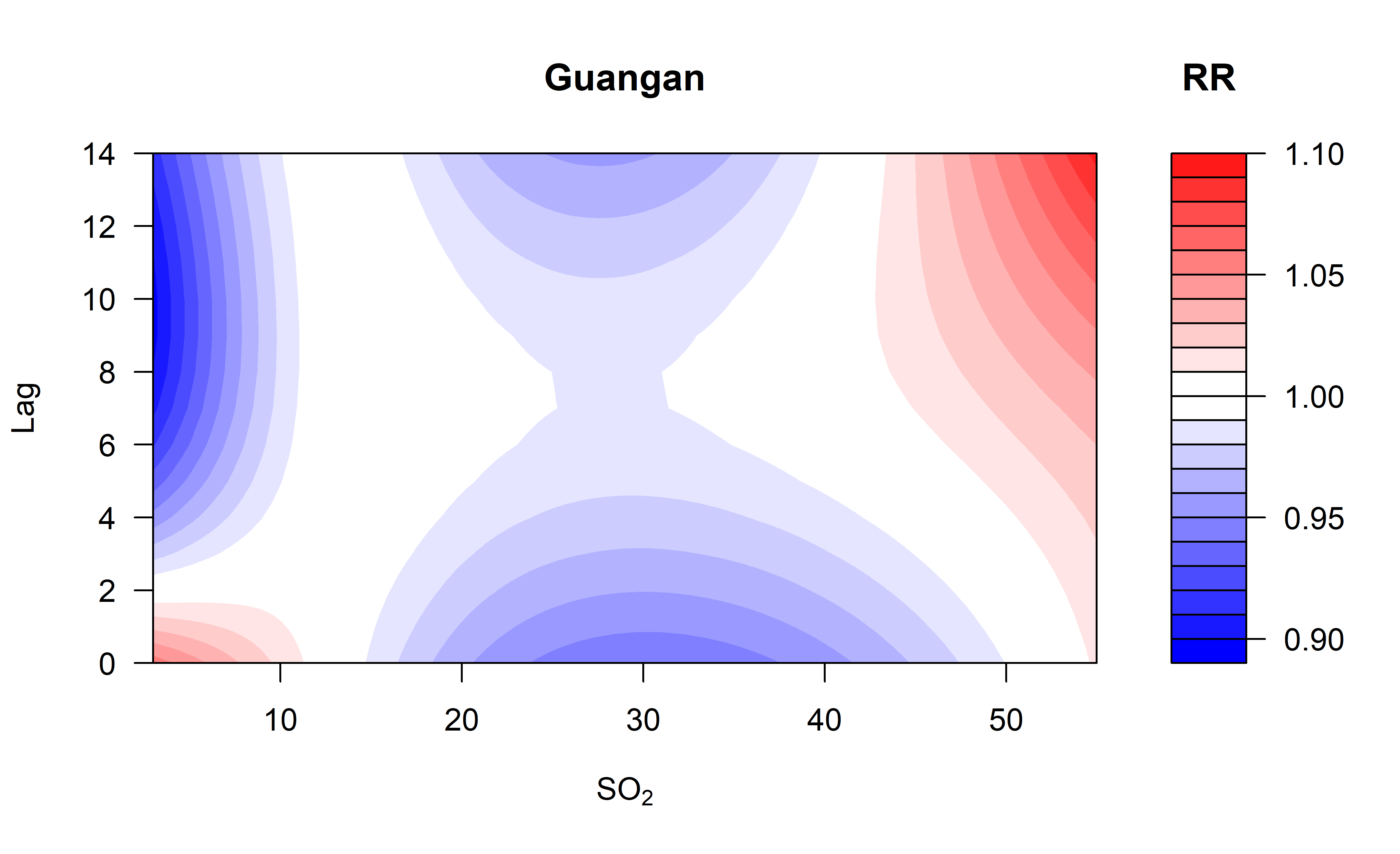

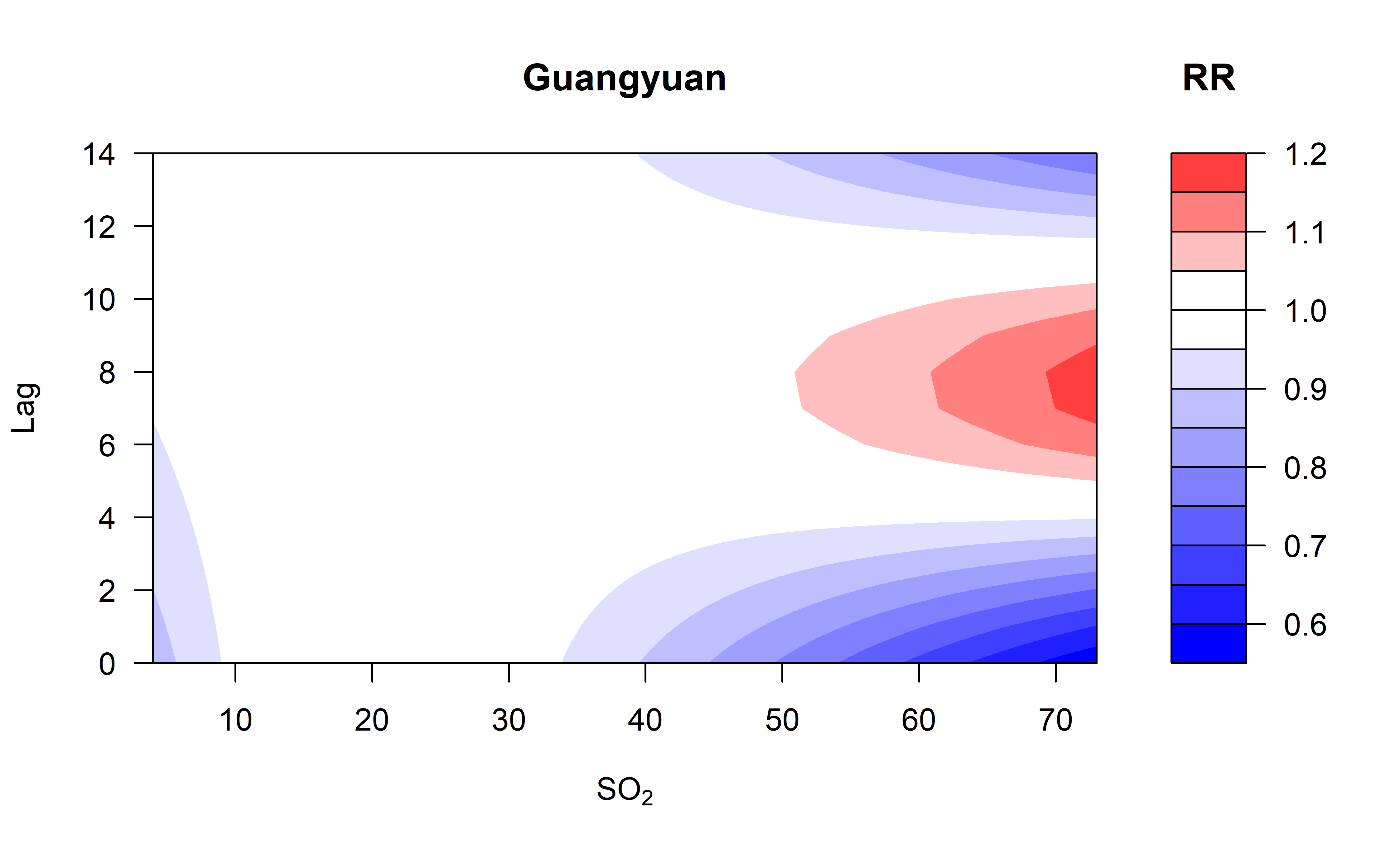

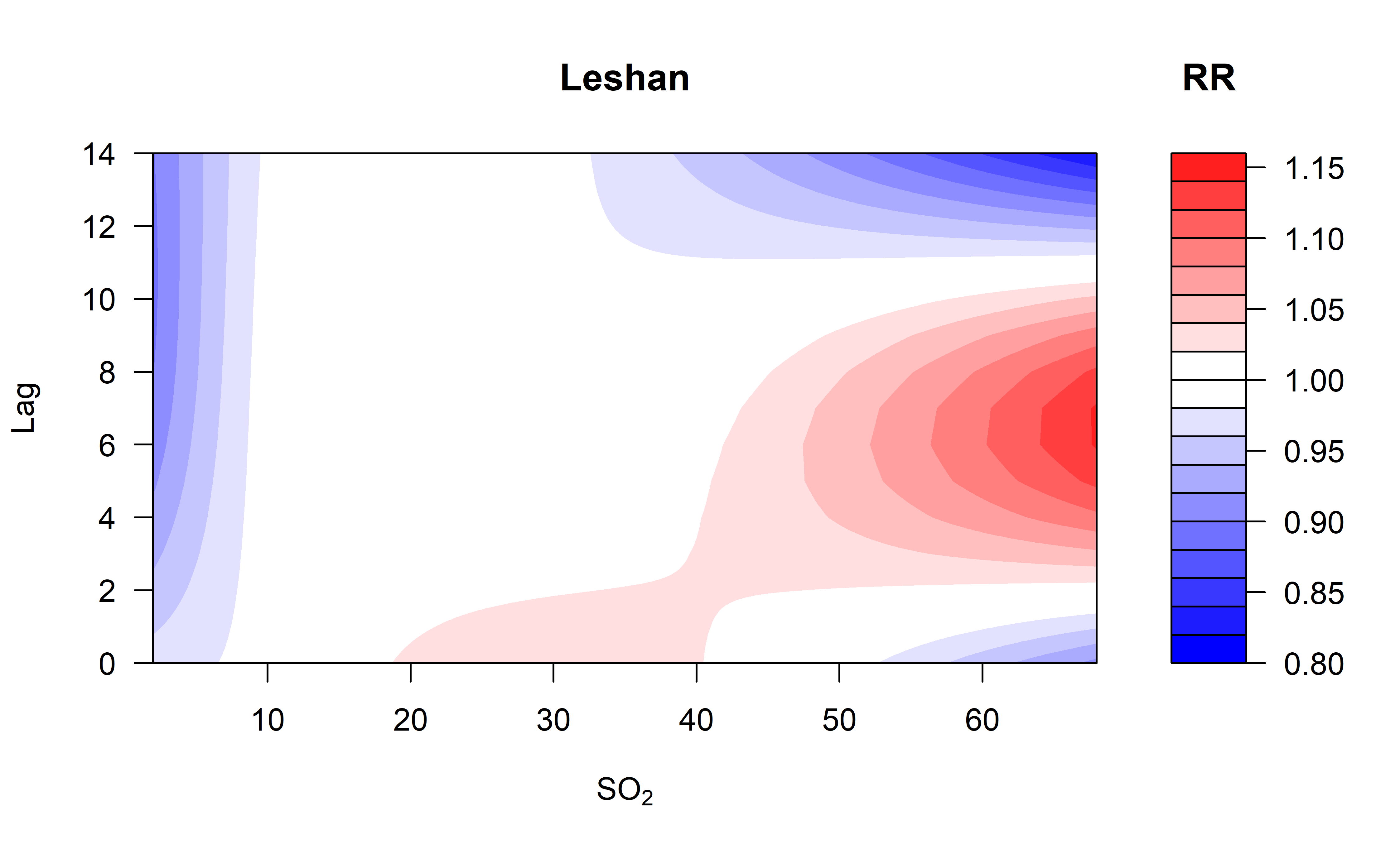

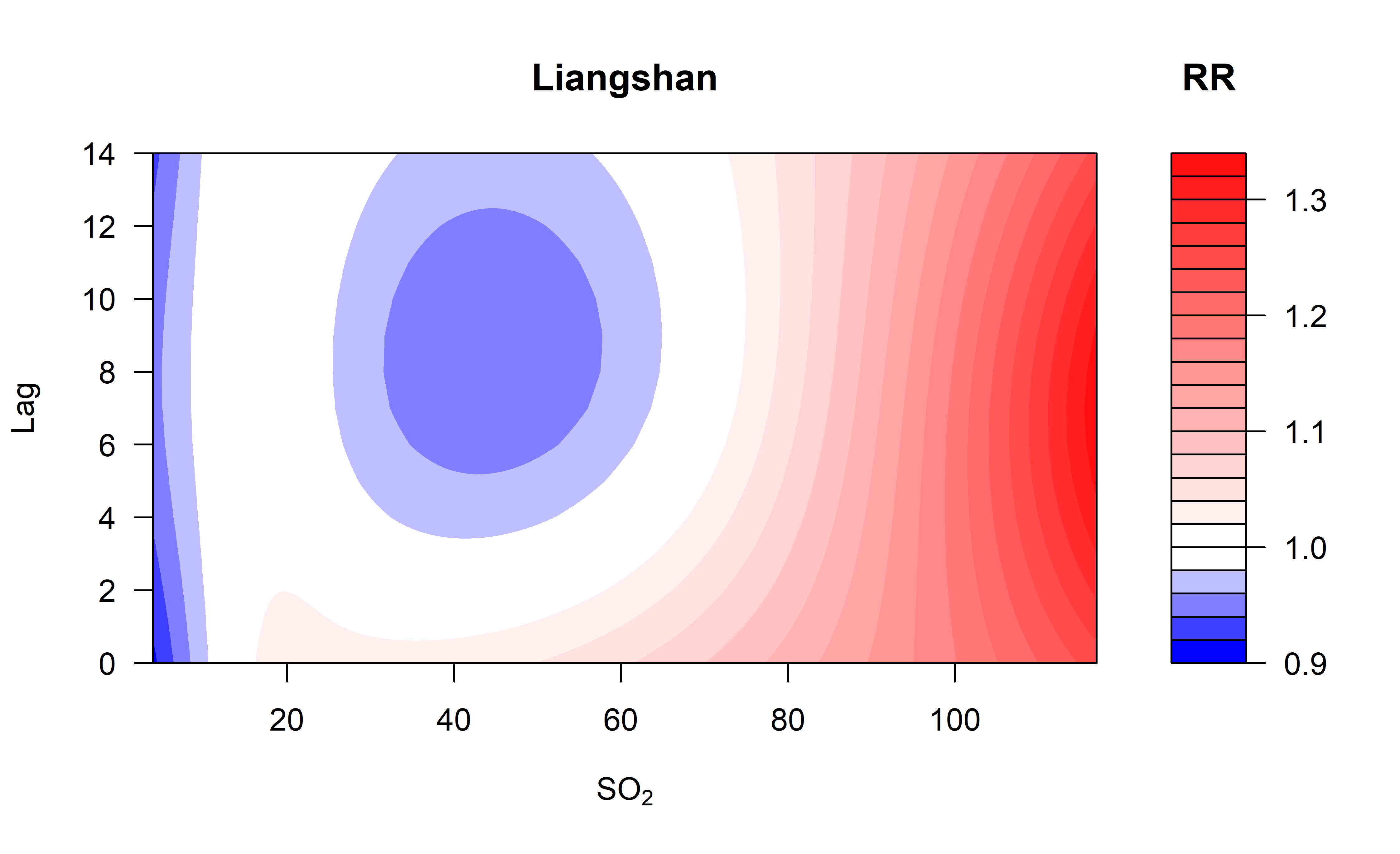

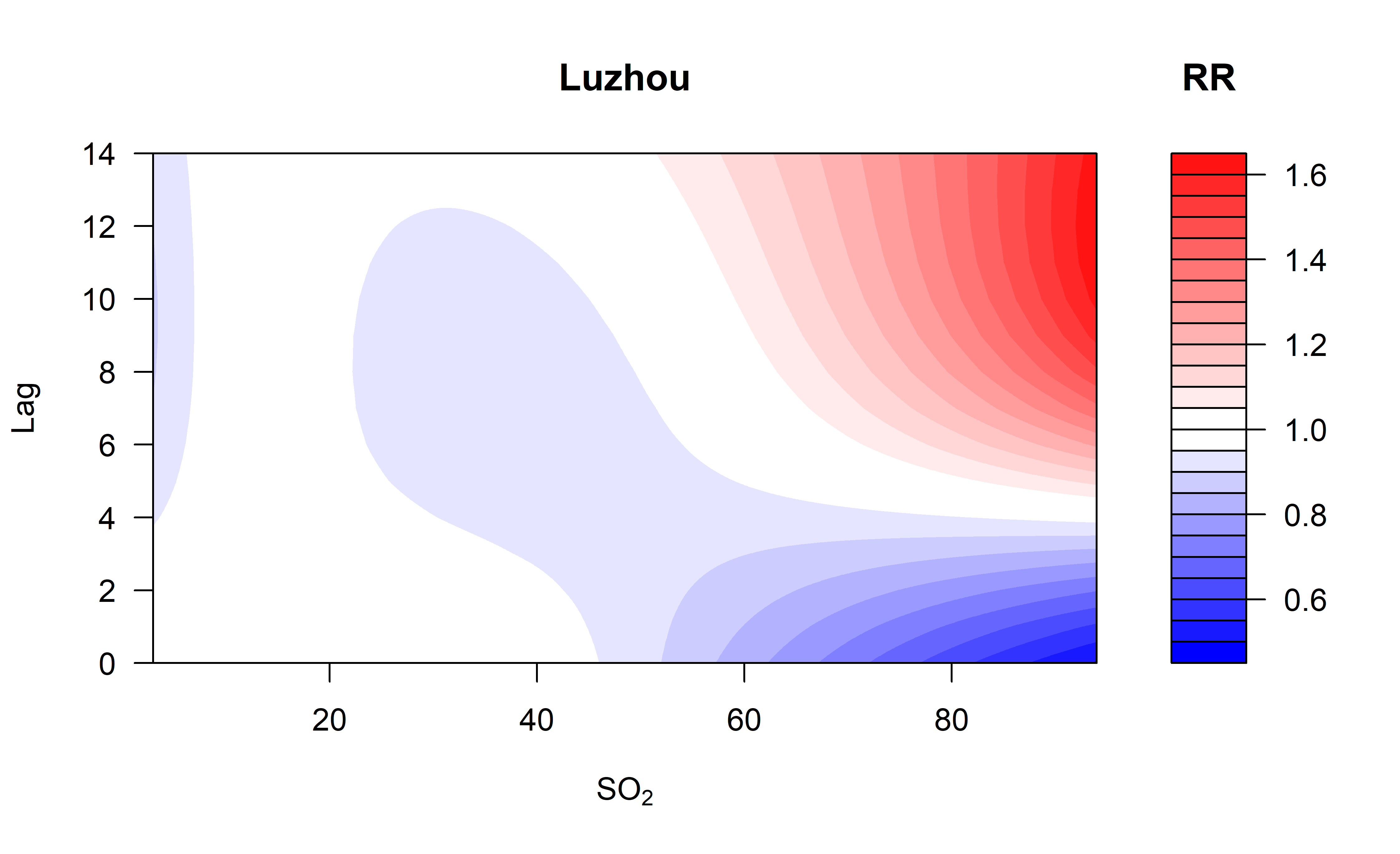

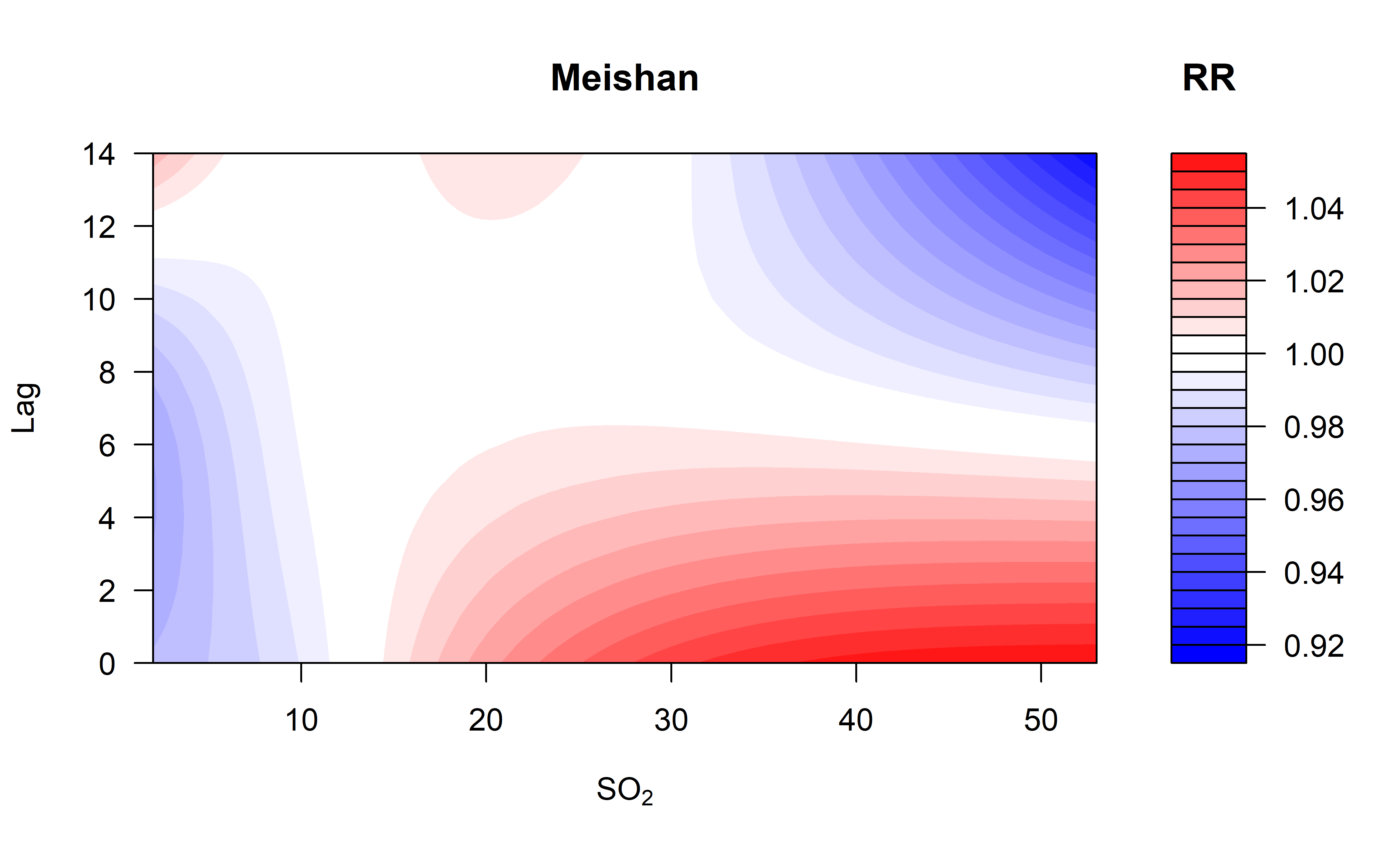

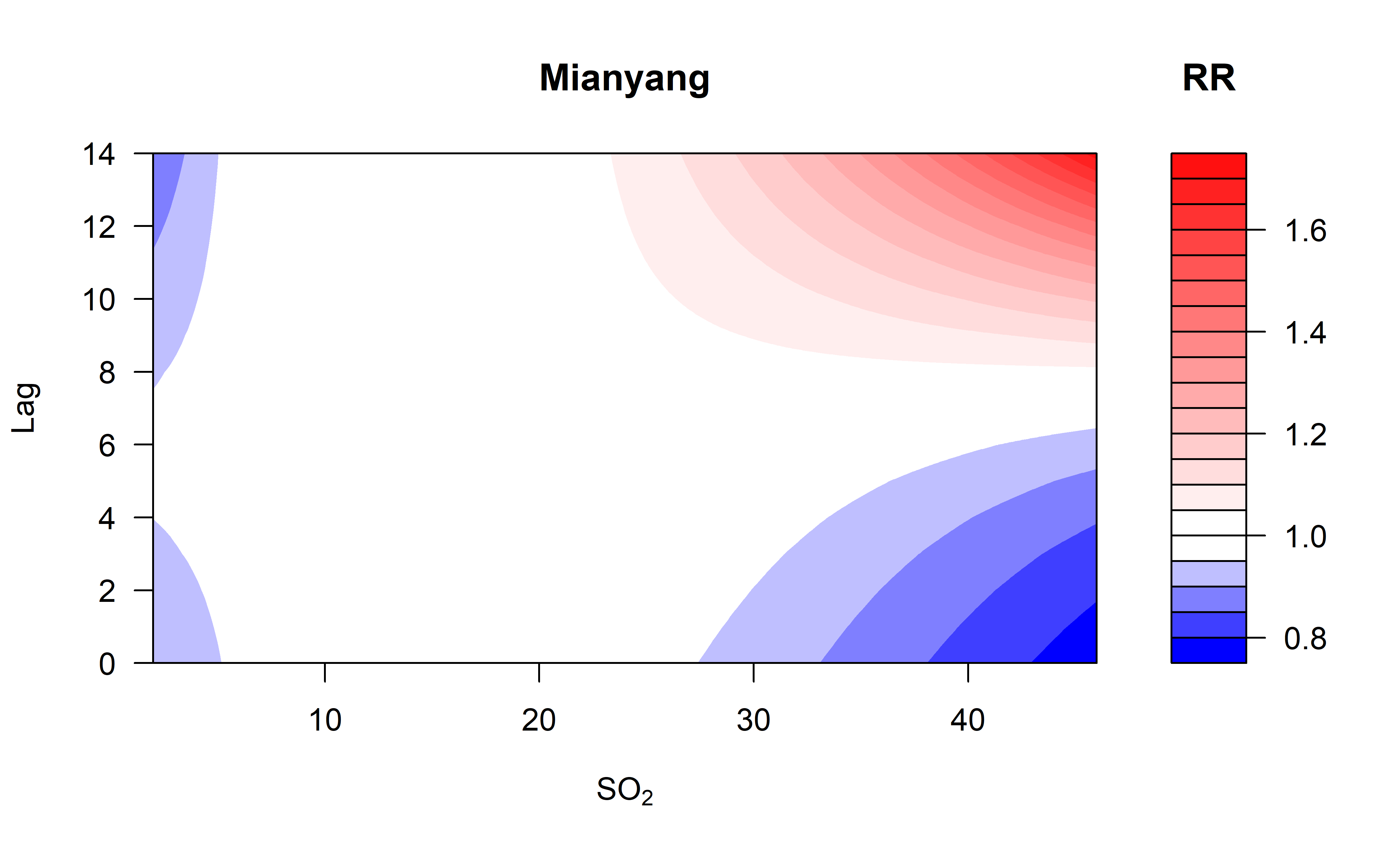

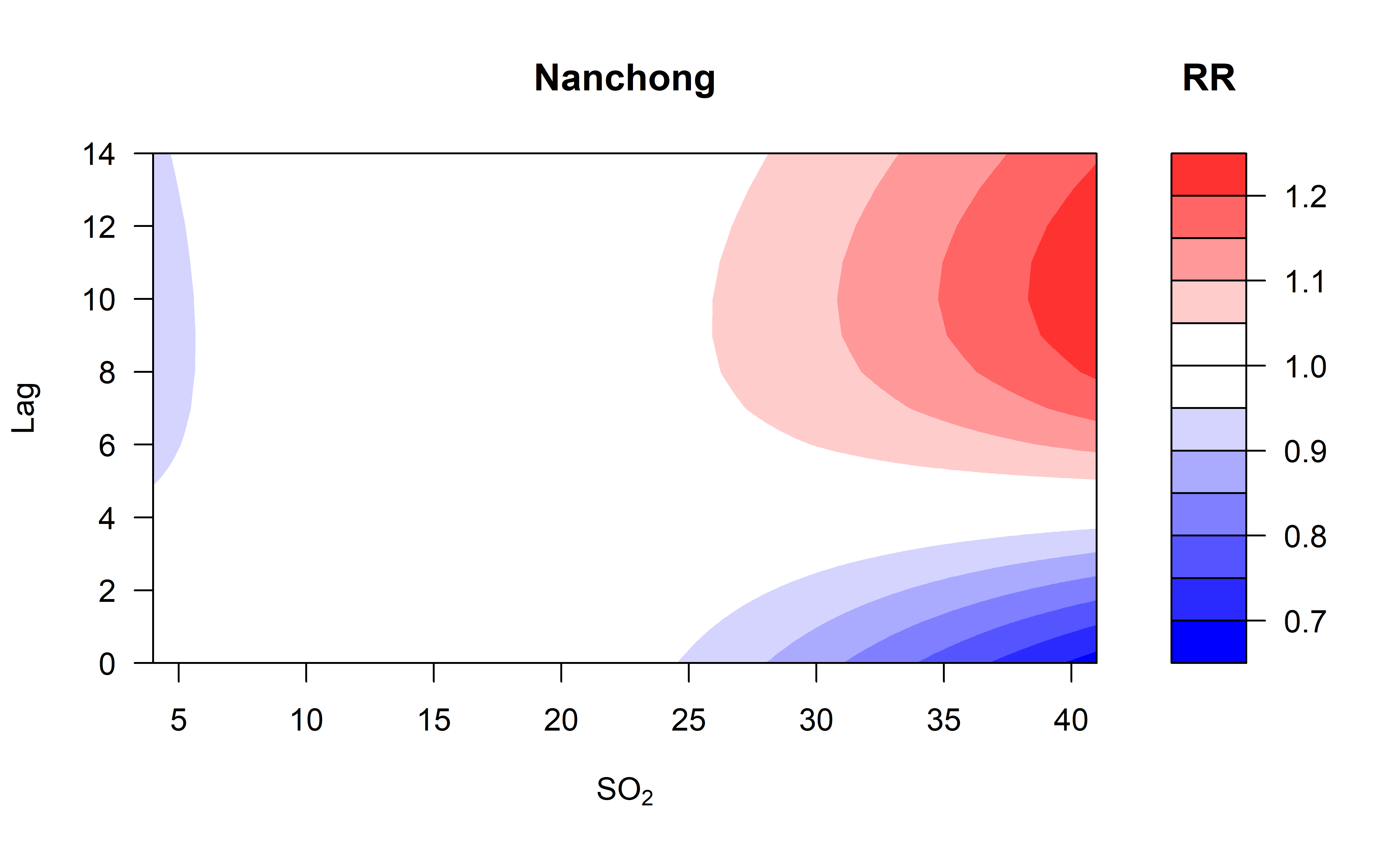

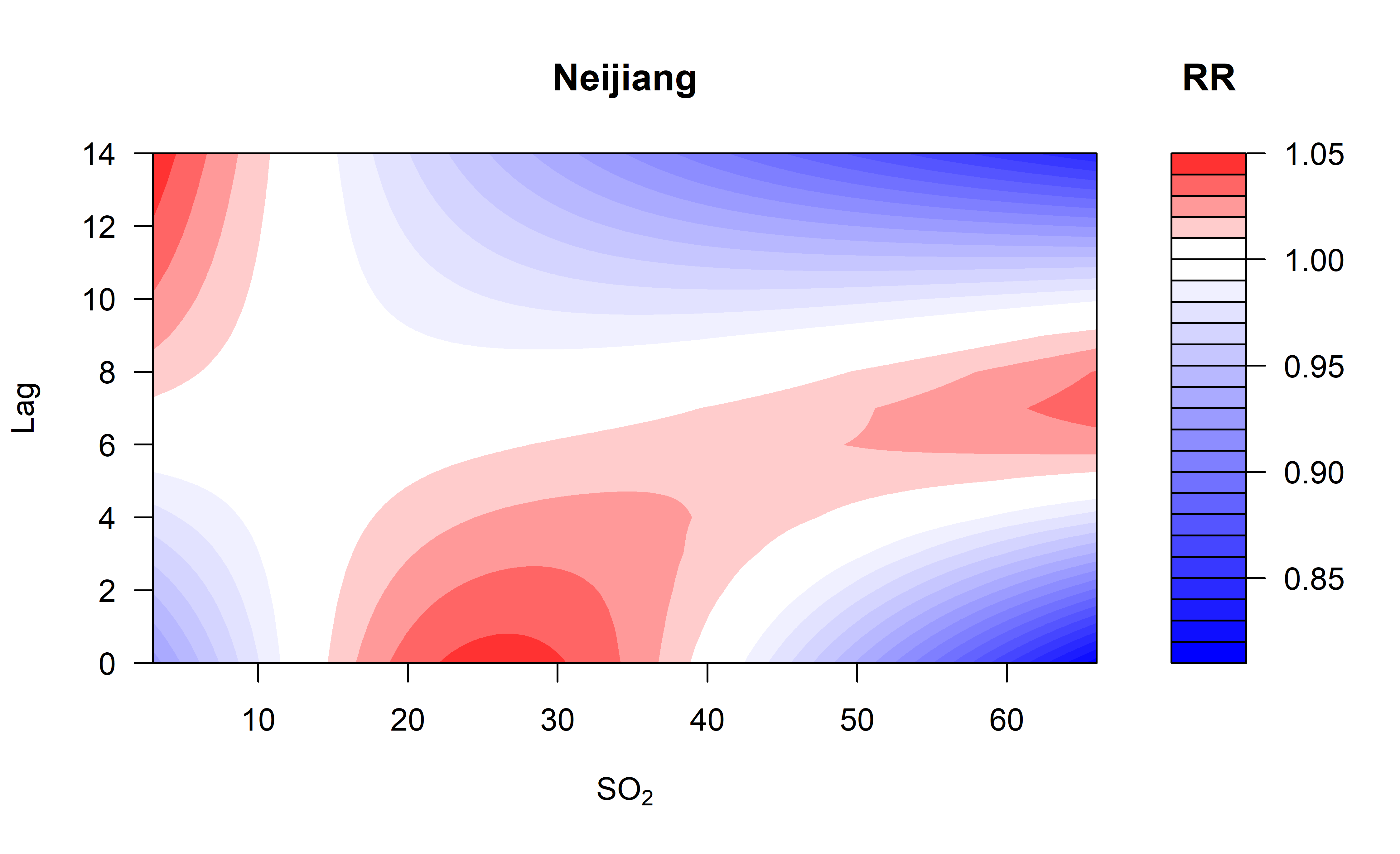

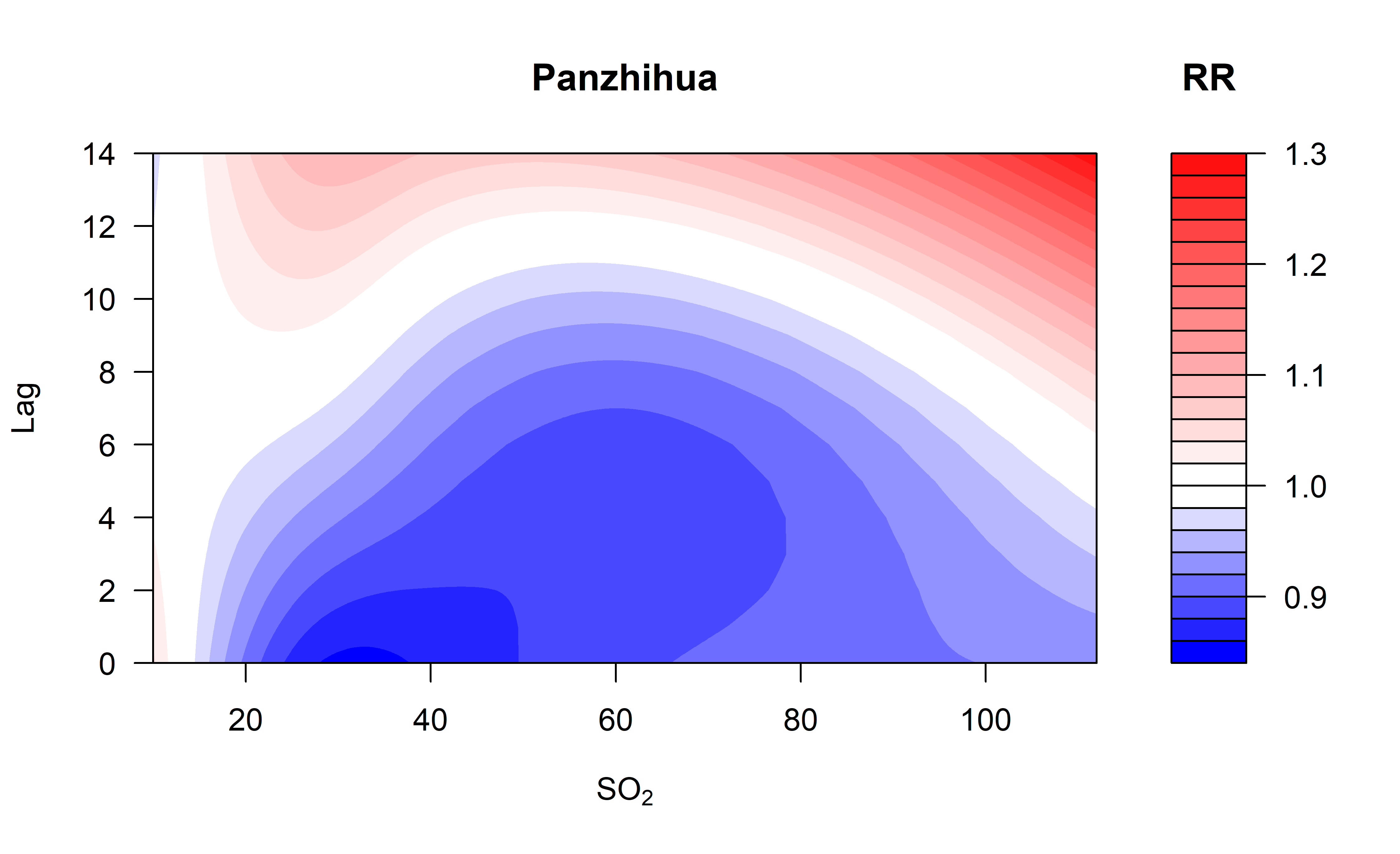

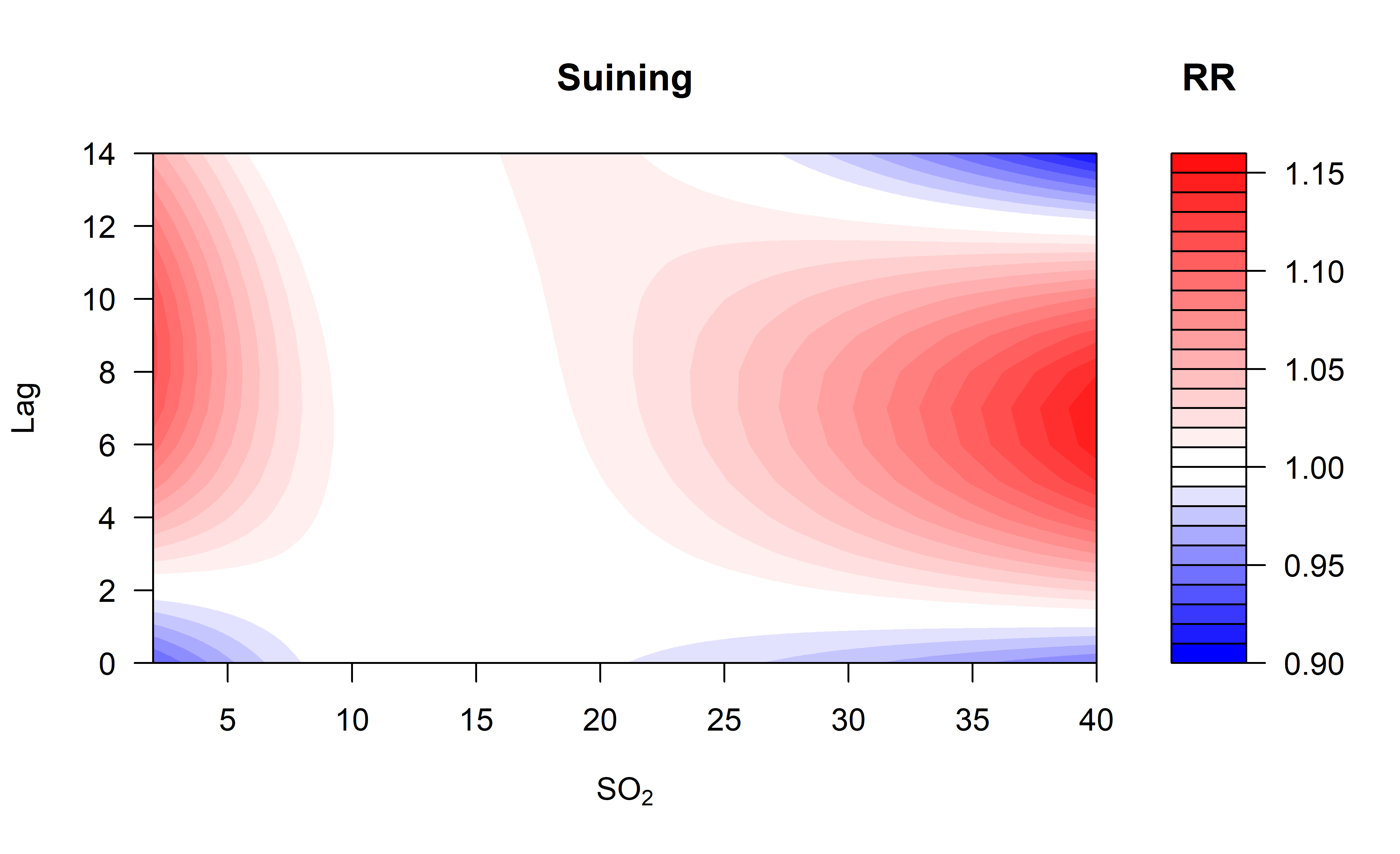

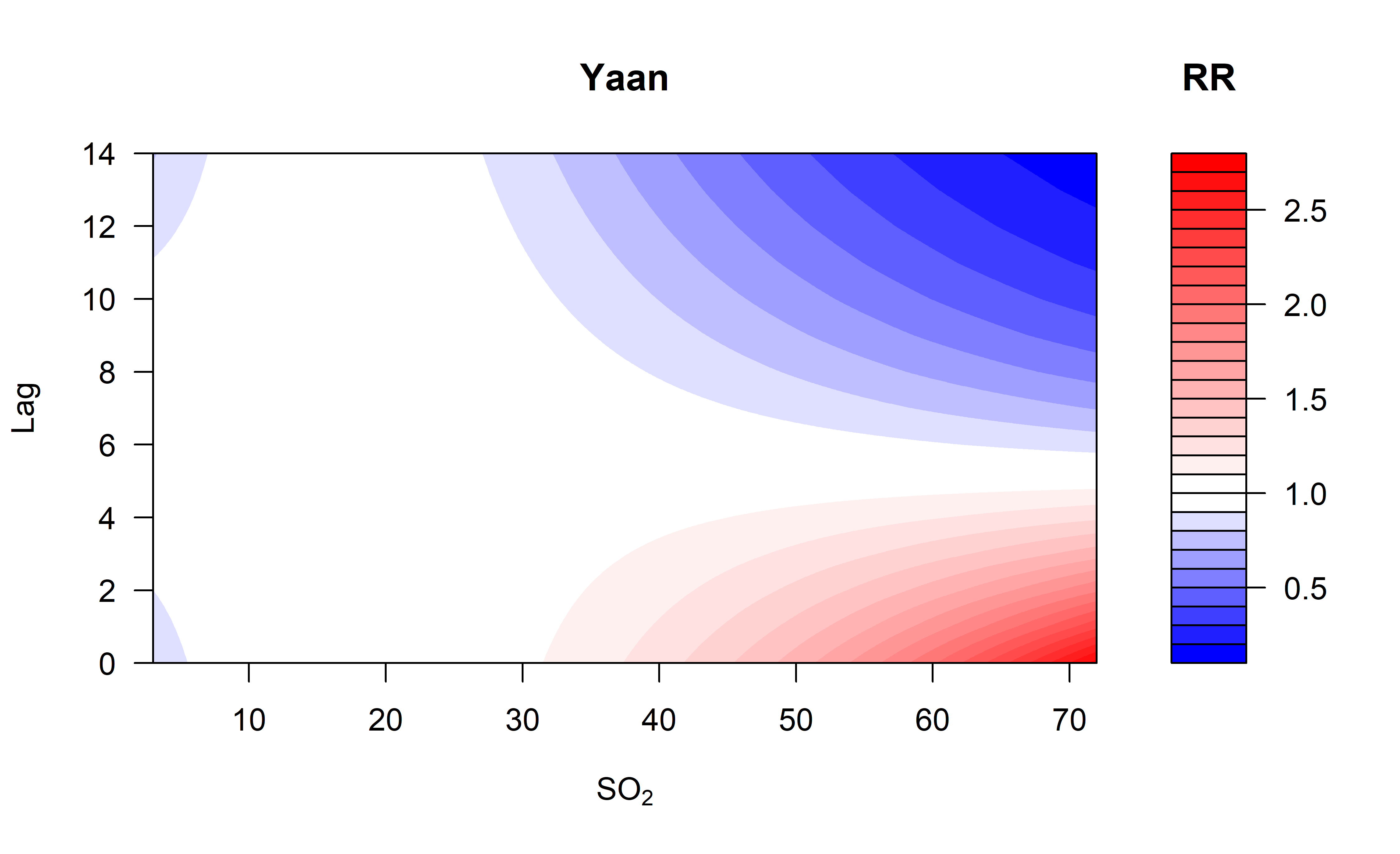

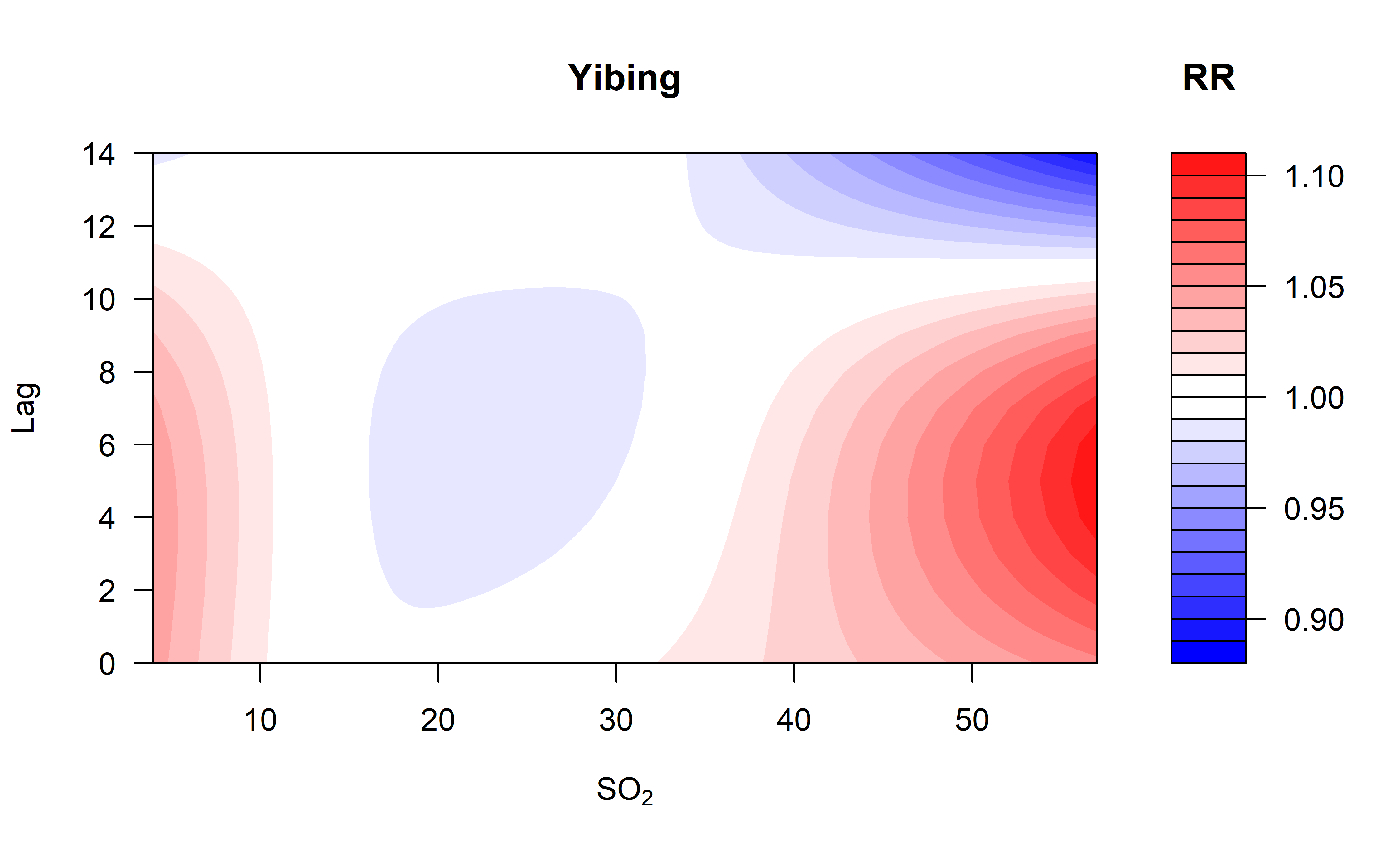

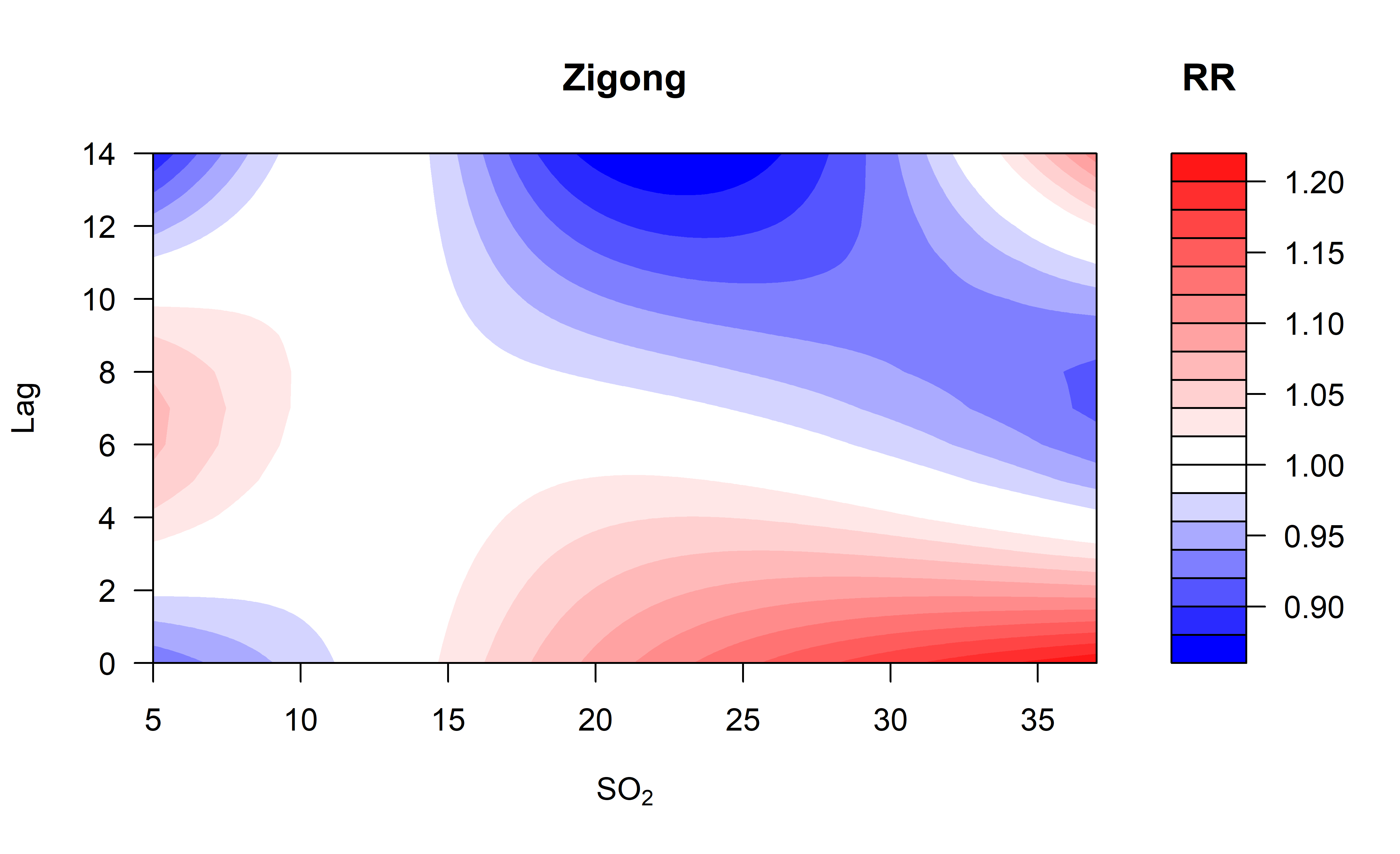

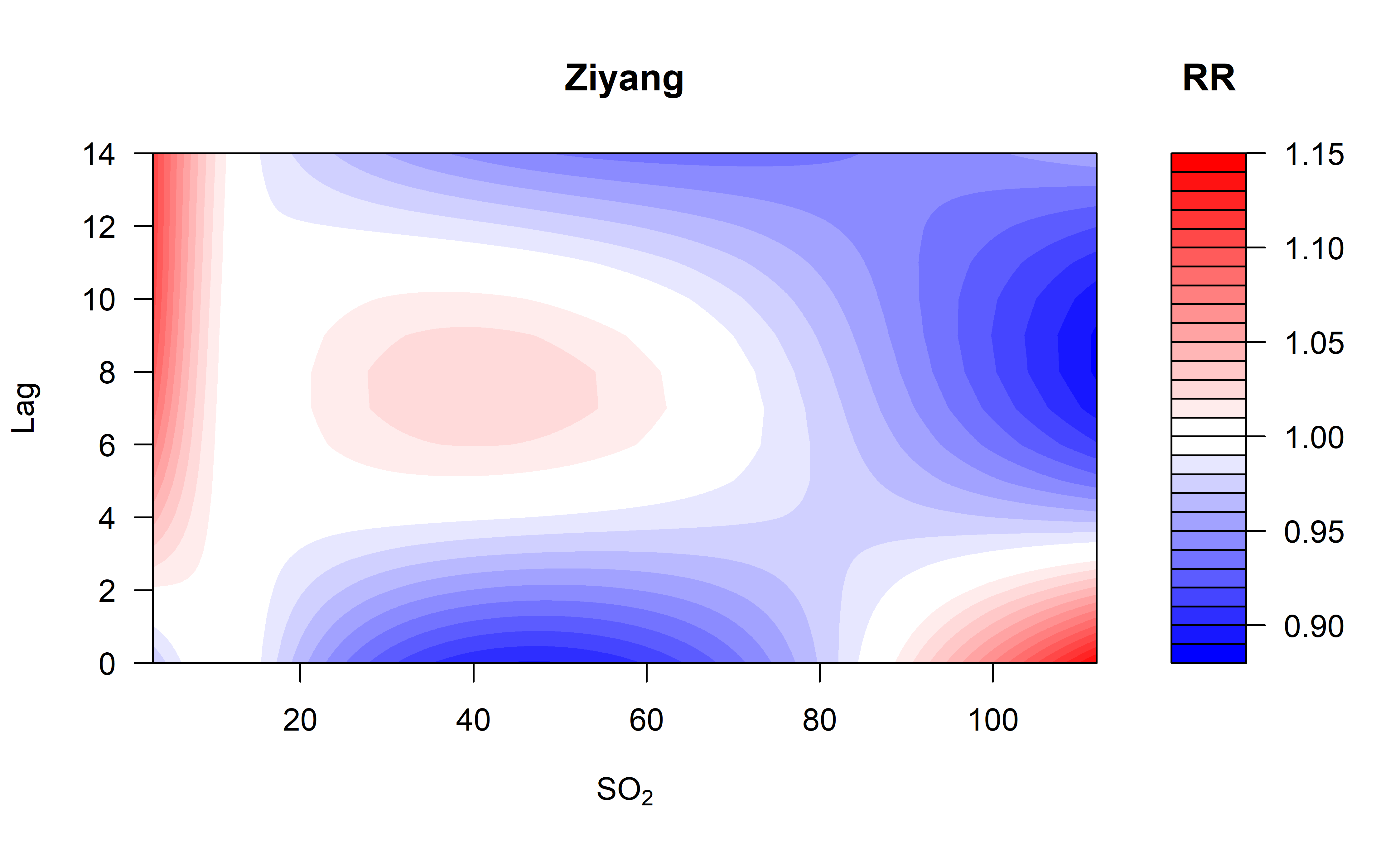


Fig. S3. Contour plots of the city-specific relationship between the risk of HFMD and SO_2_ at different time lags.


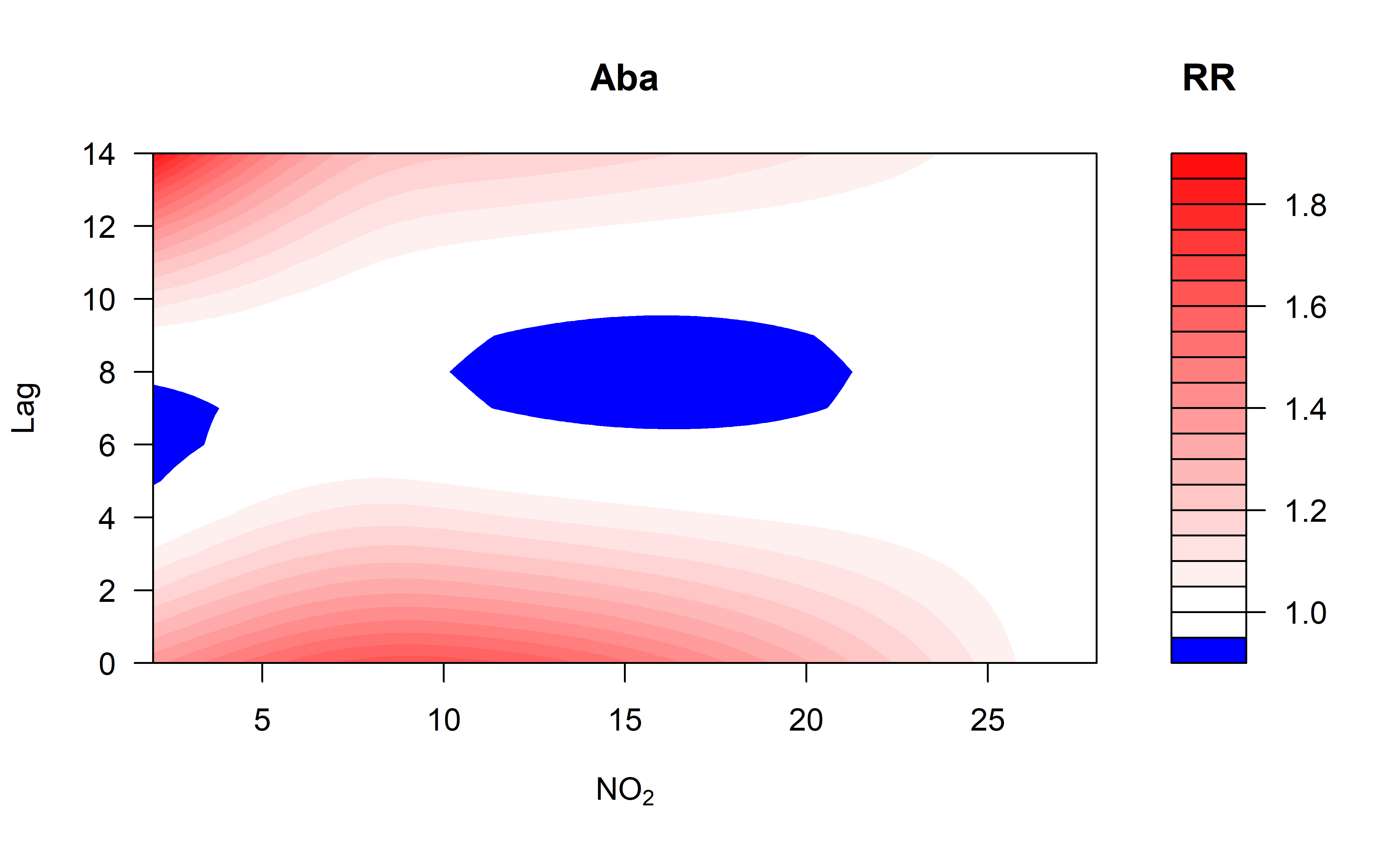

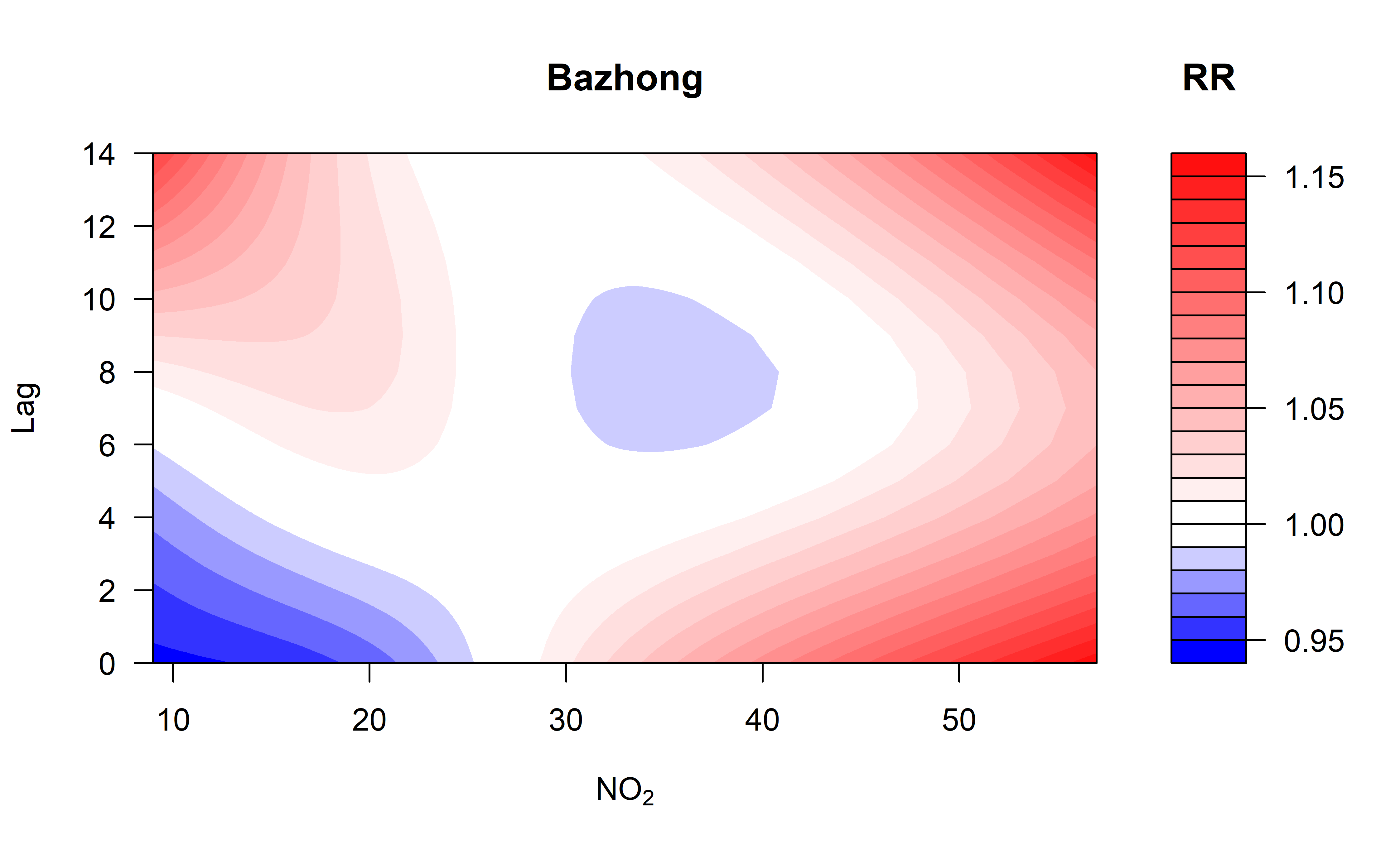

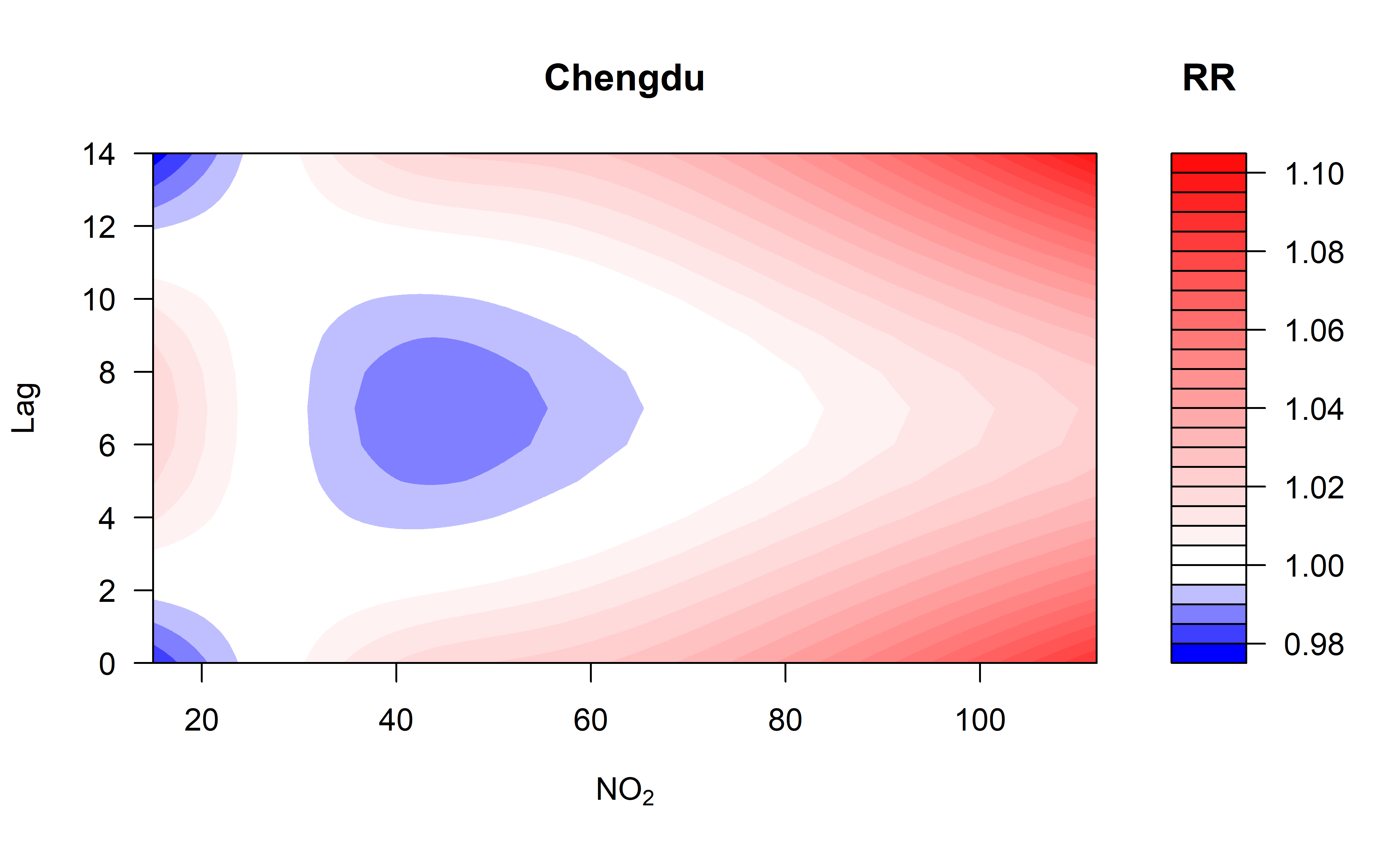

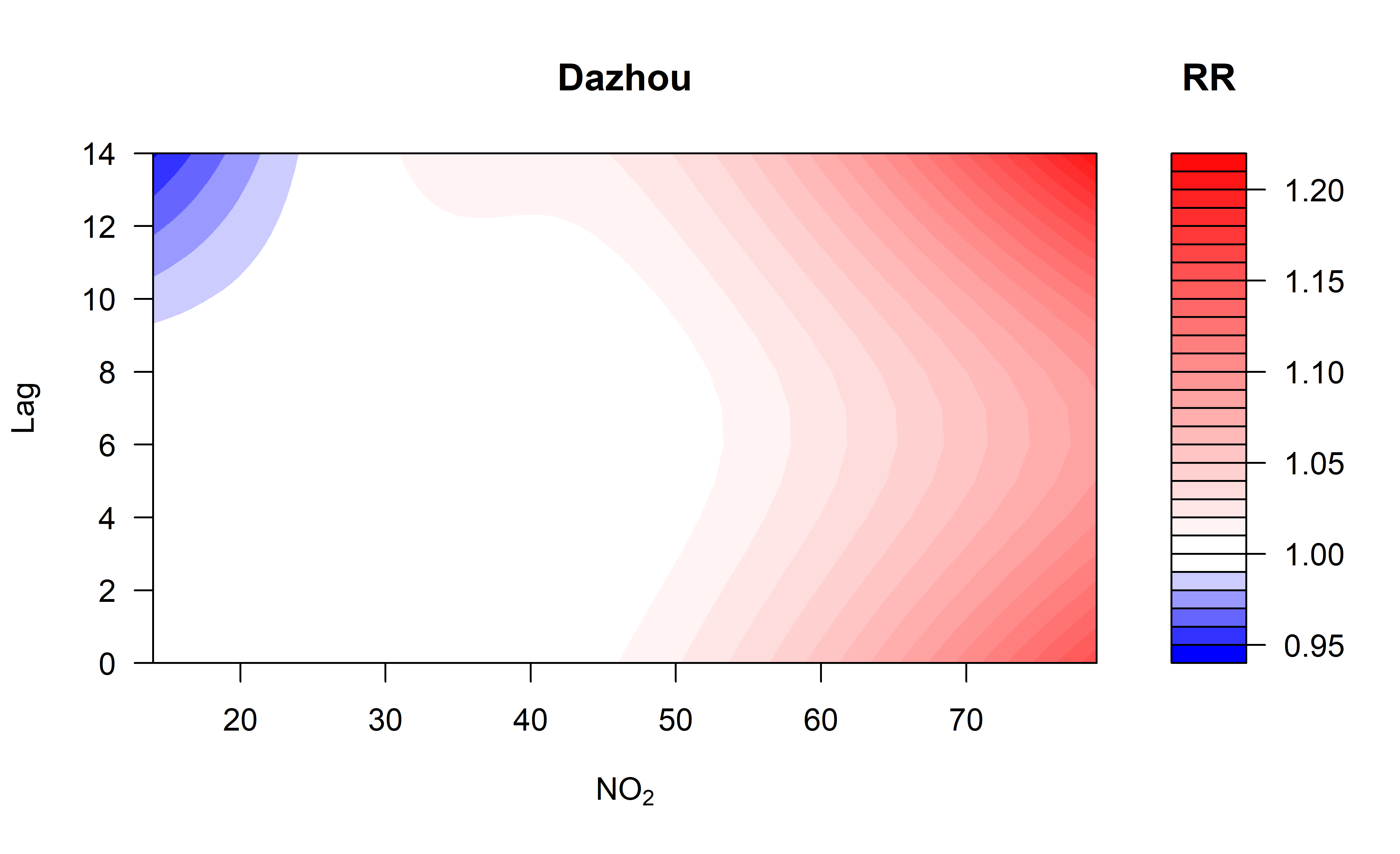

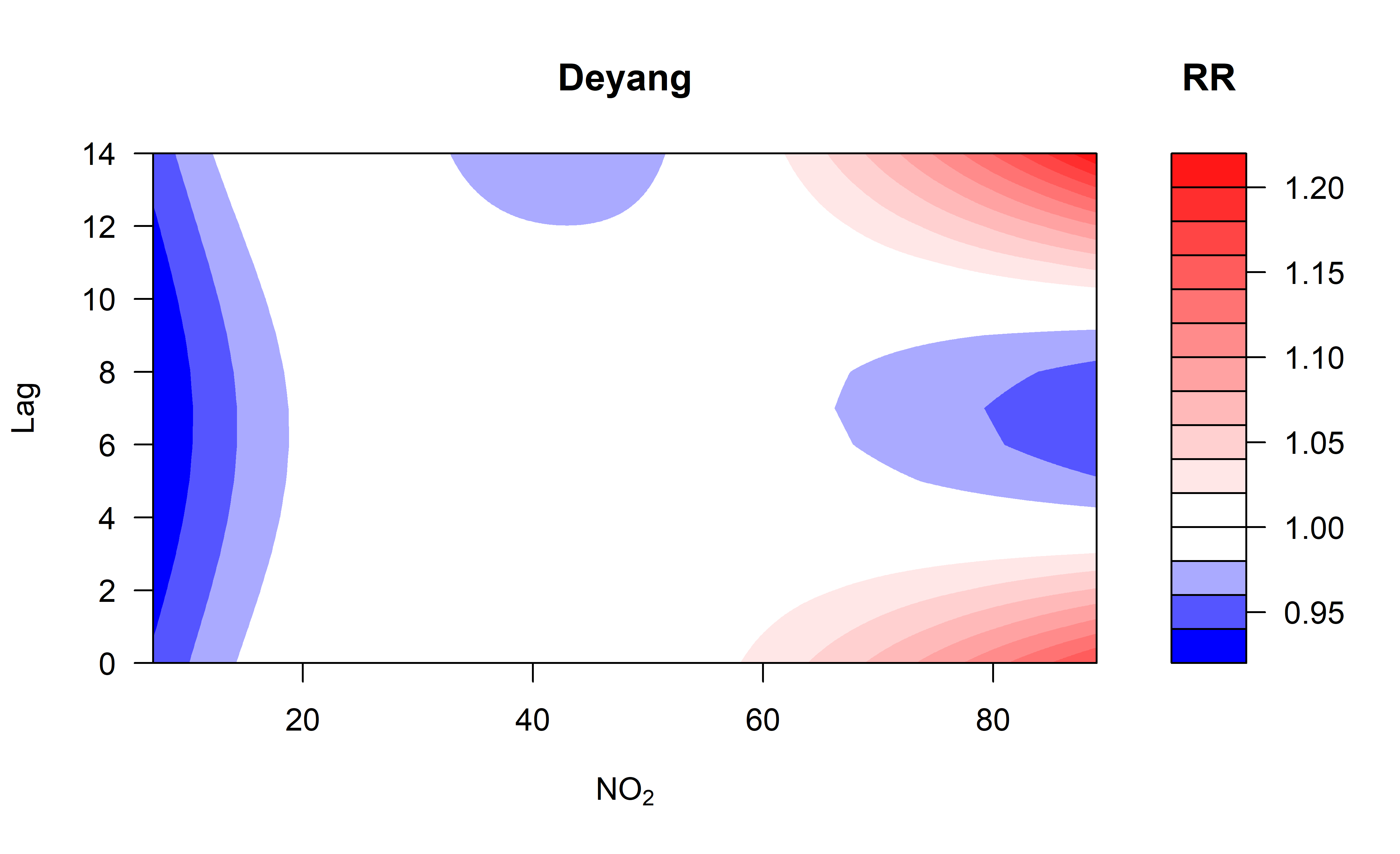

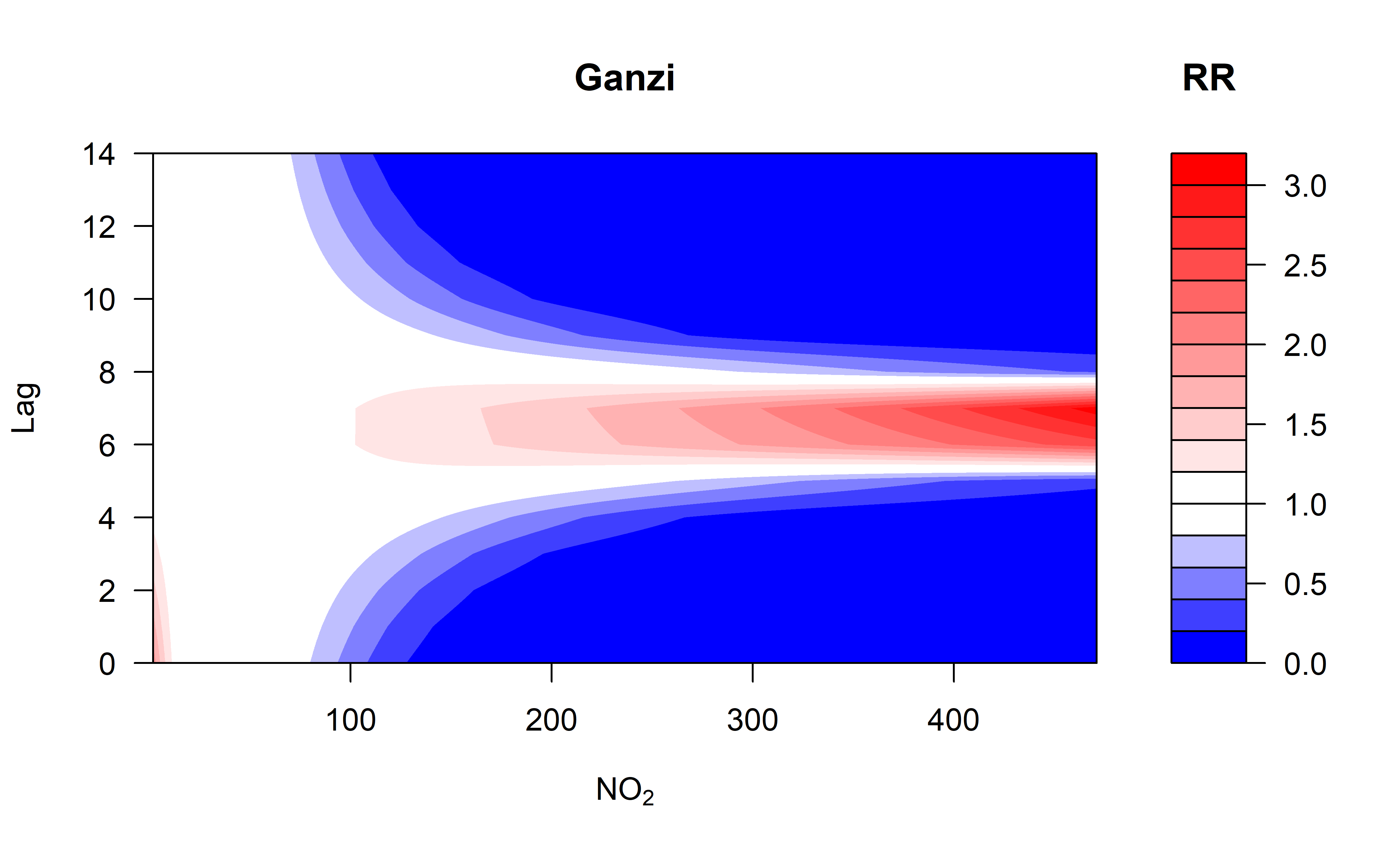

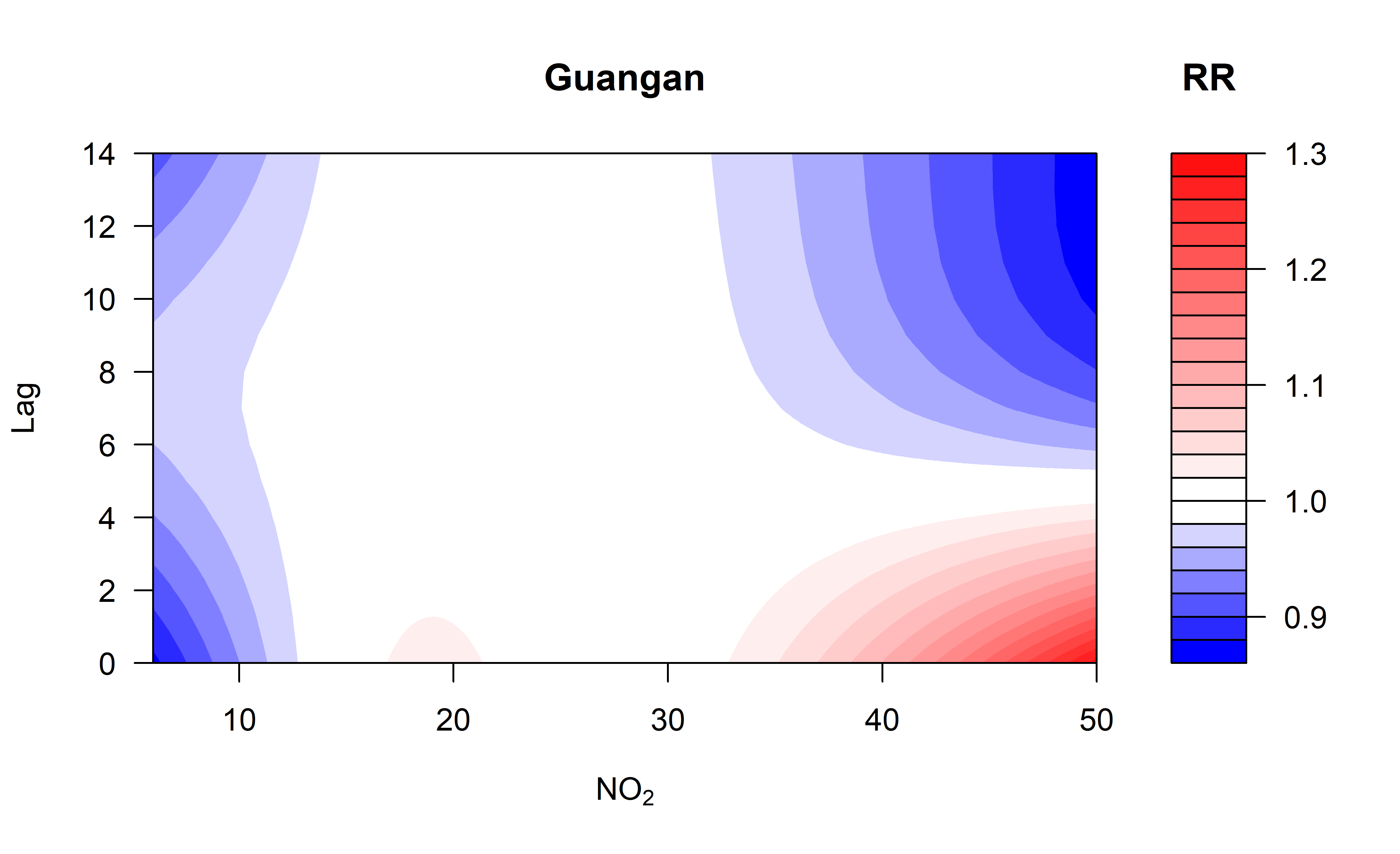

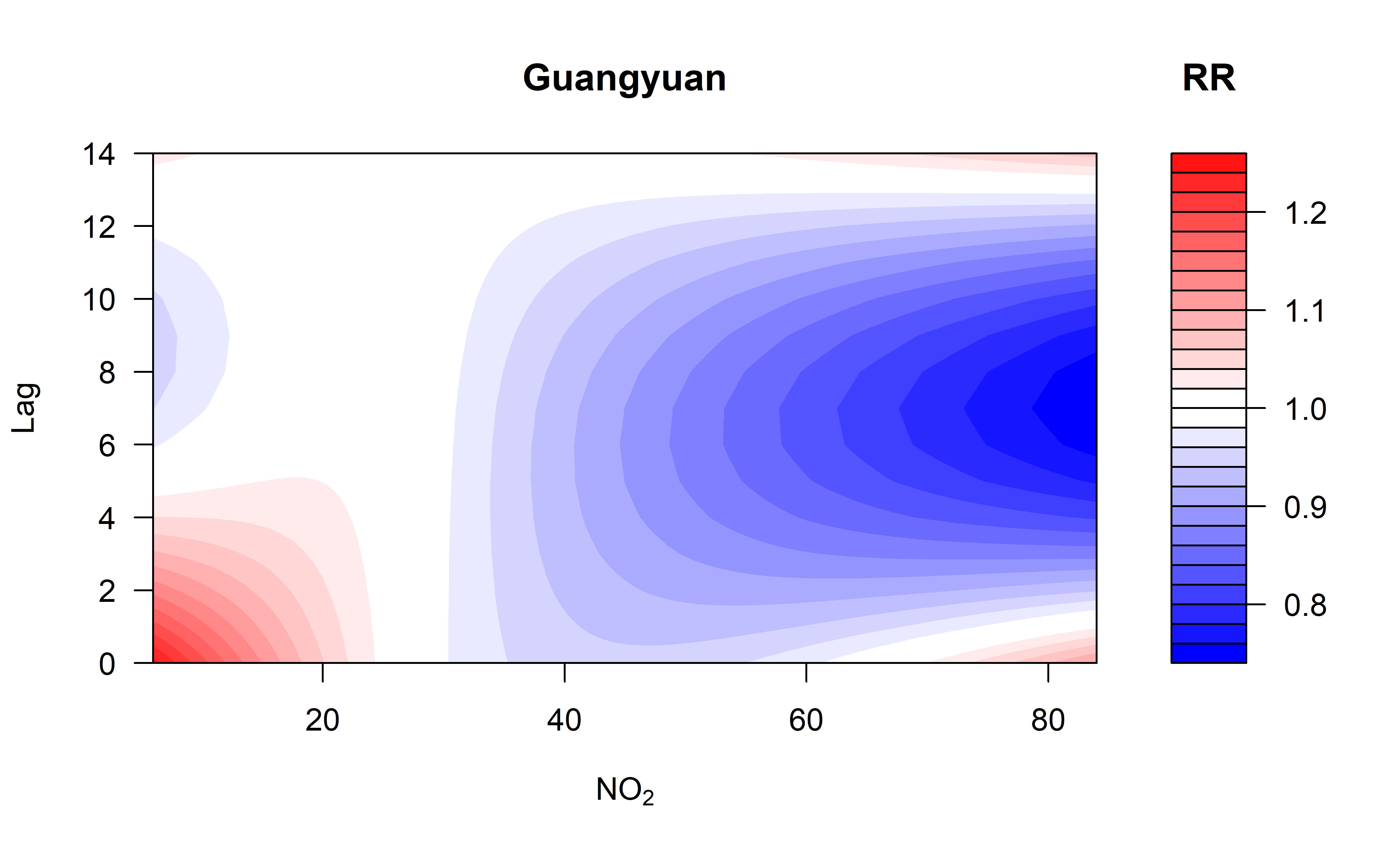

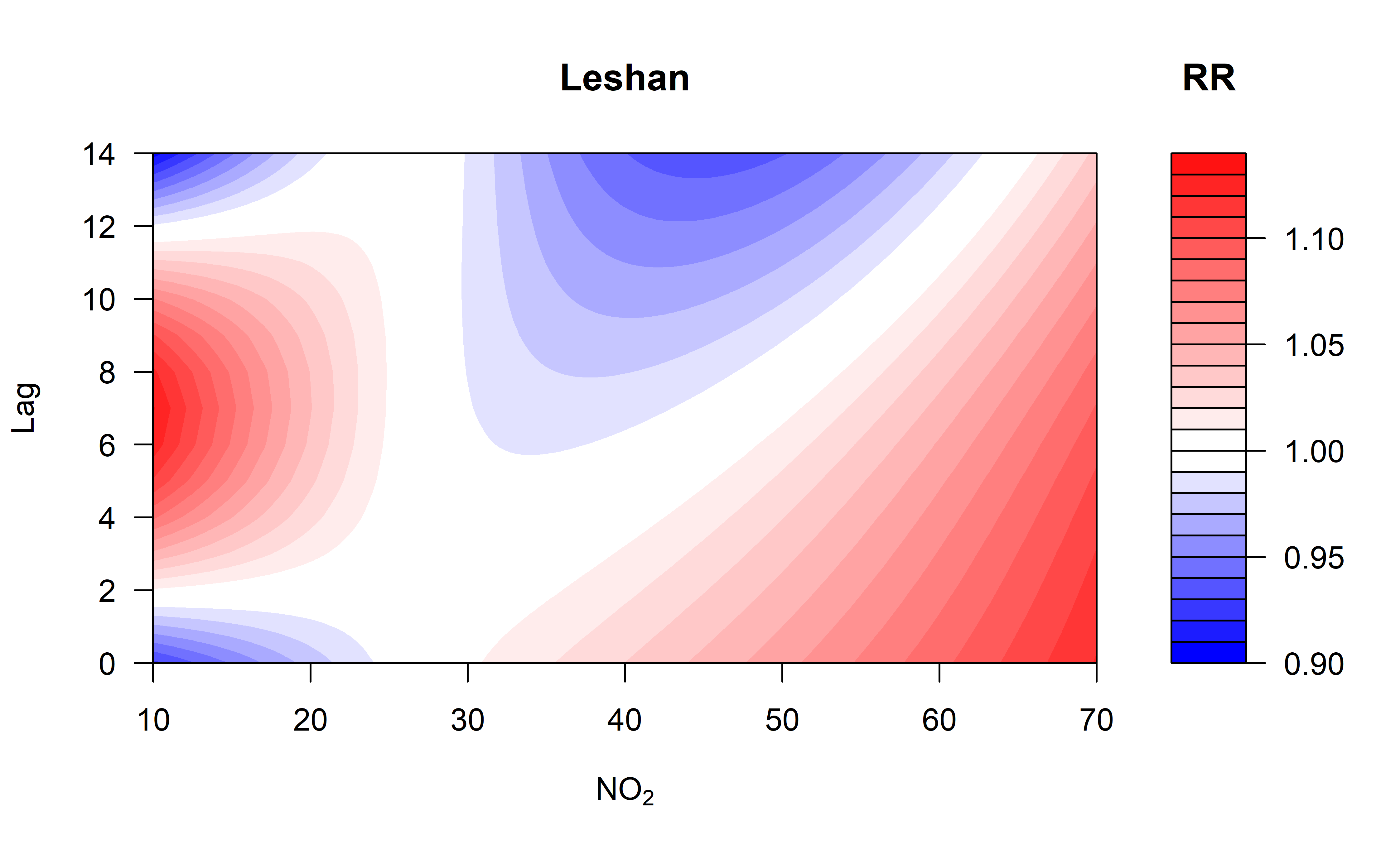

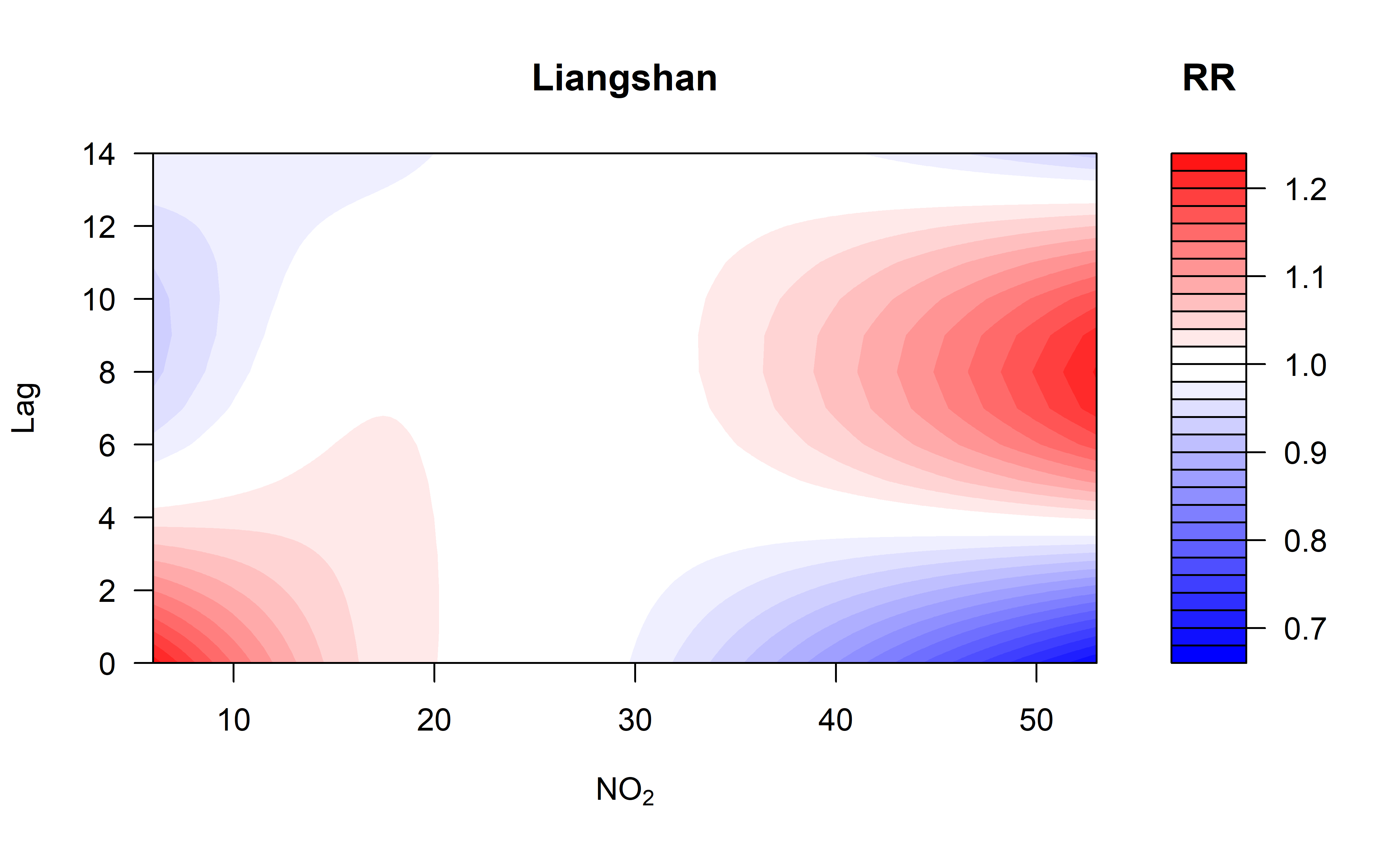

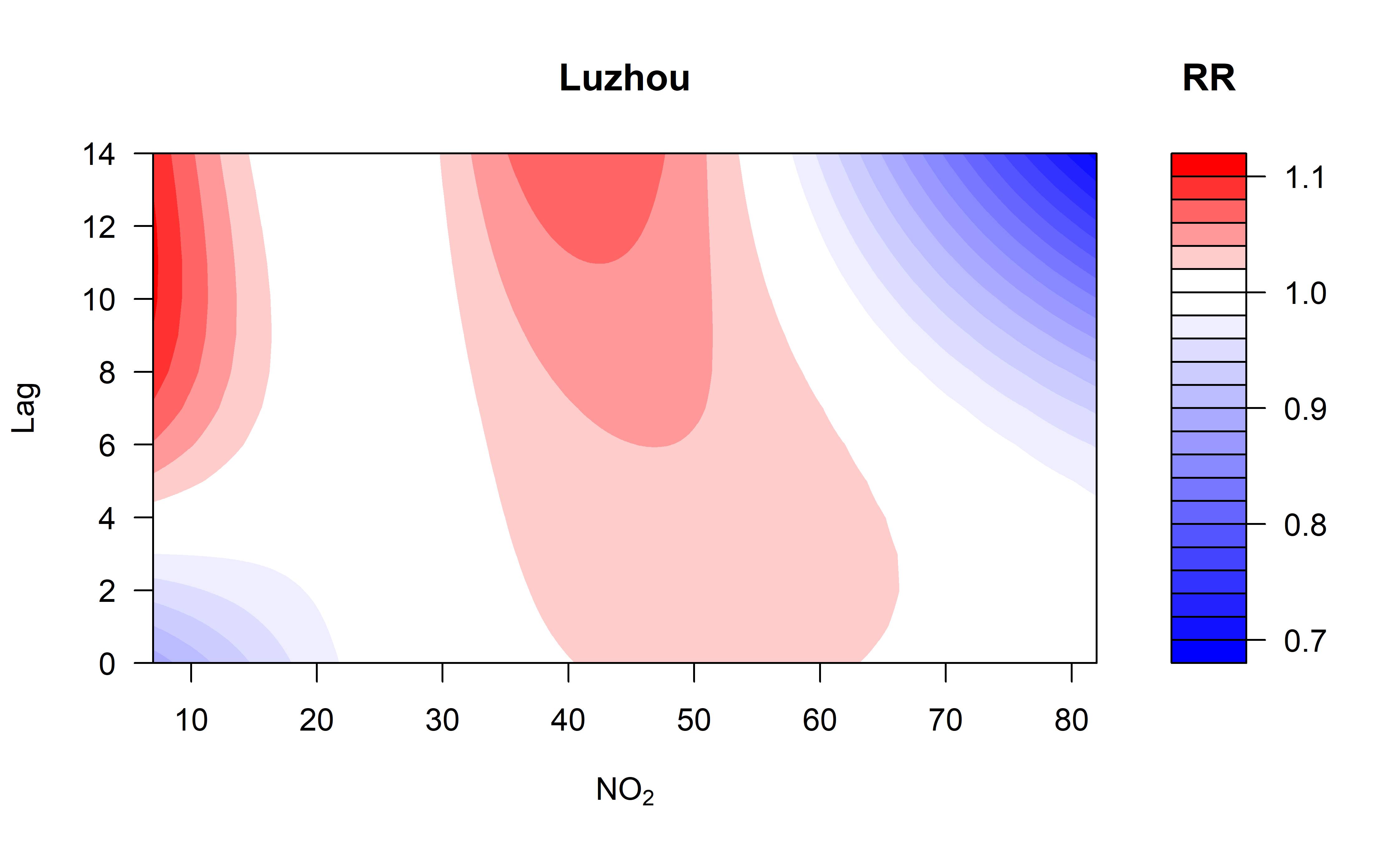

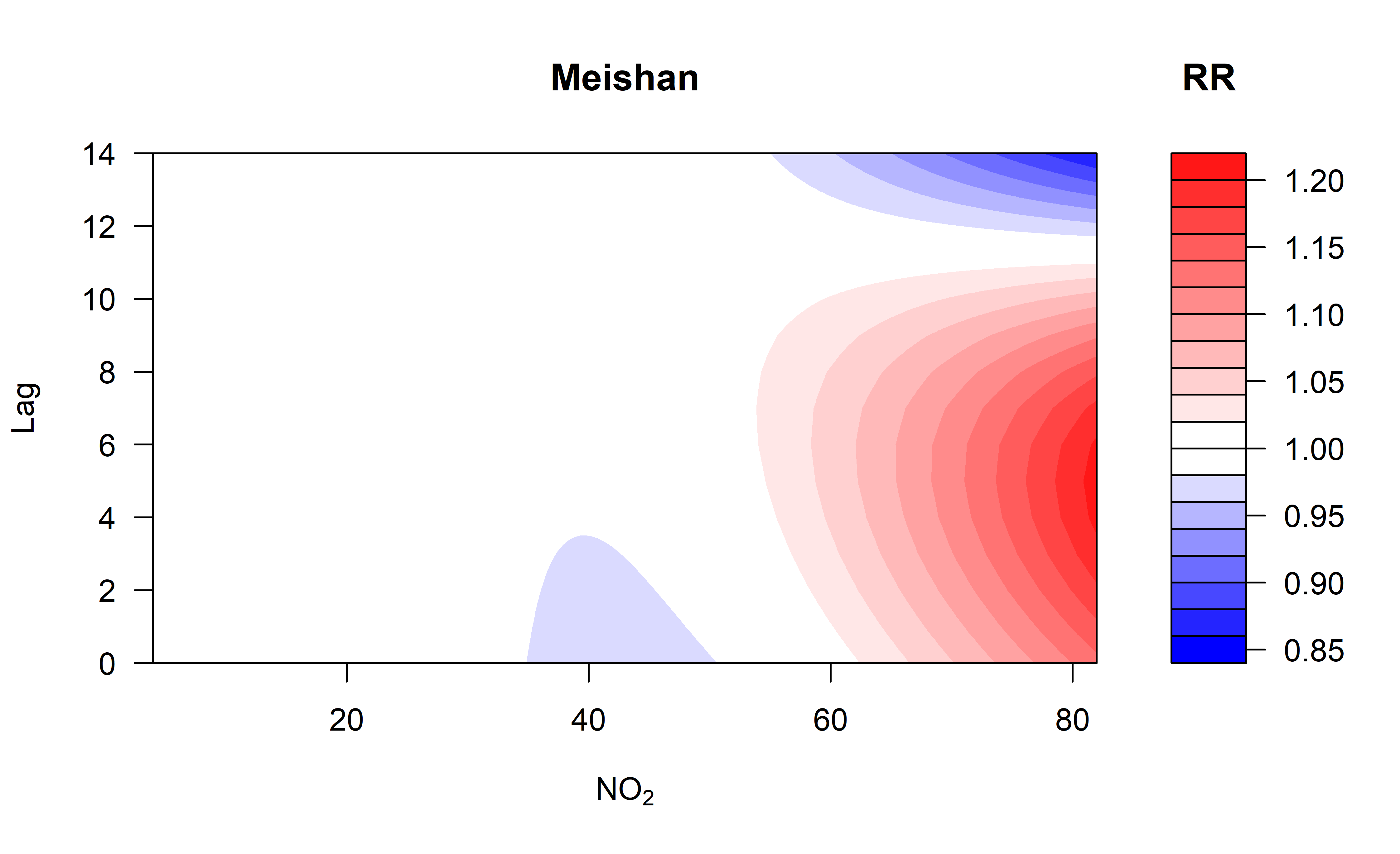

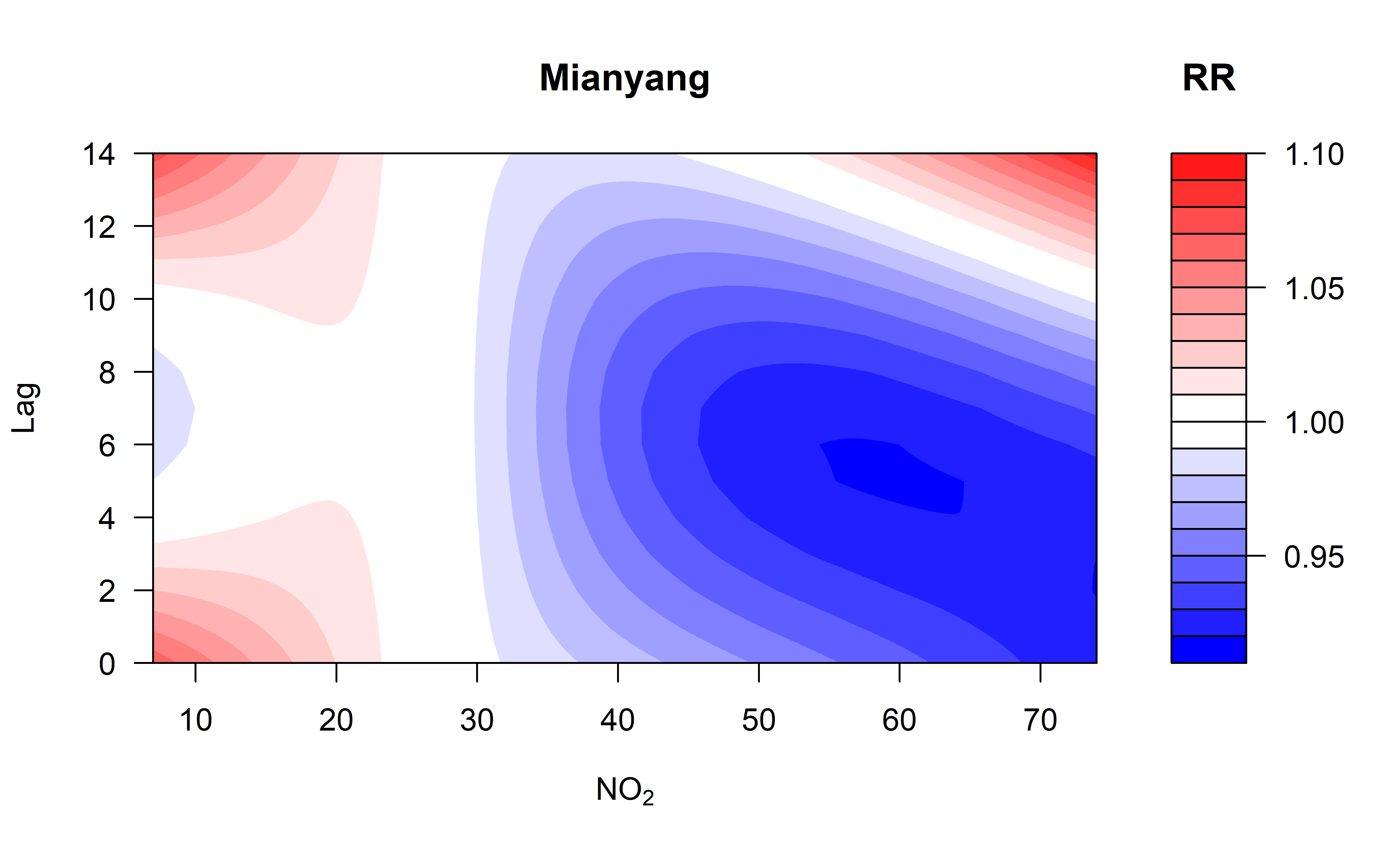

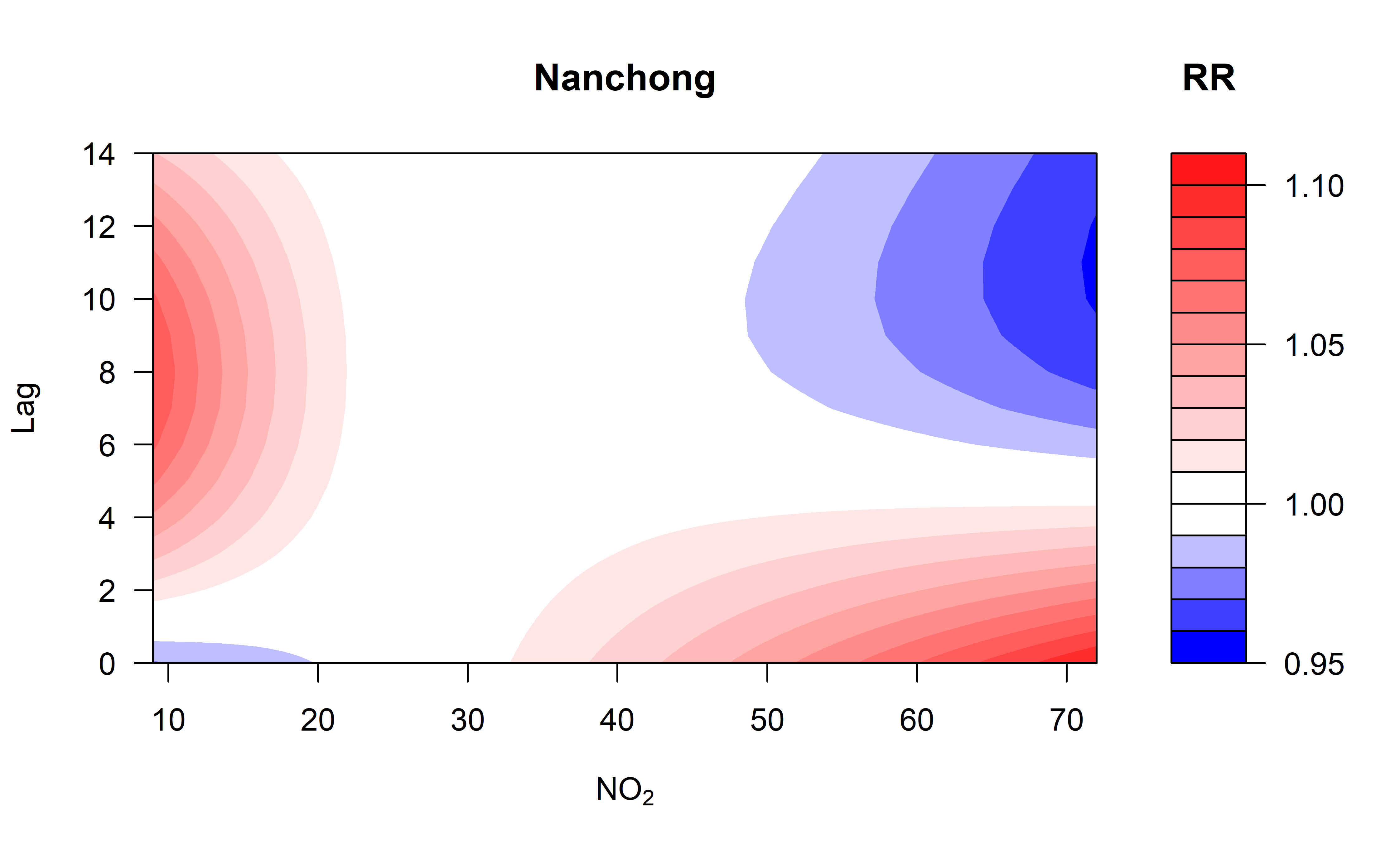

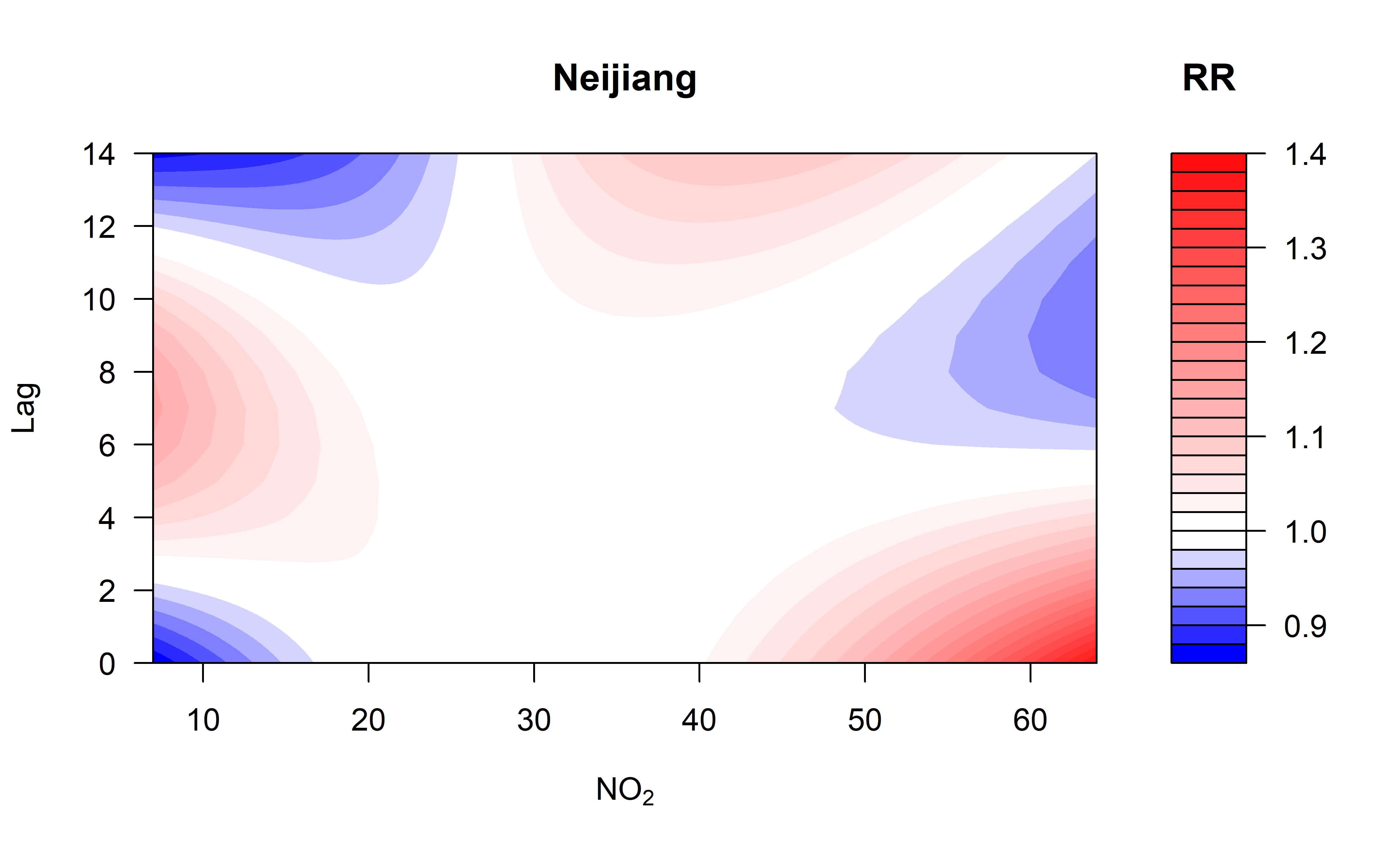

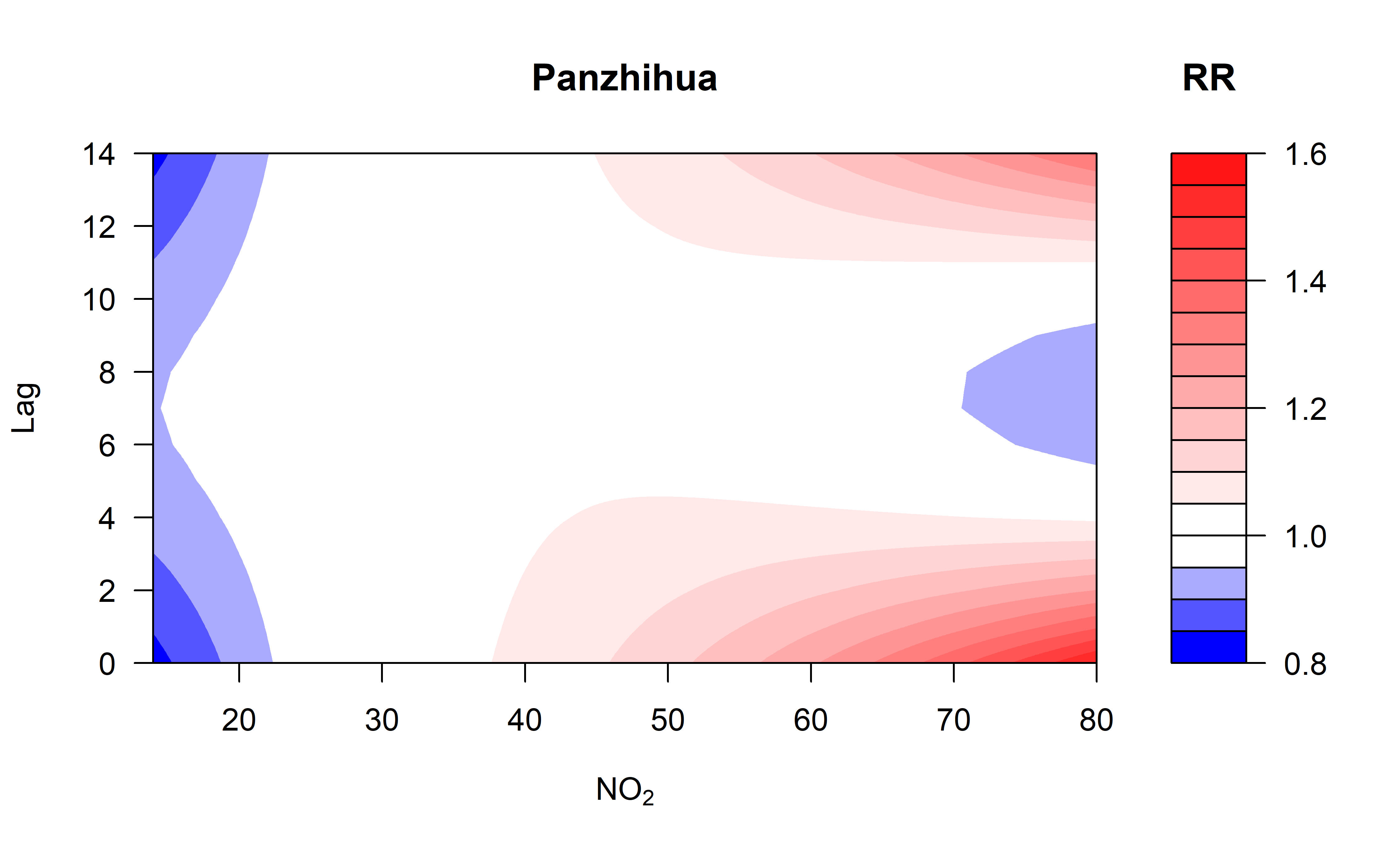

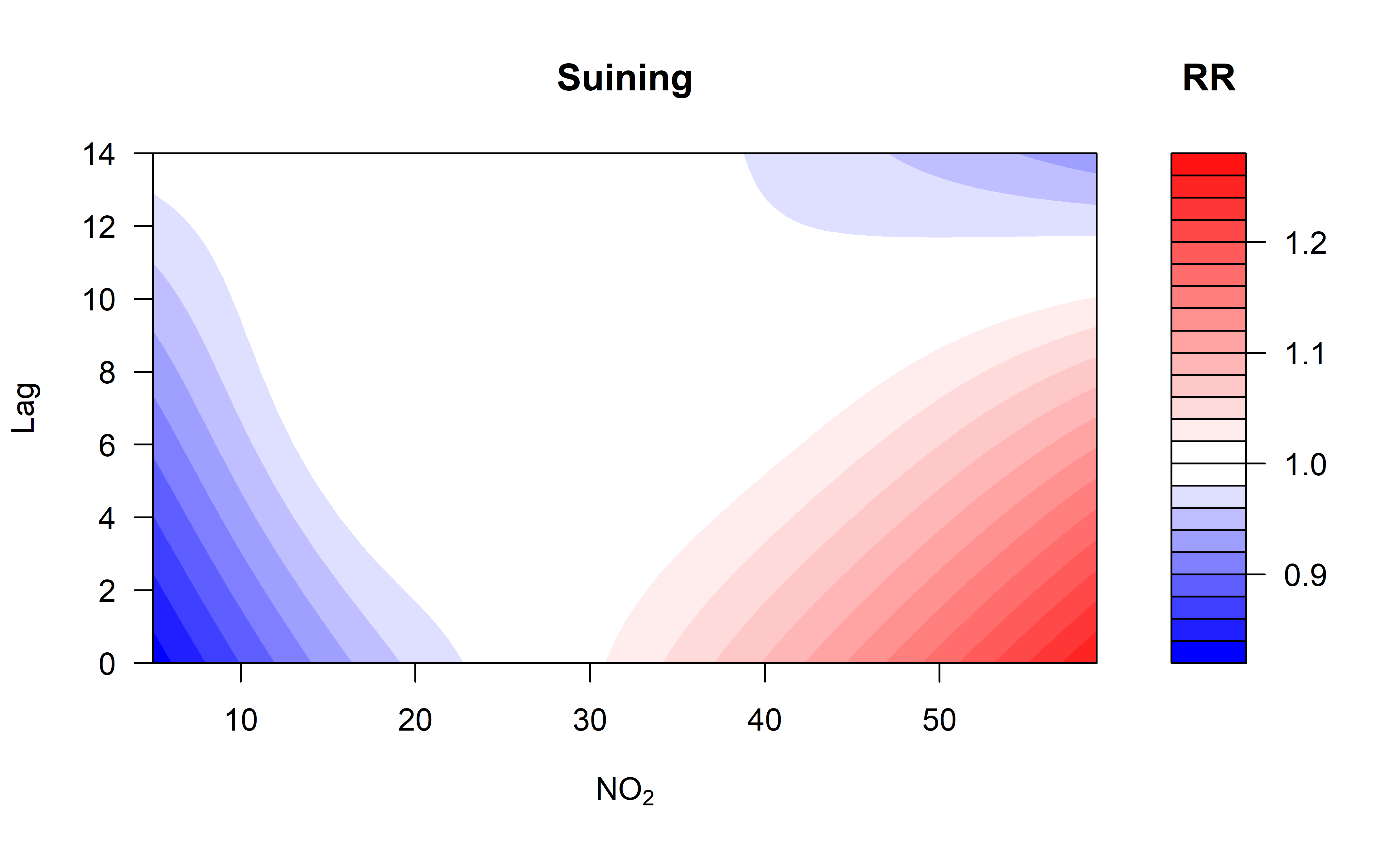

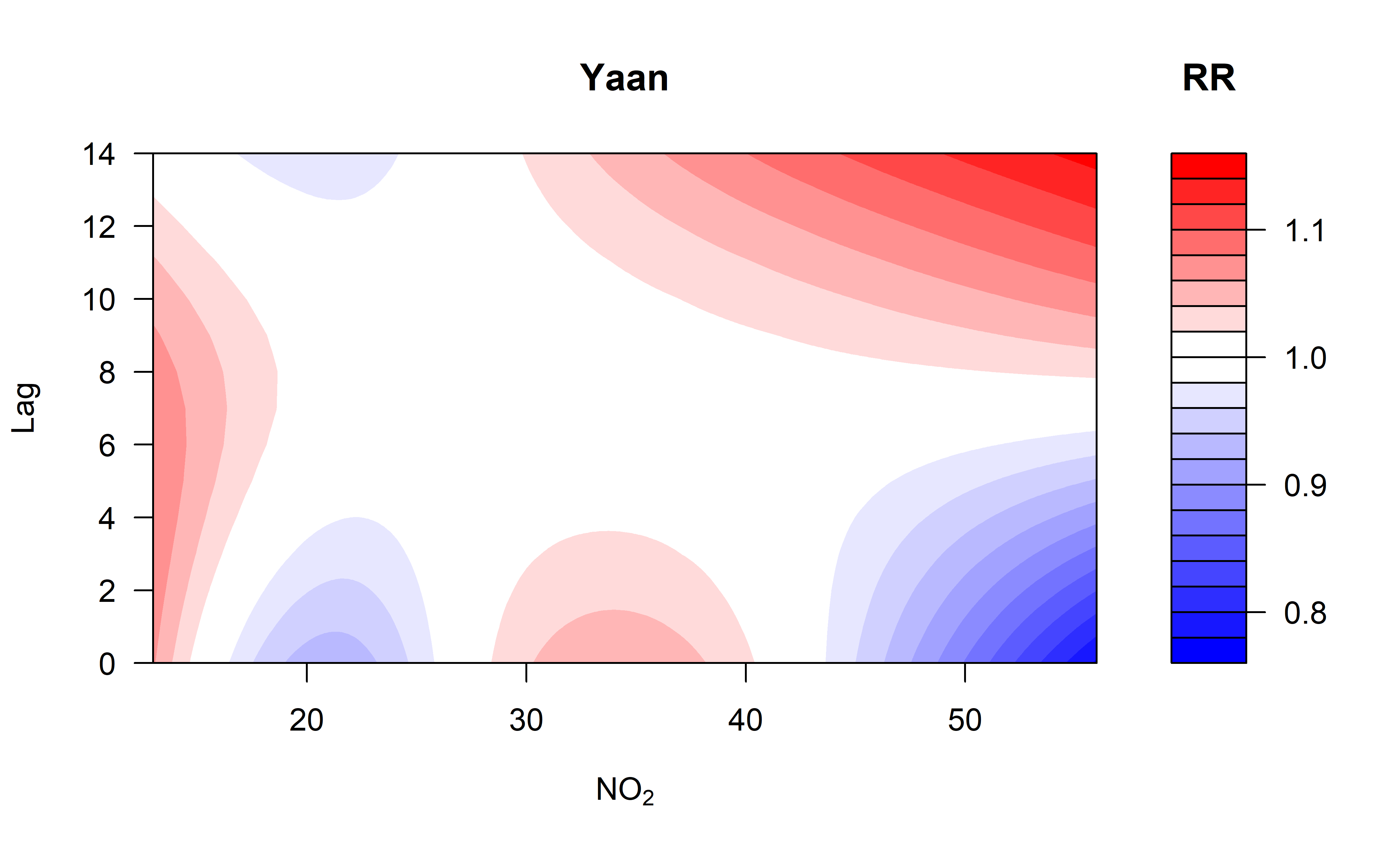

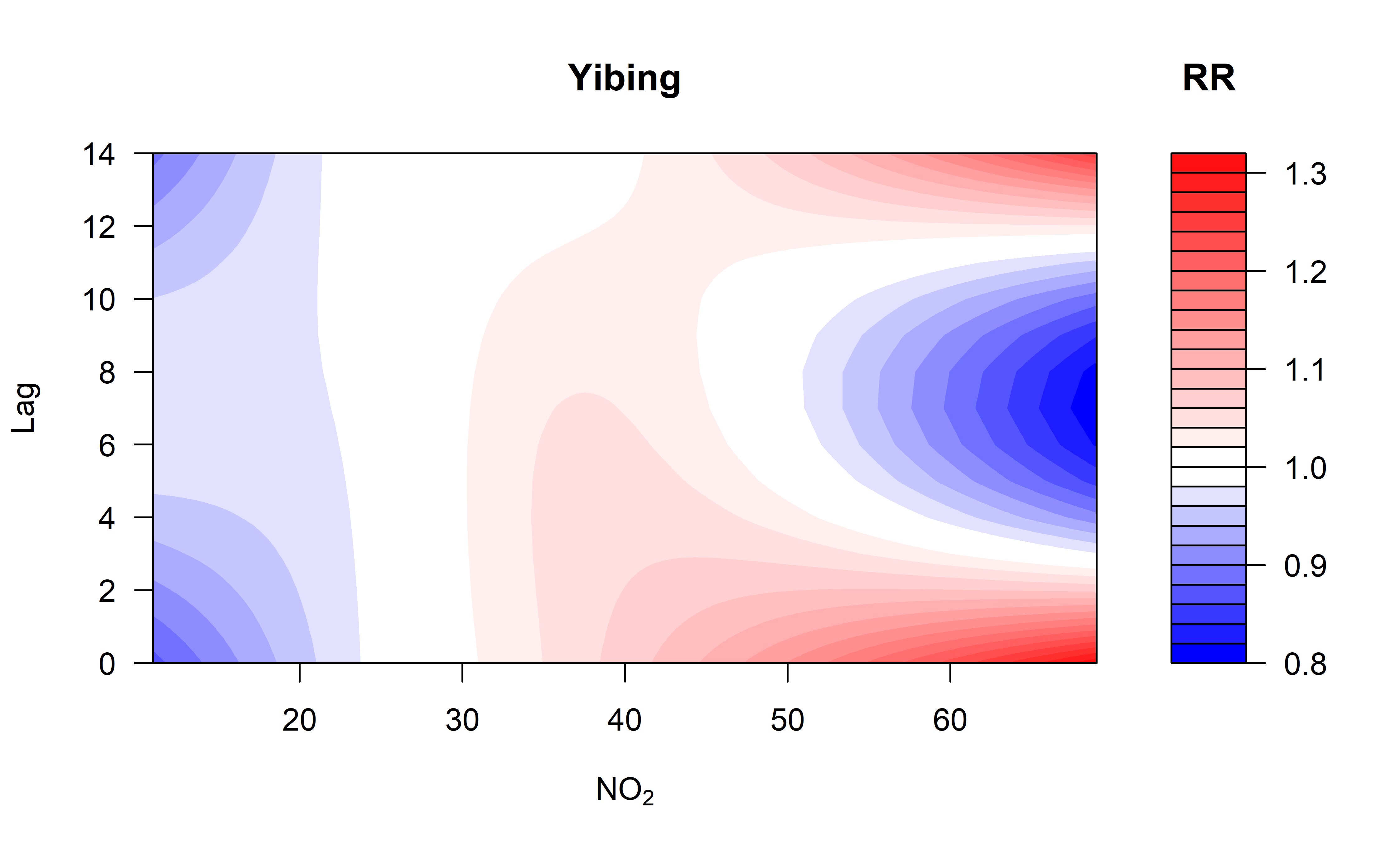

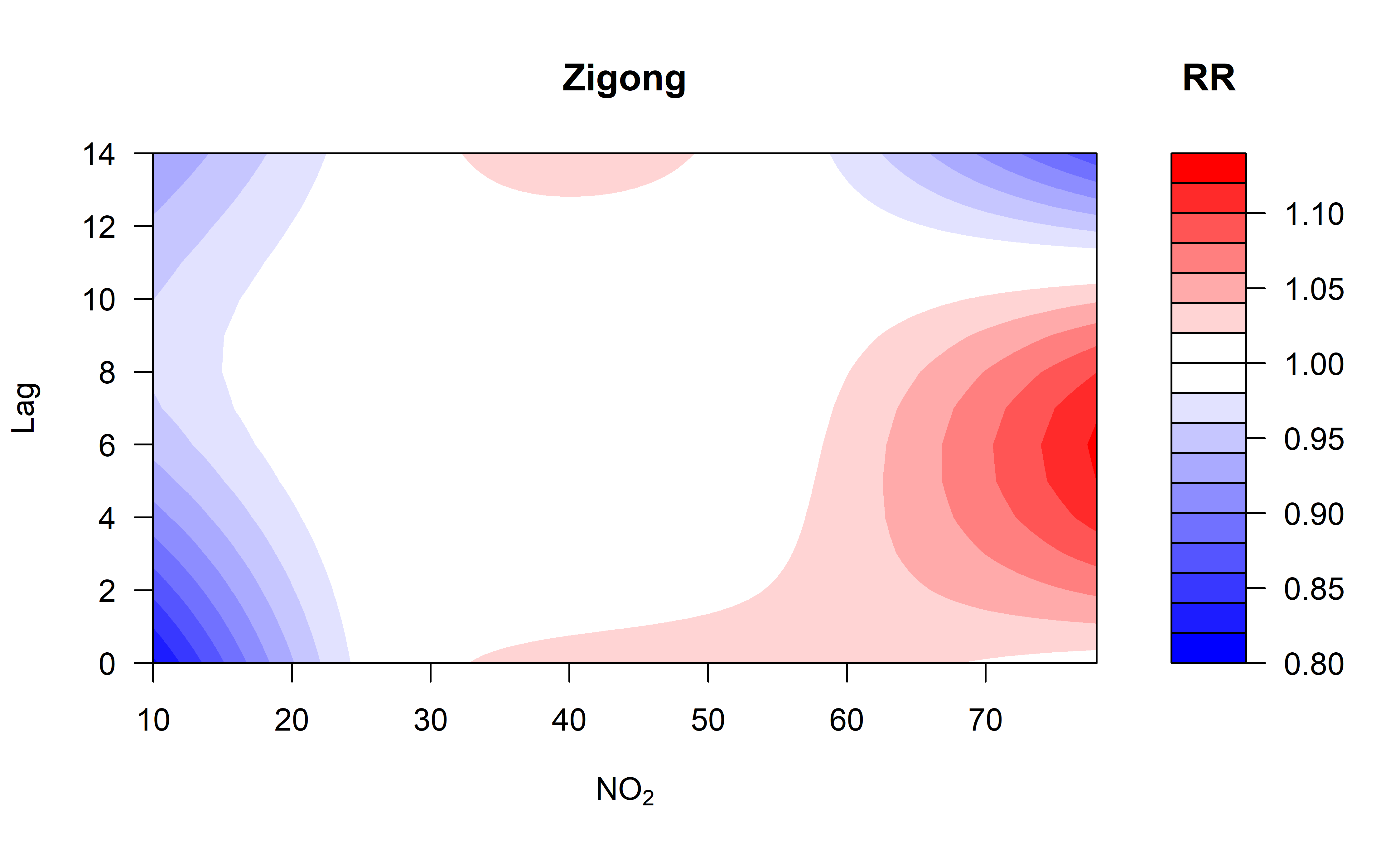

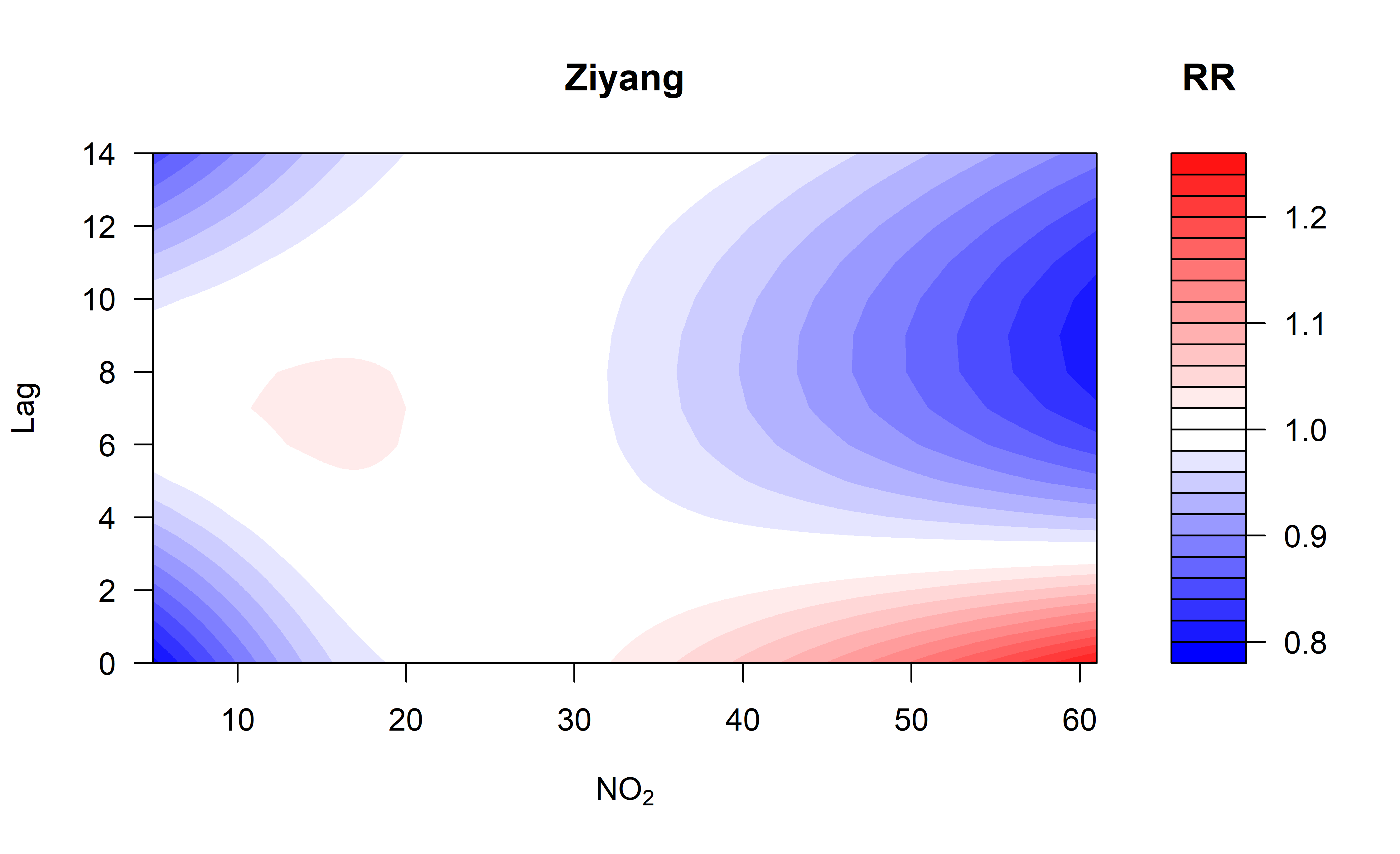


Fig. S4. Contour plots of the city-specific relationship between the risk of HFMD and NO_2_ at different time lags.


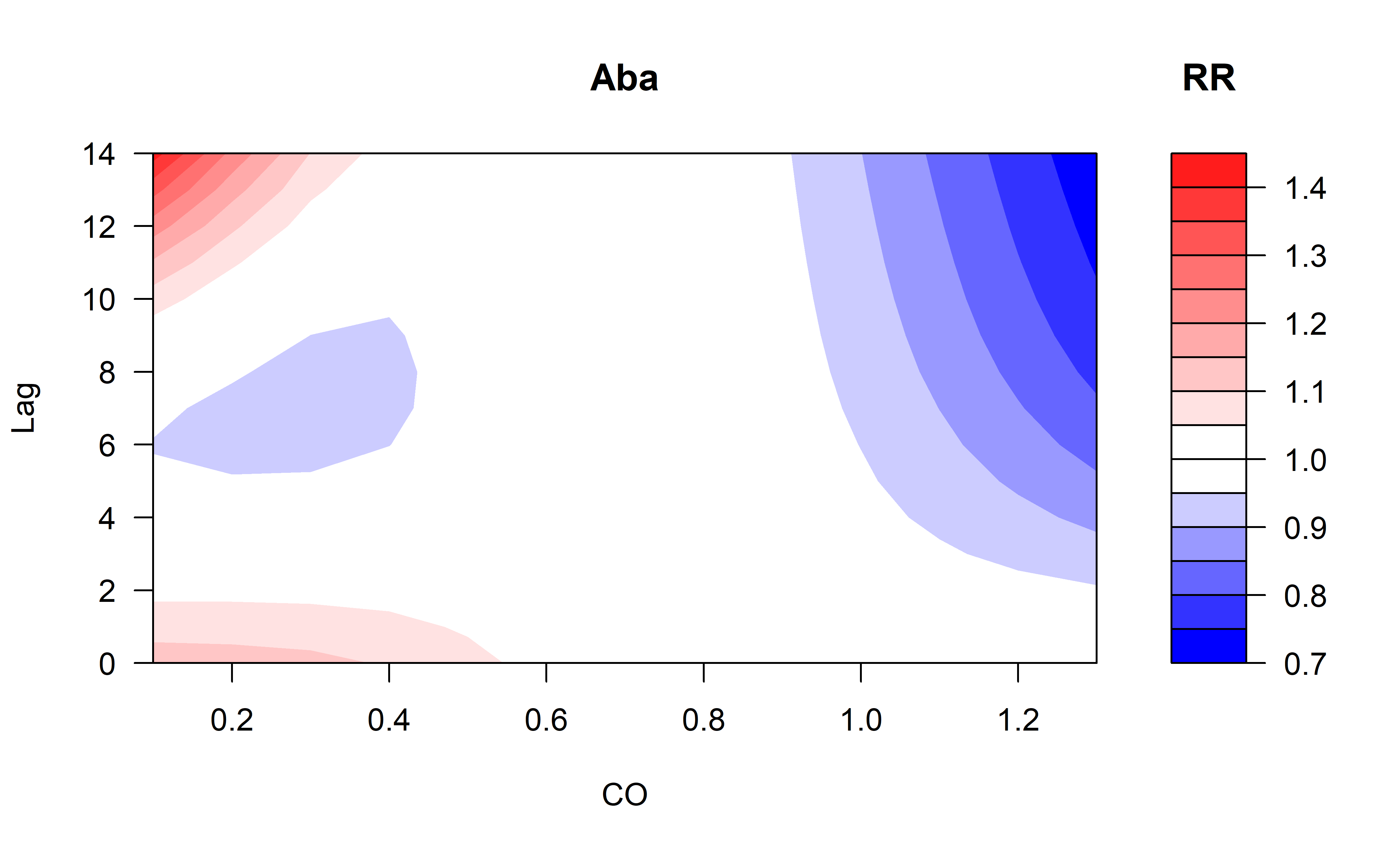

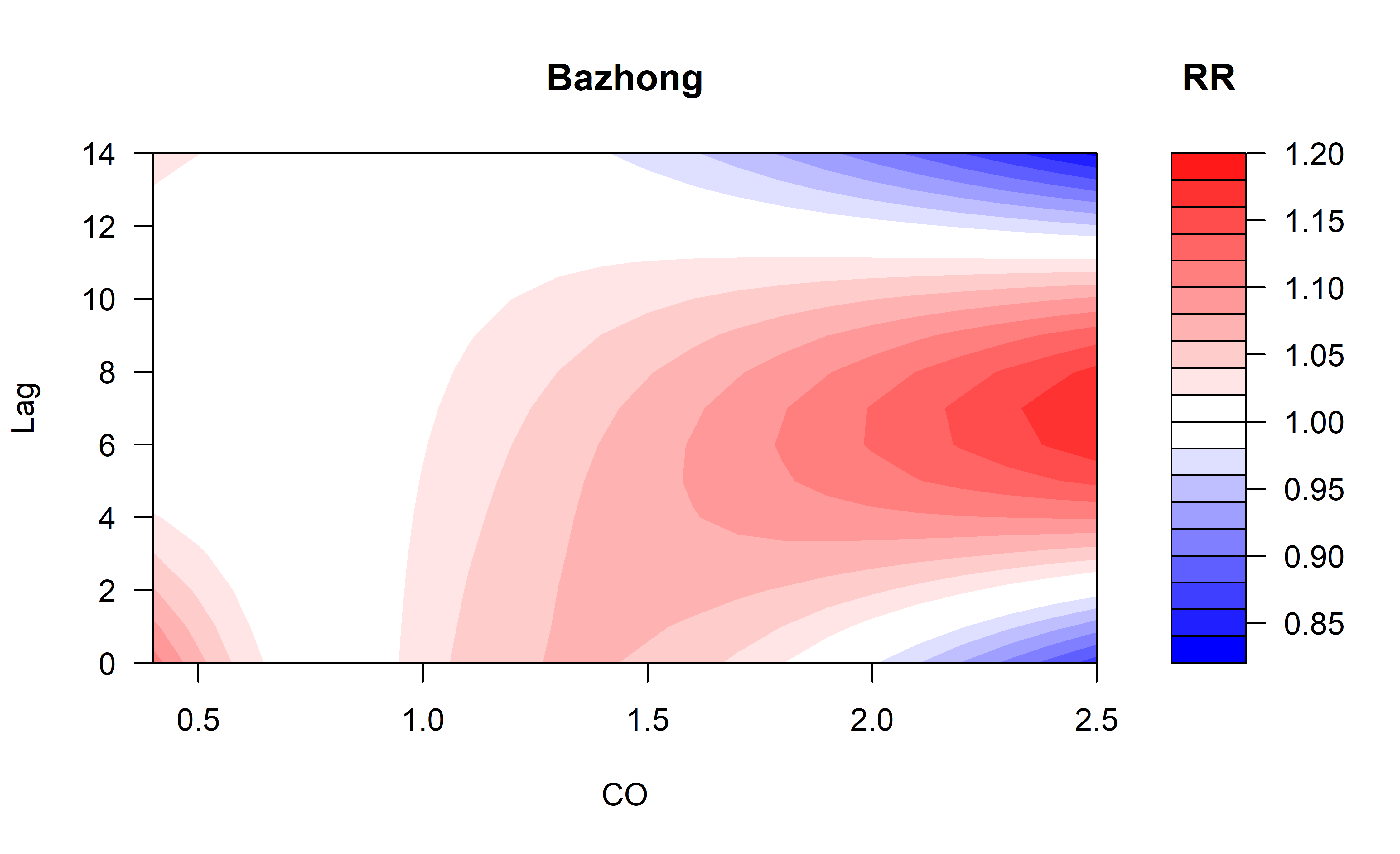

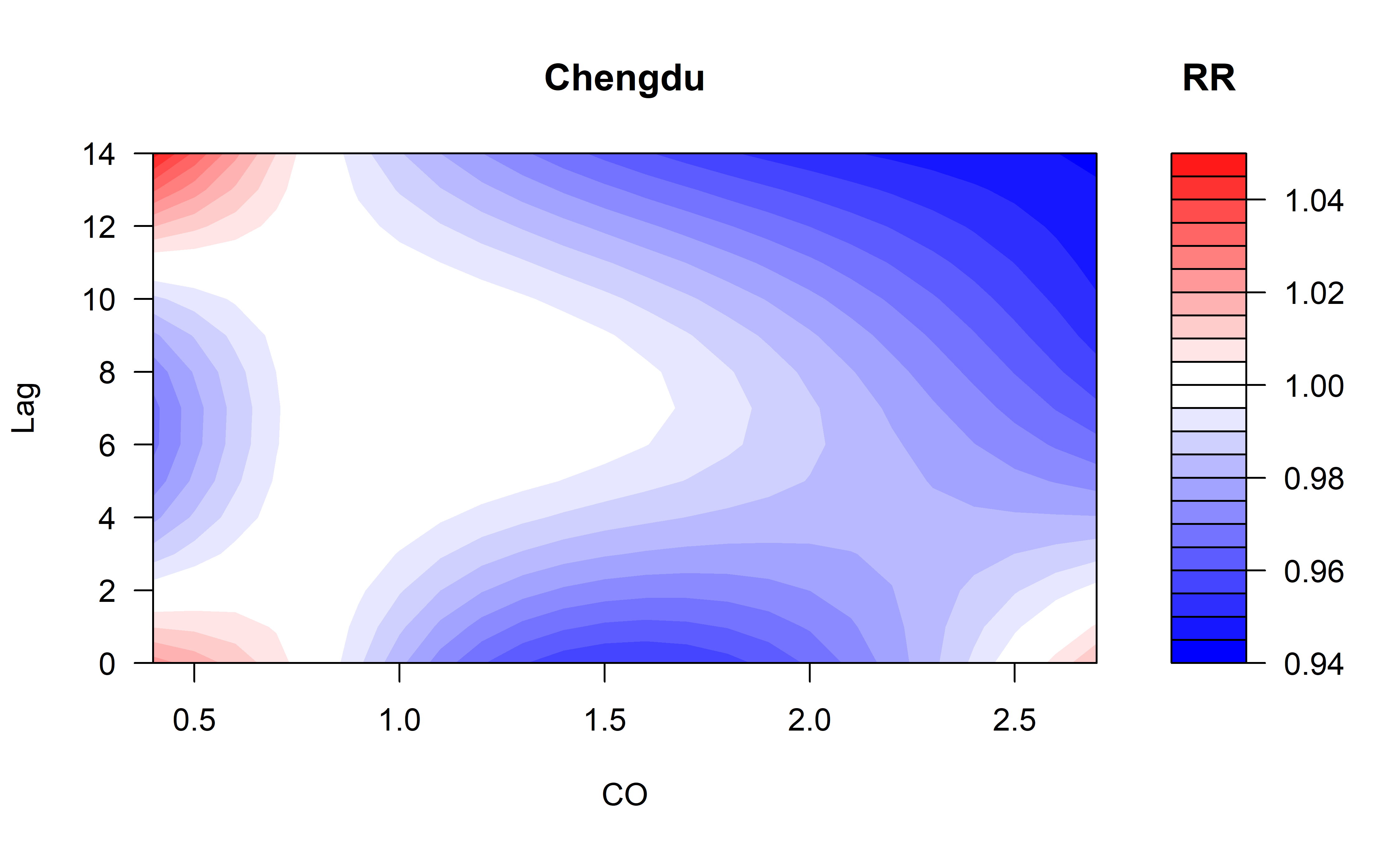

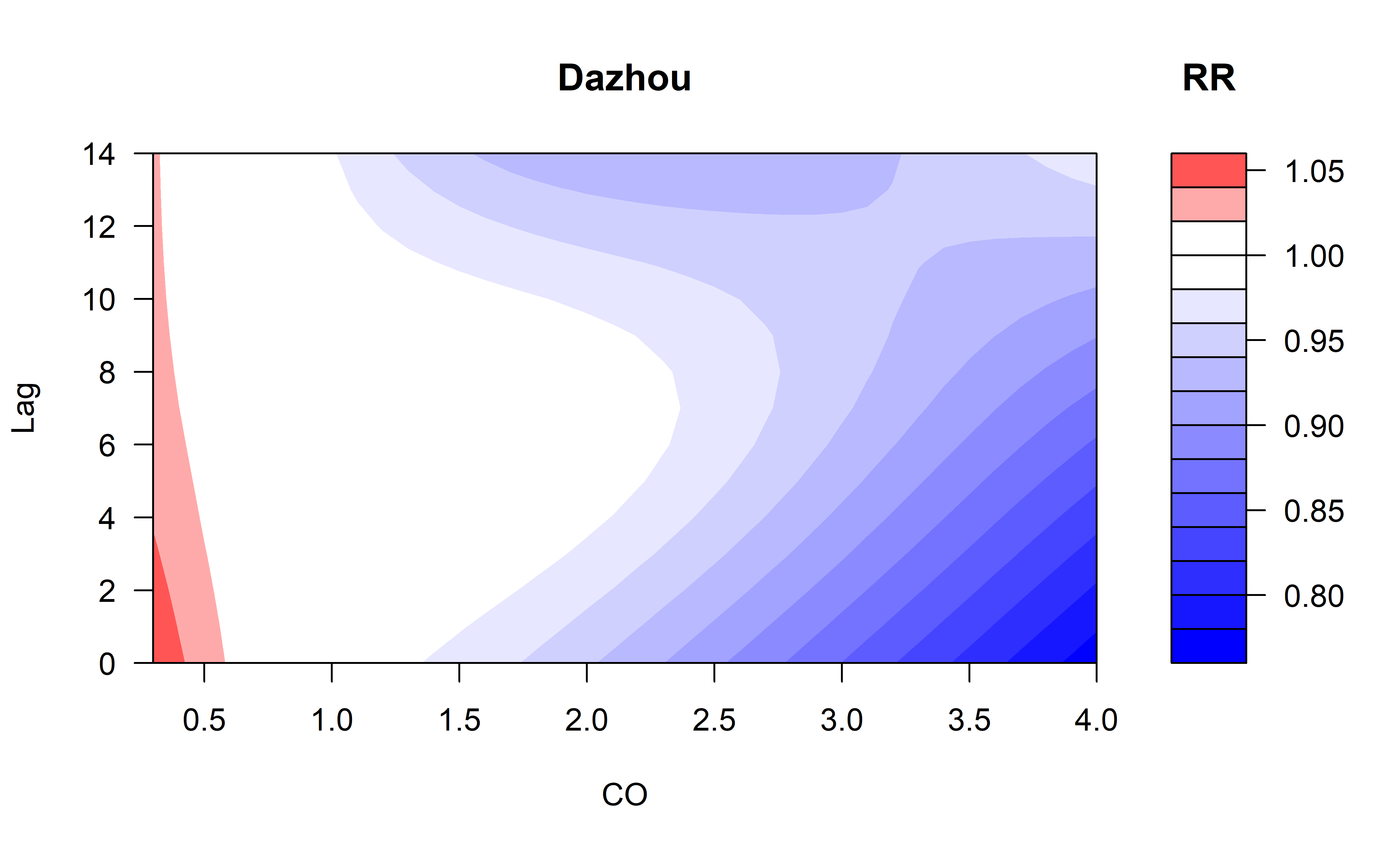

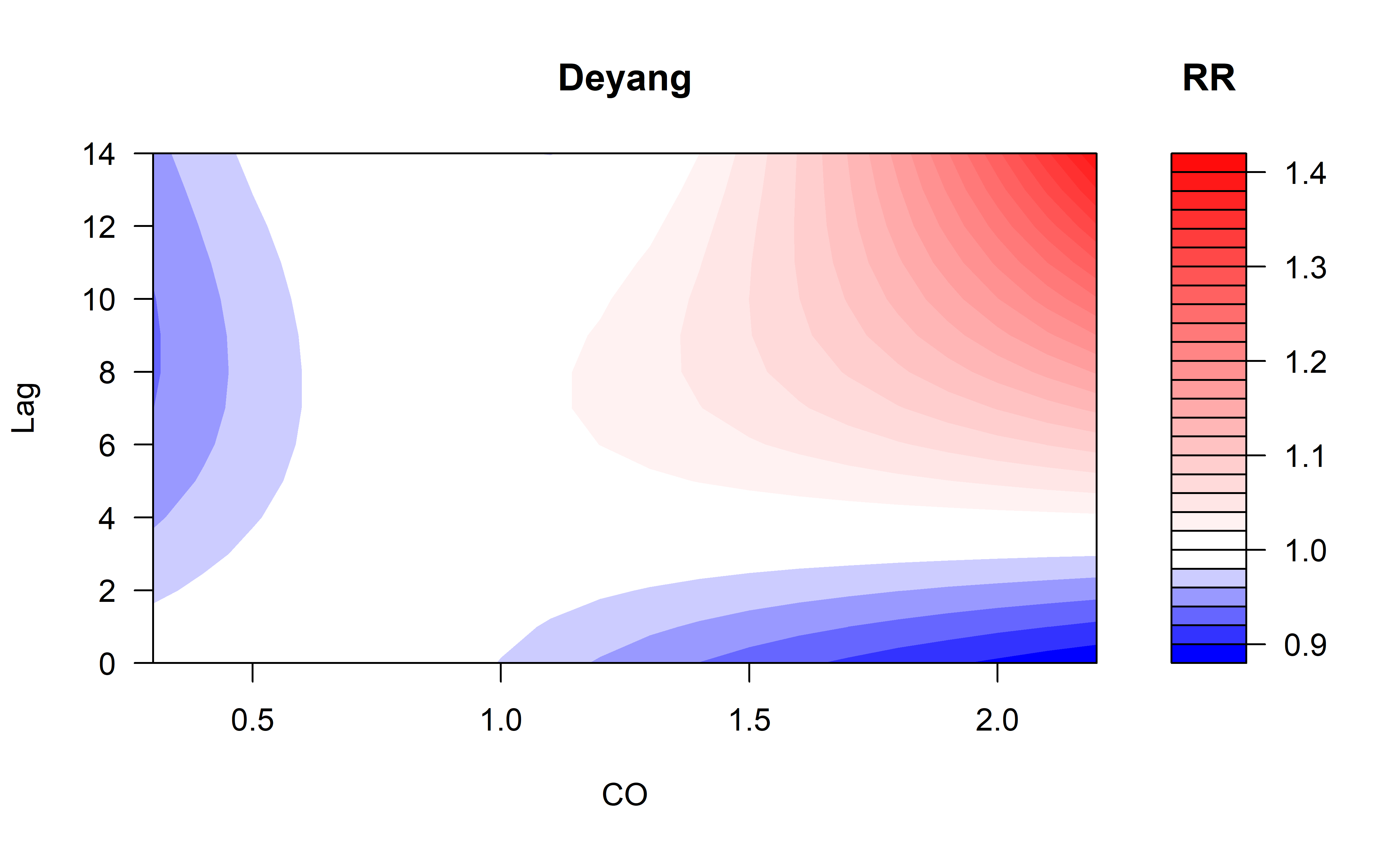

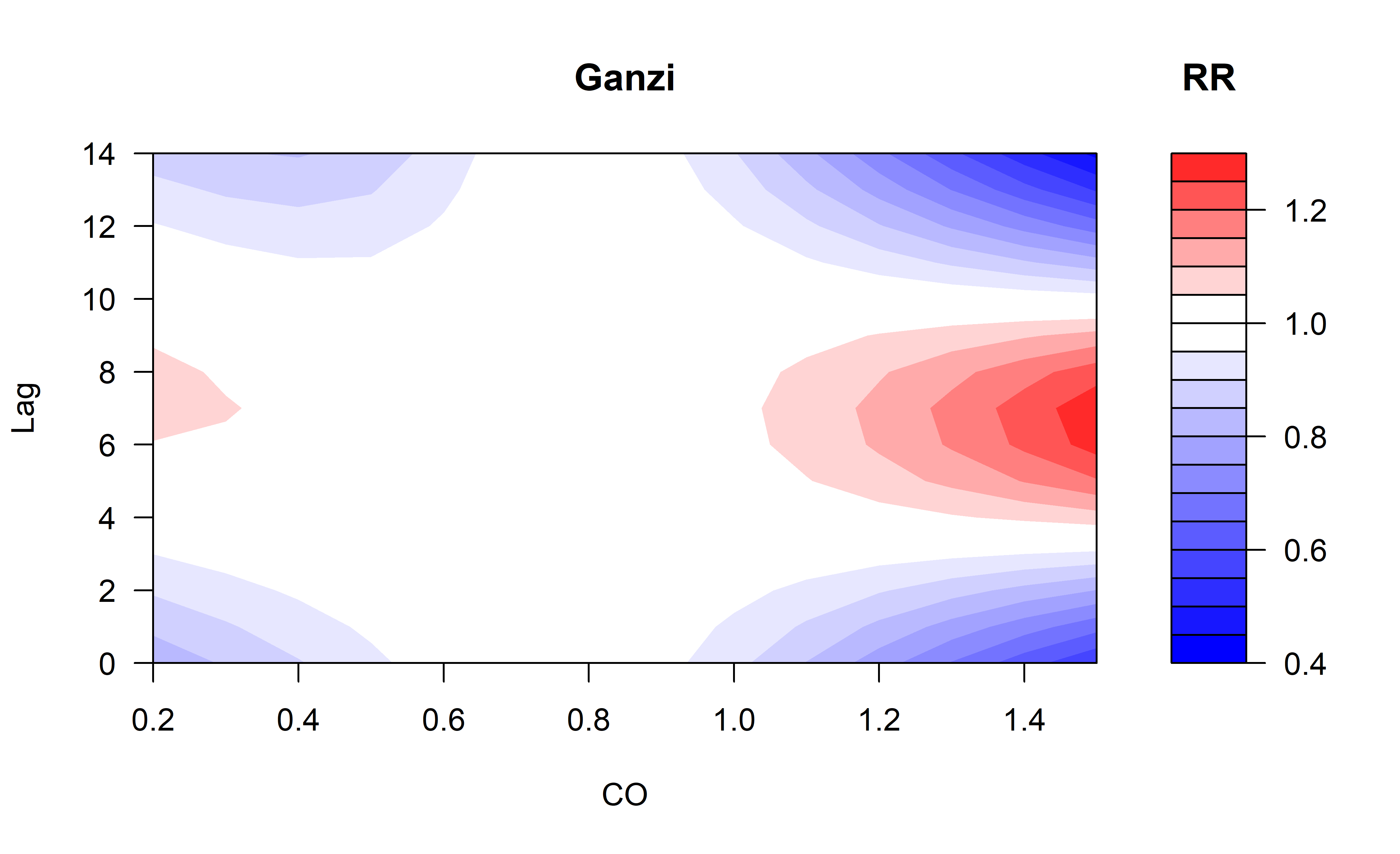

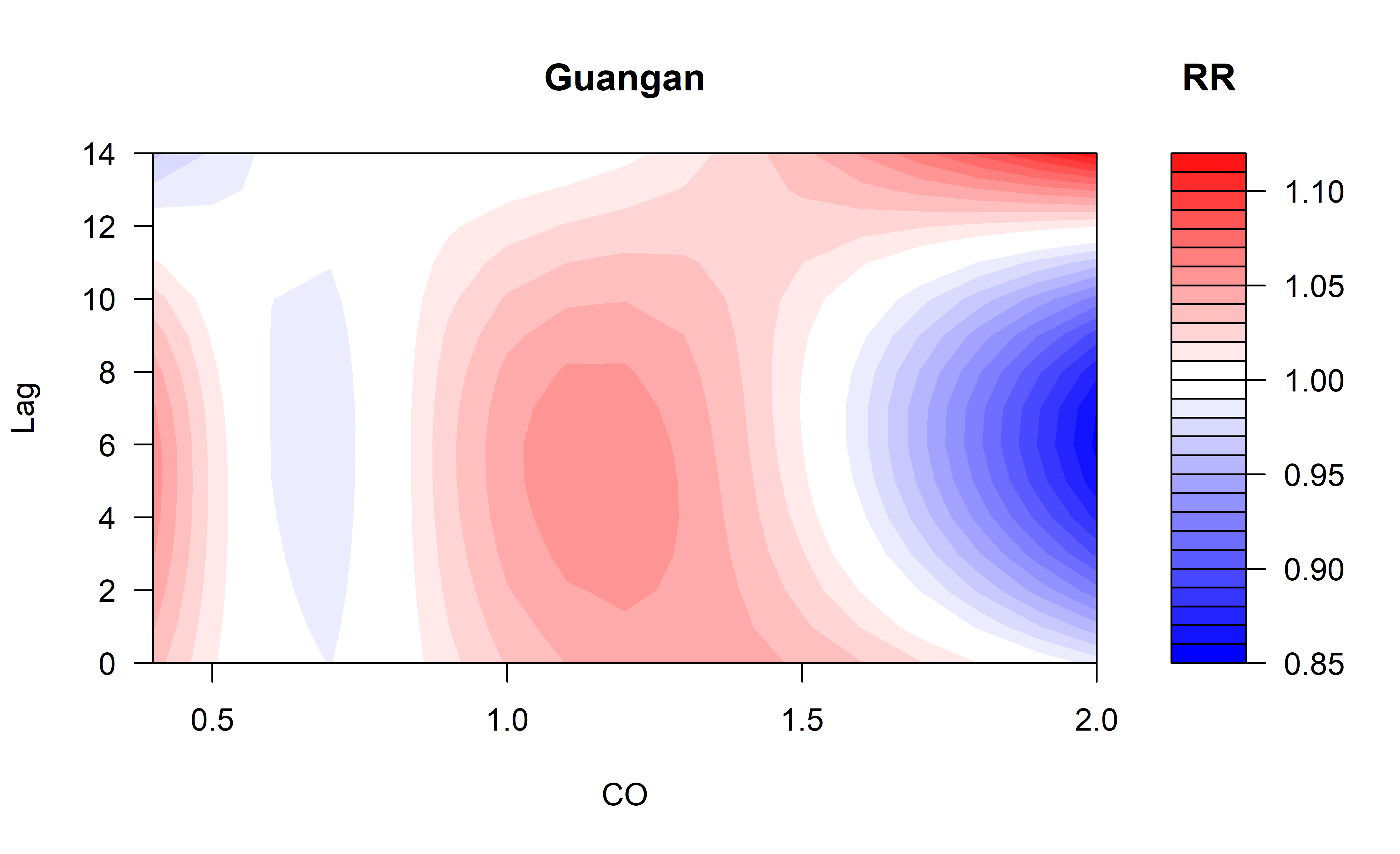

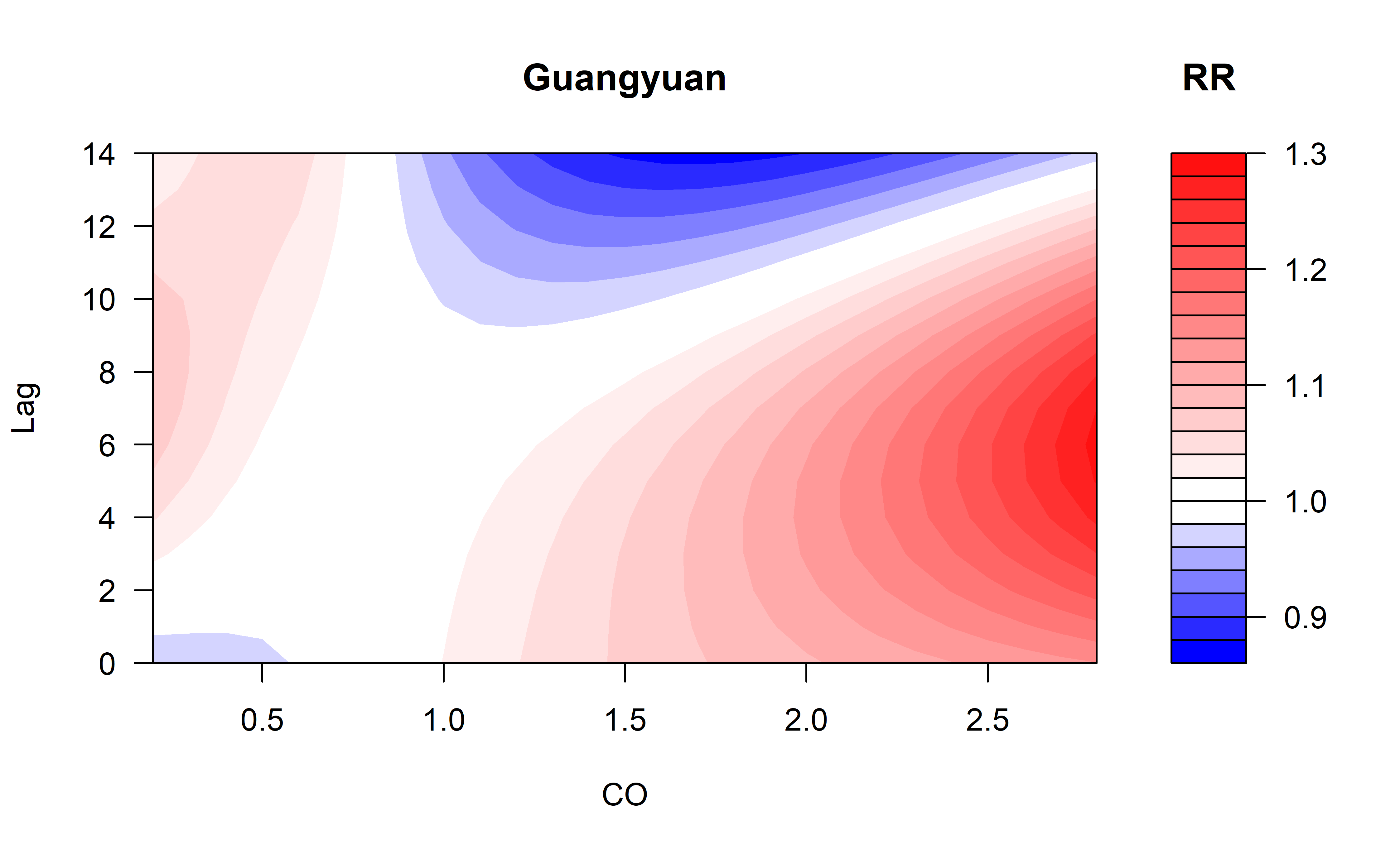

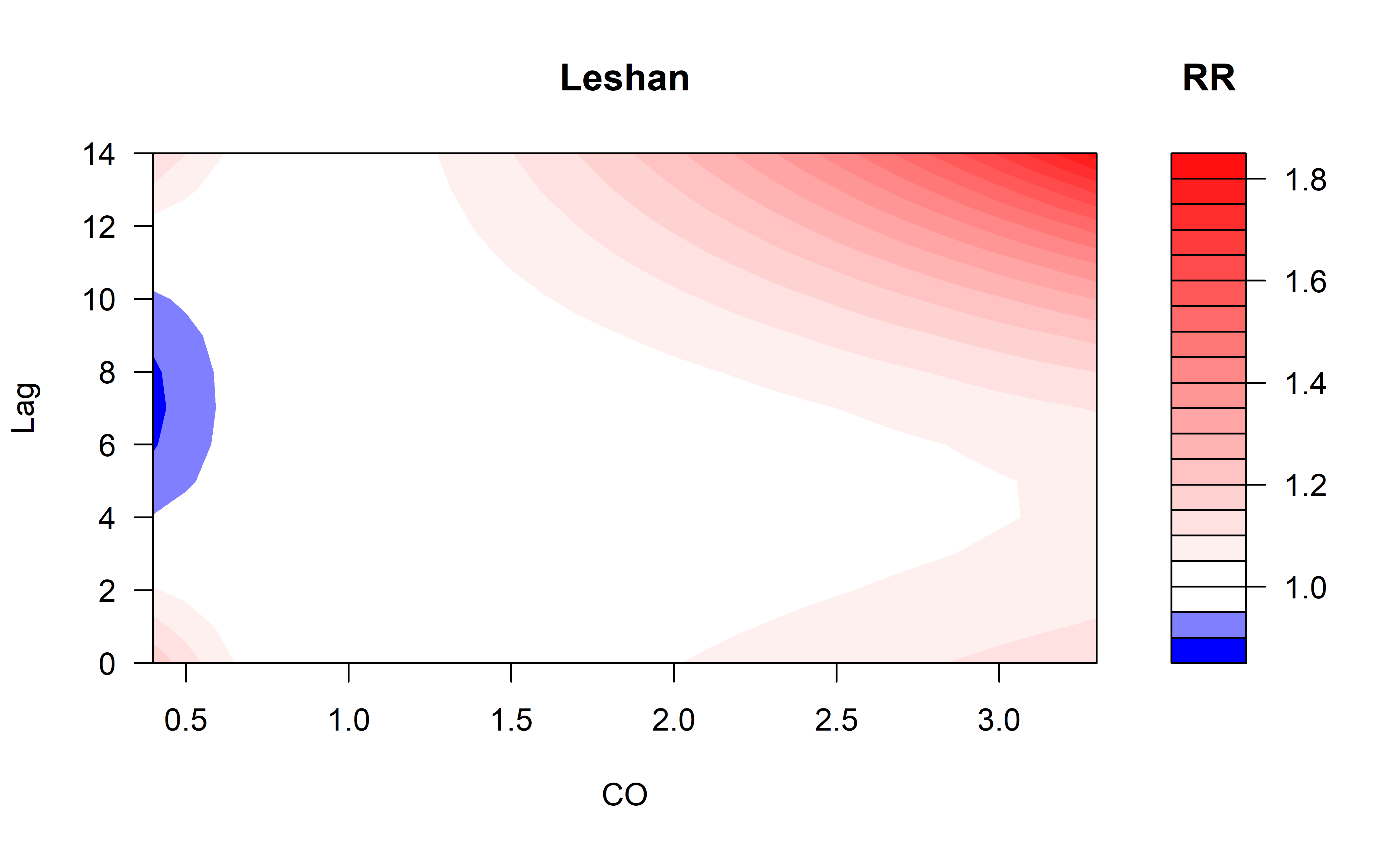

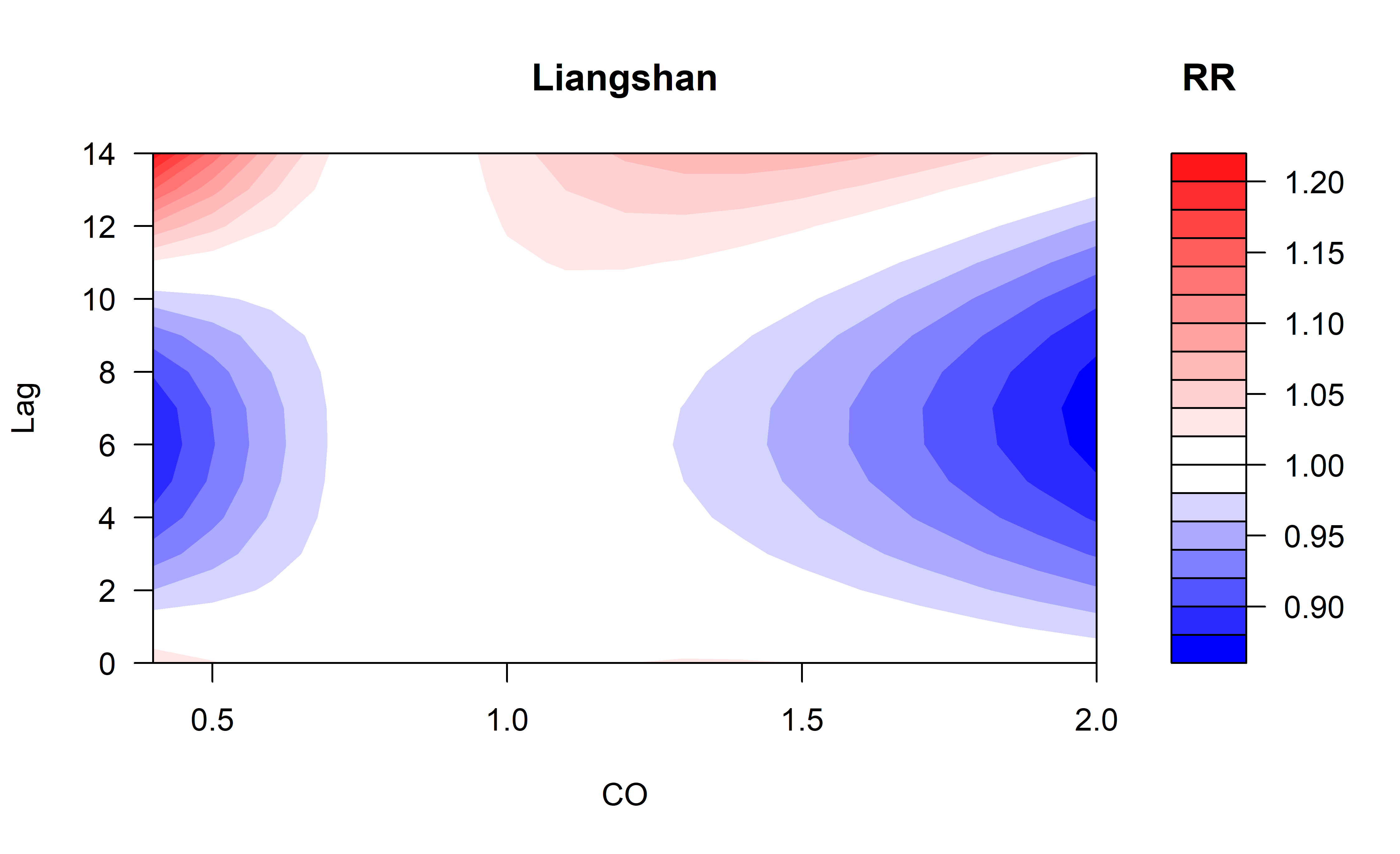

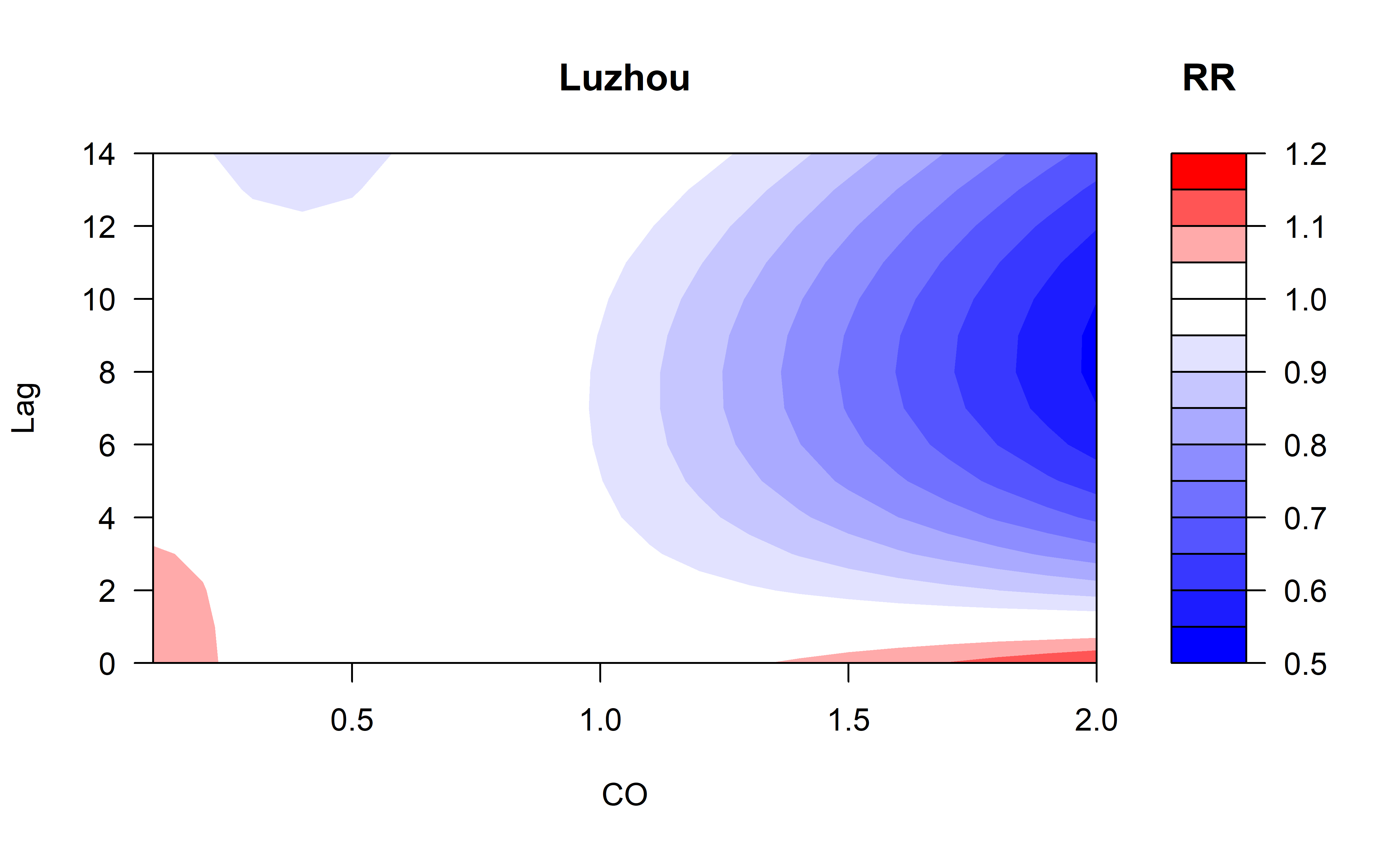

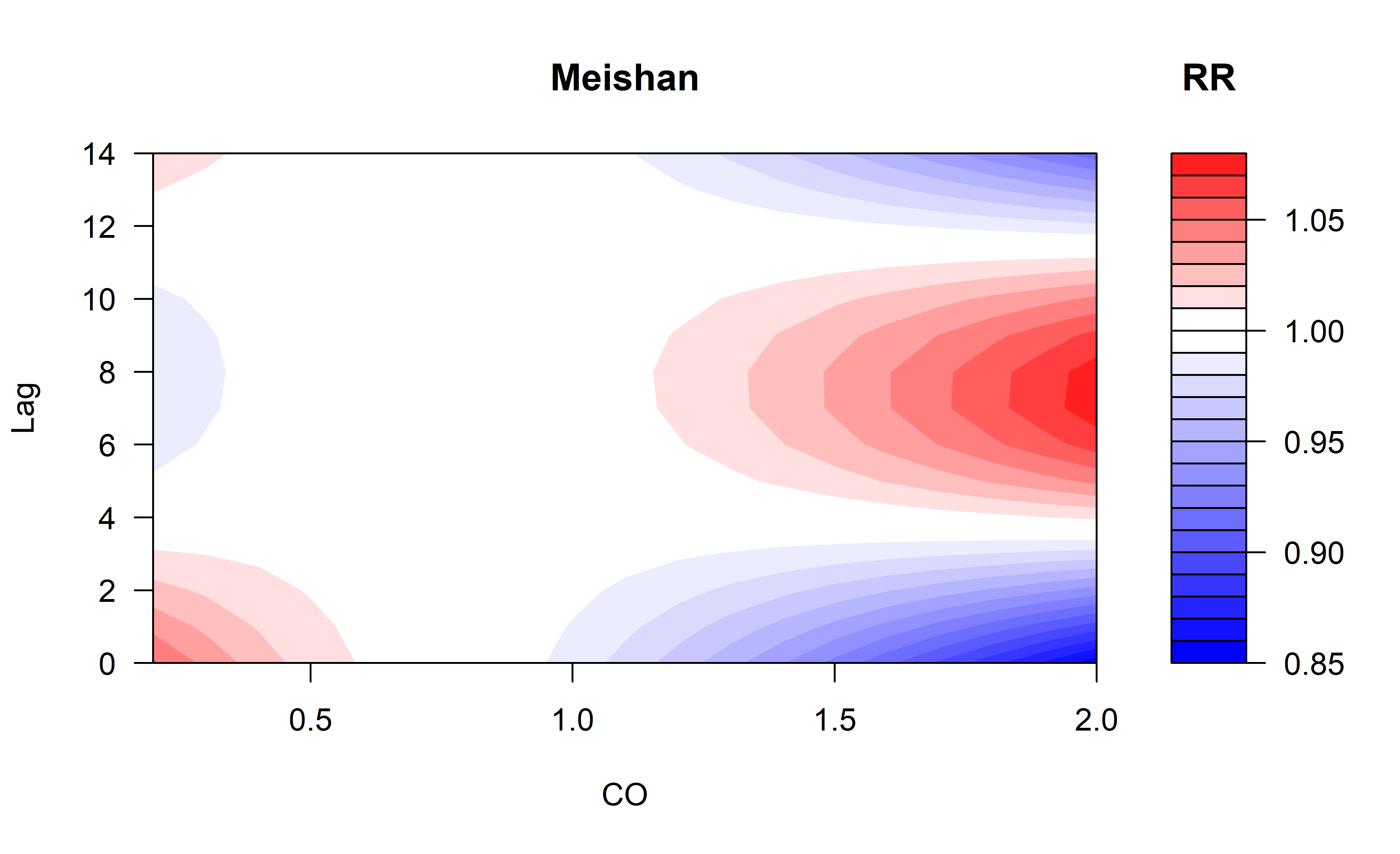

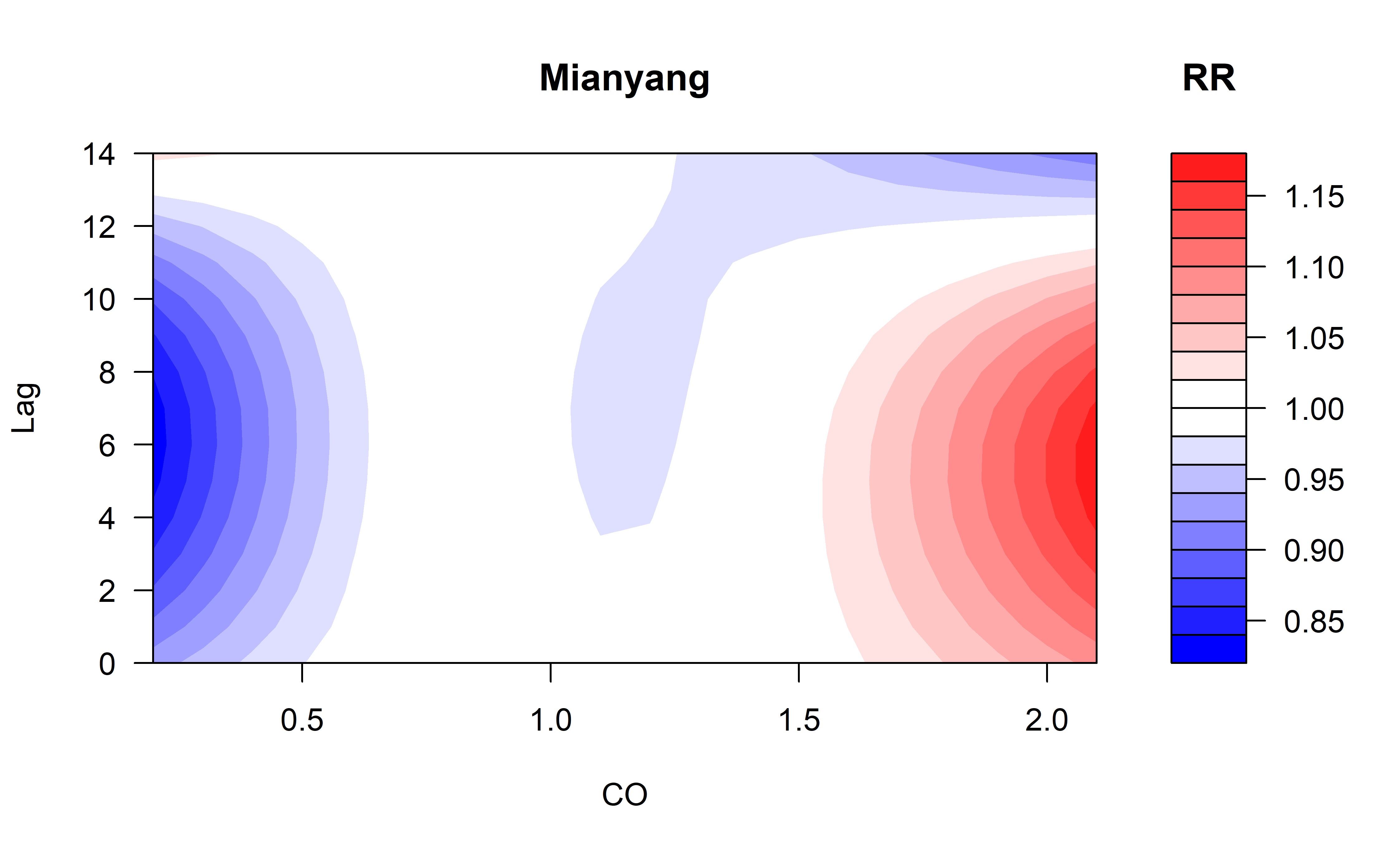

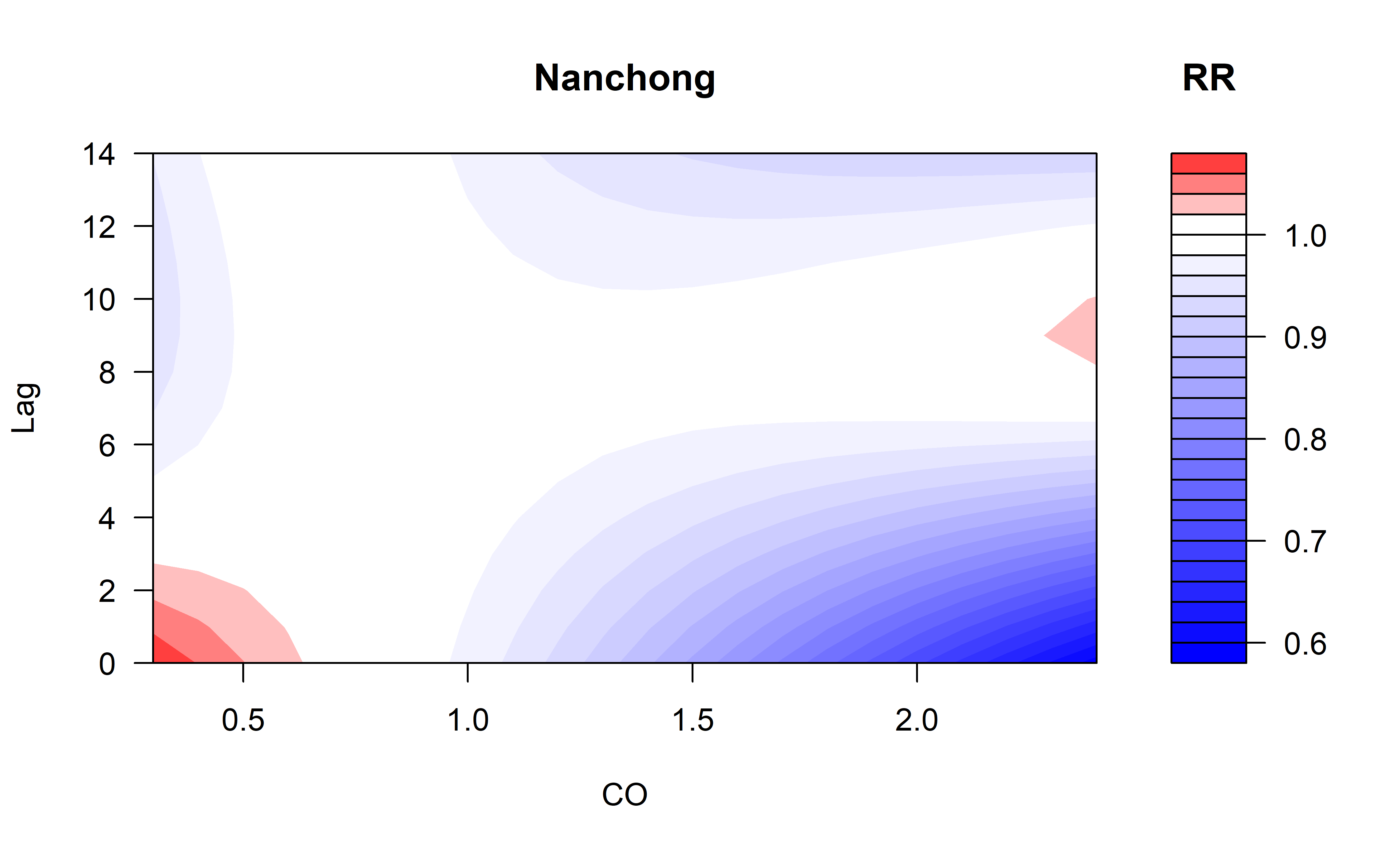

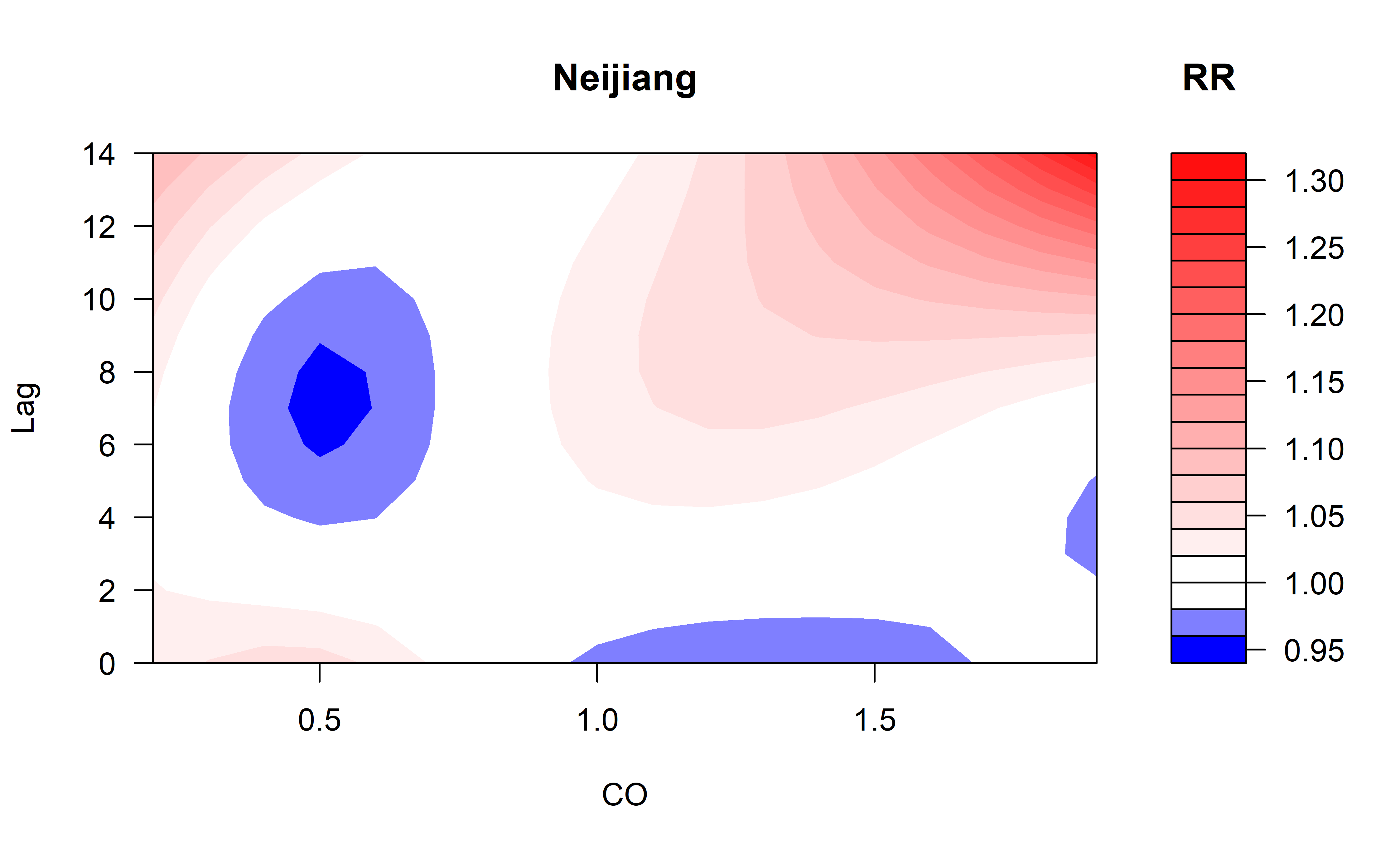

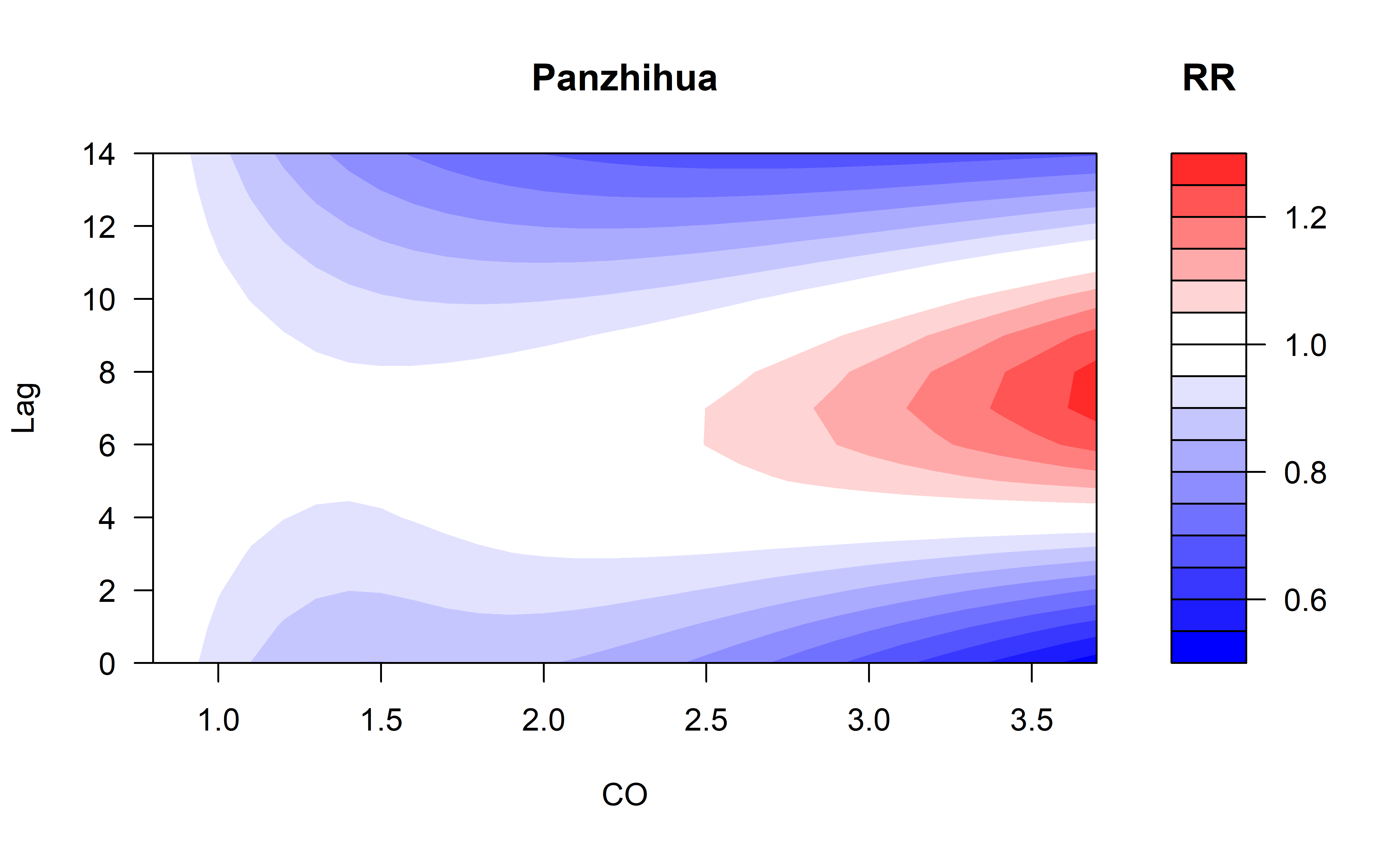

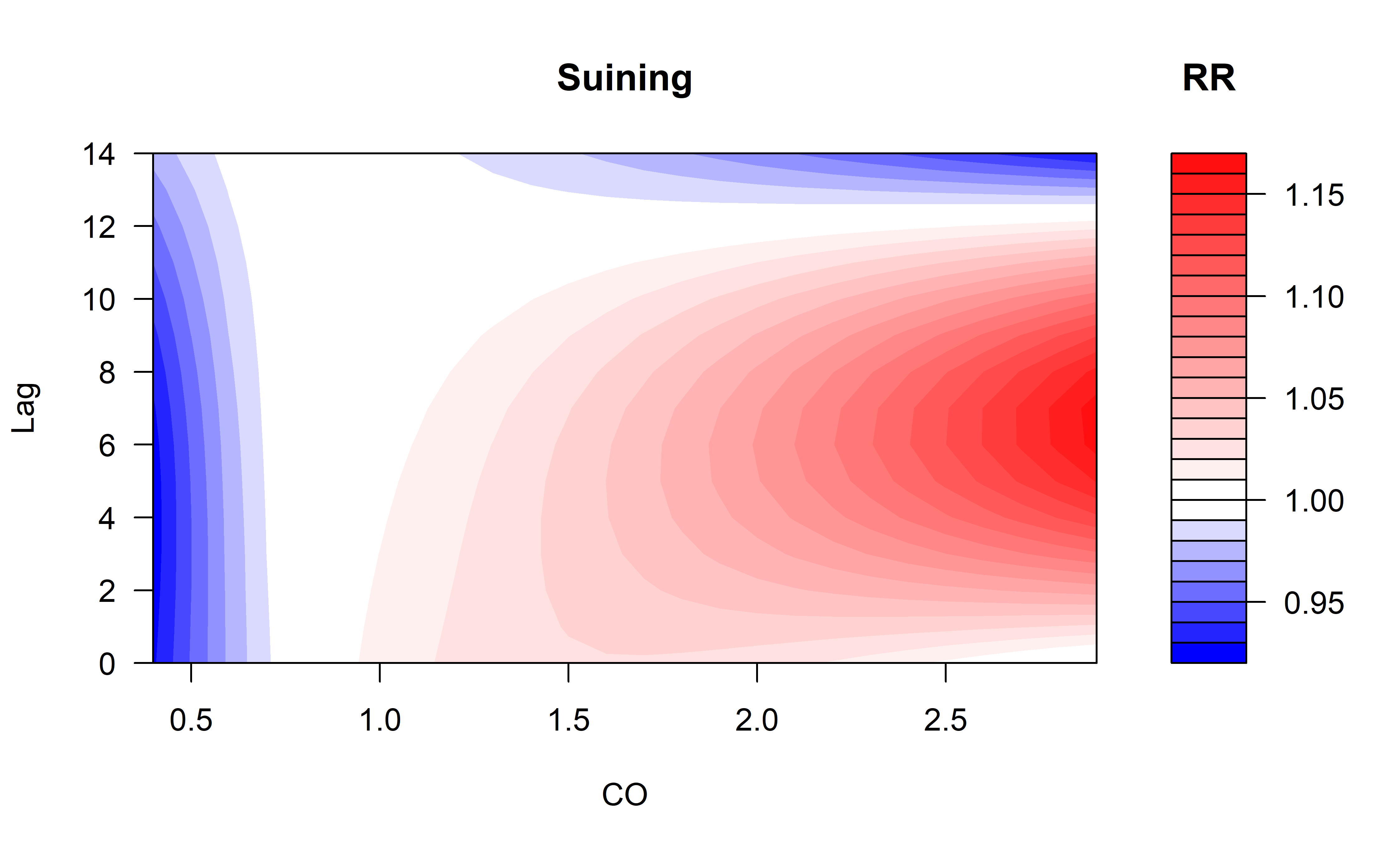

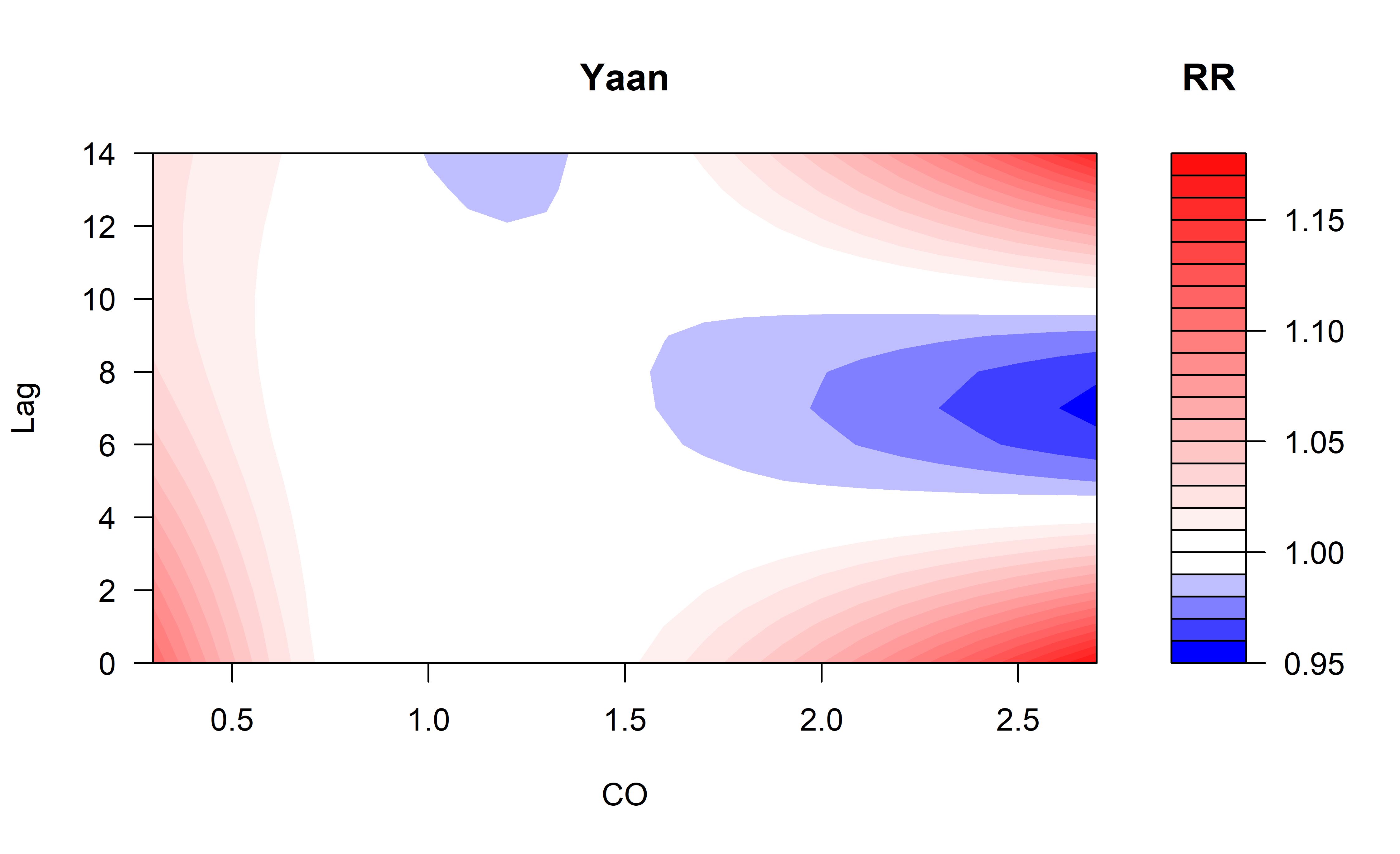

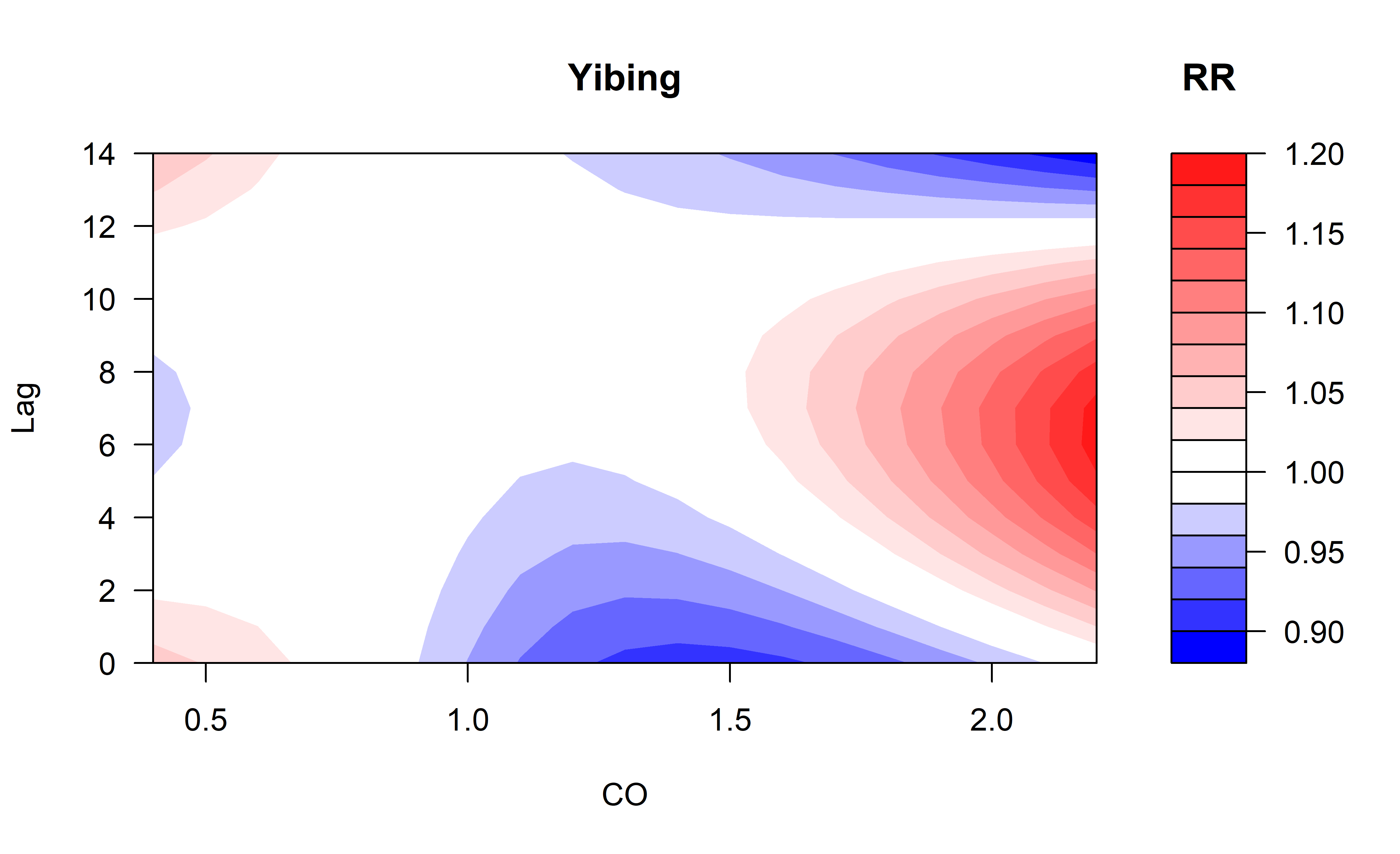

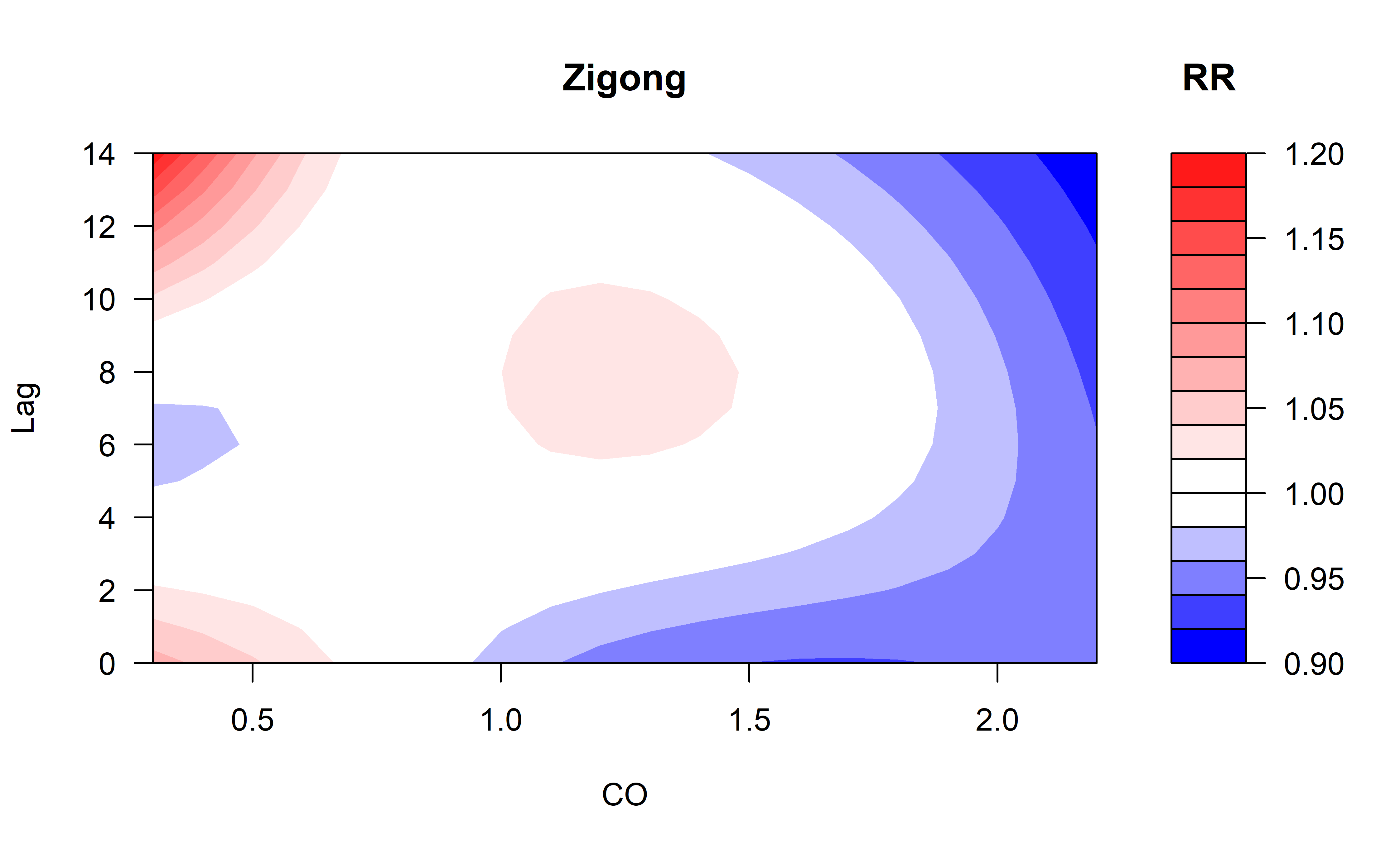

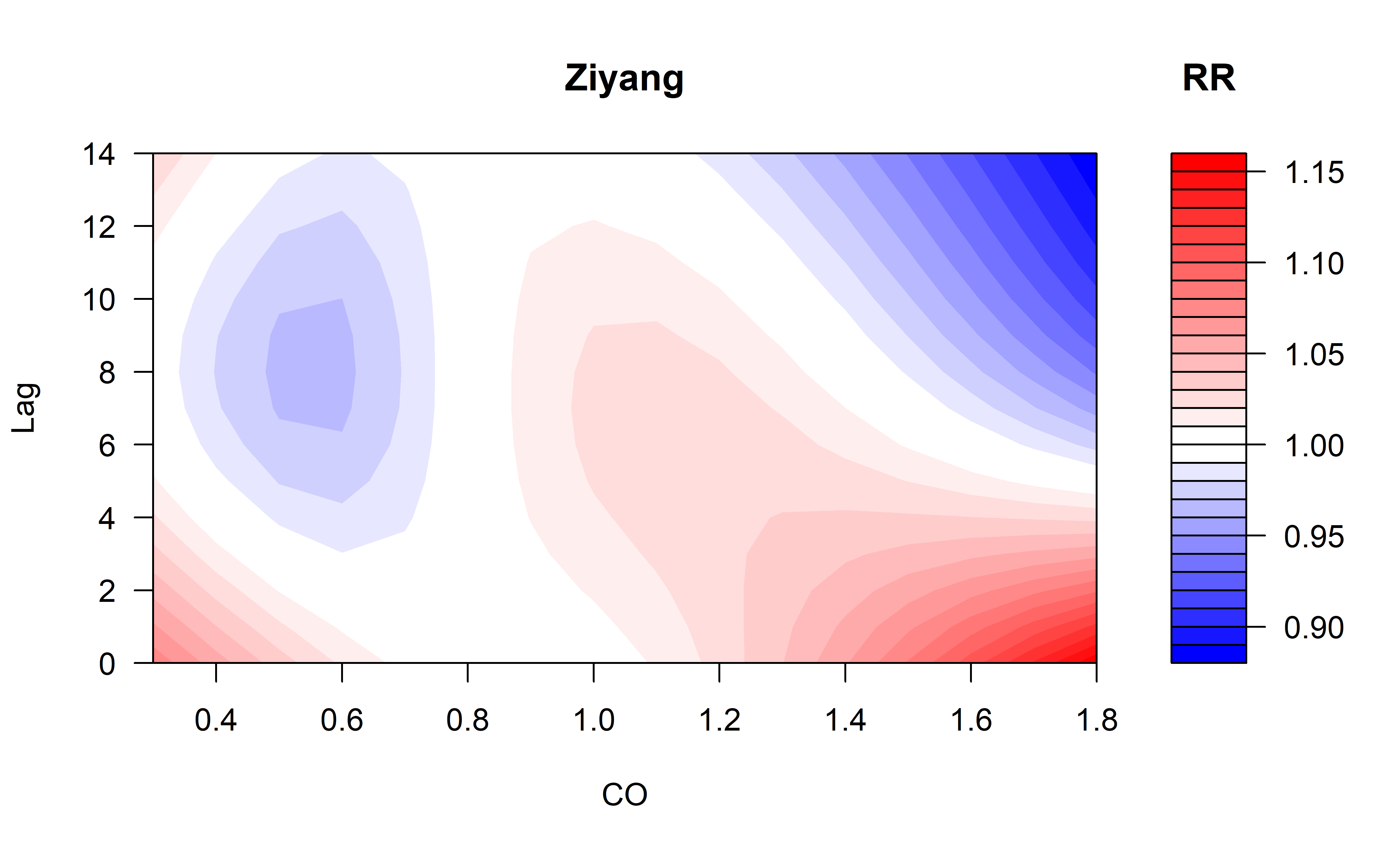


Fig. S5. Contour plots of the city-specific relationship between the risk of HFMD and CO at different time lags.


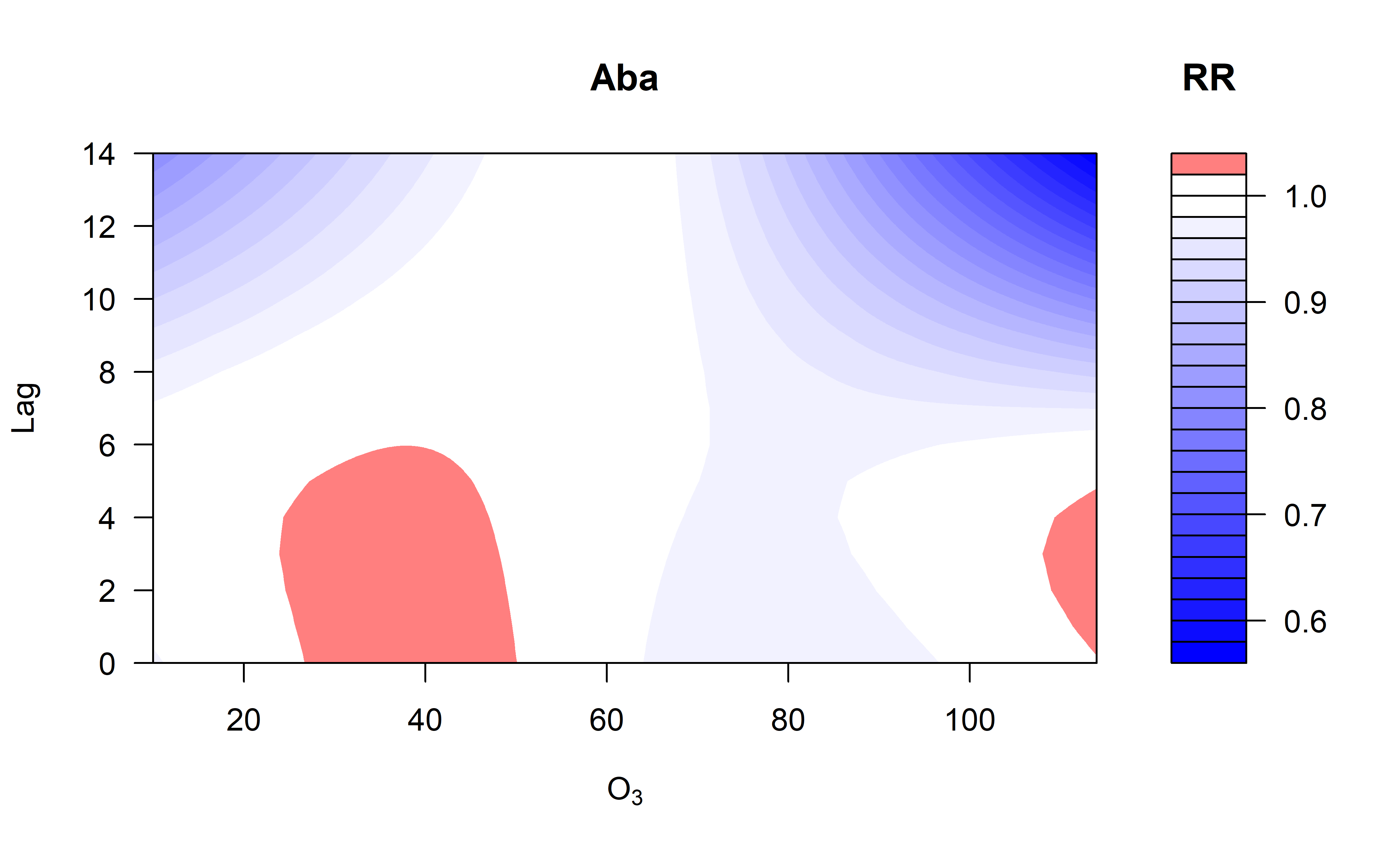

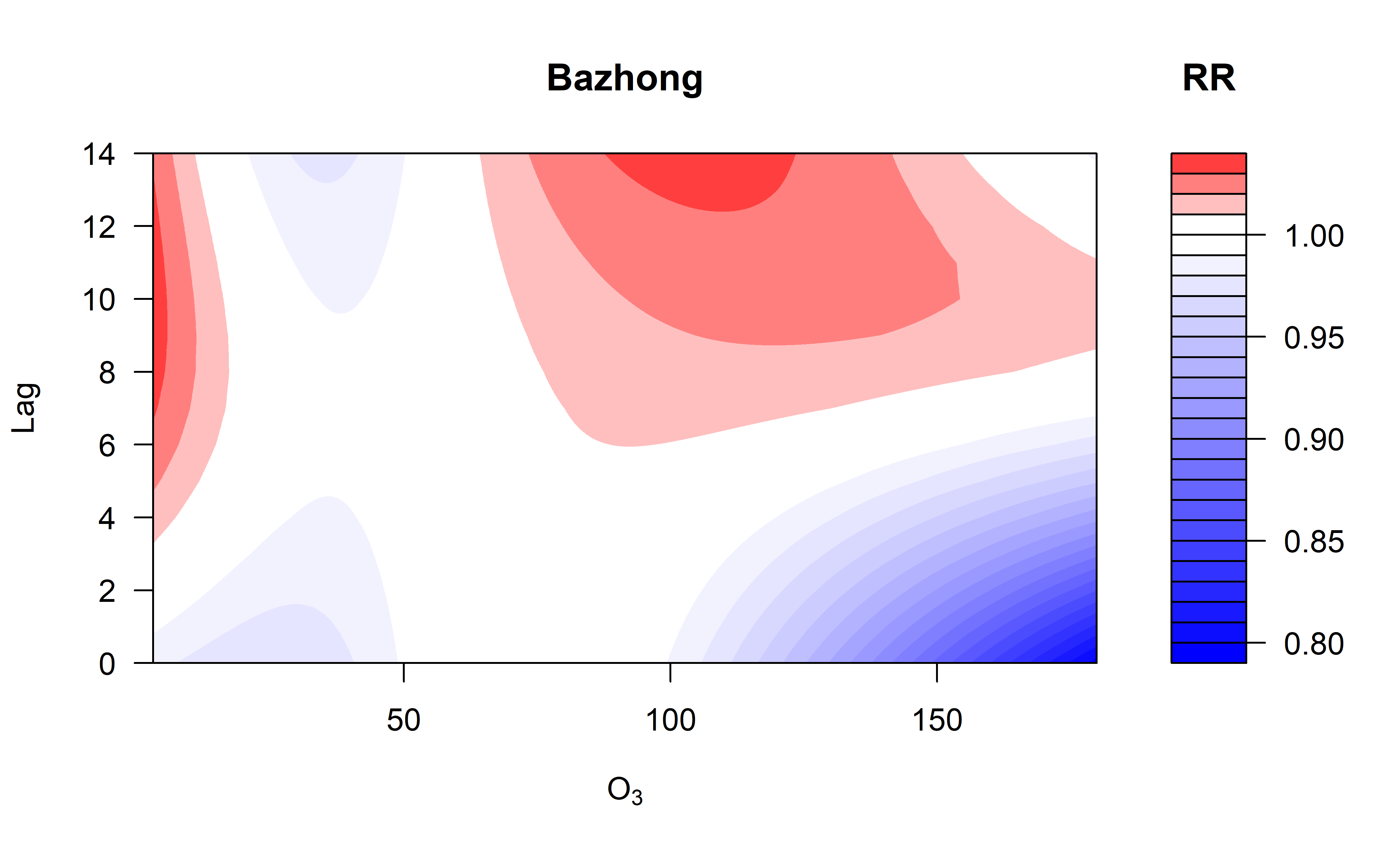

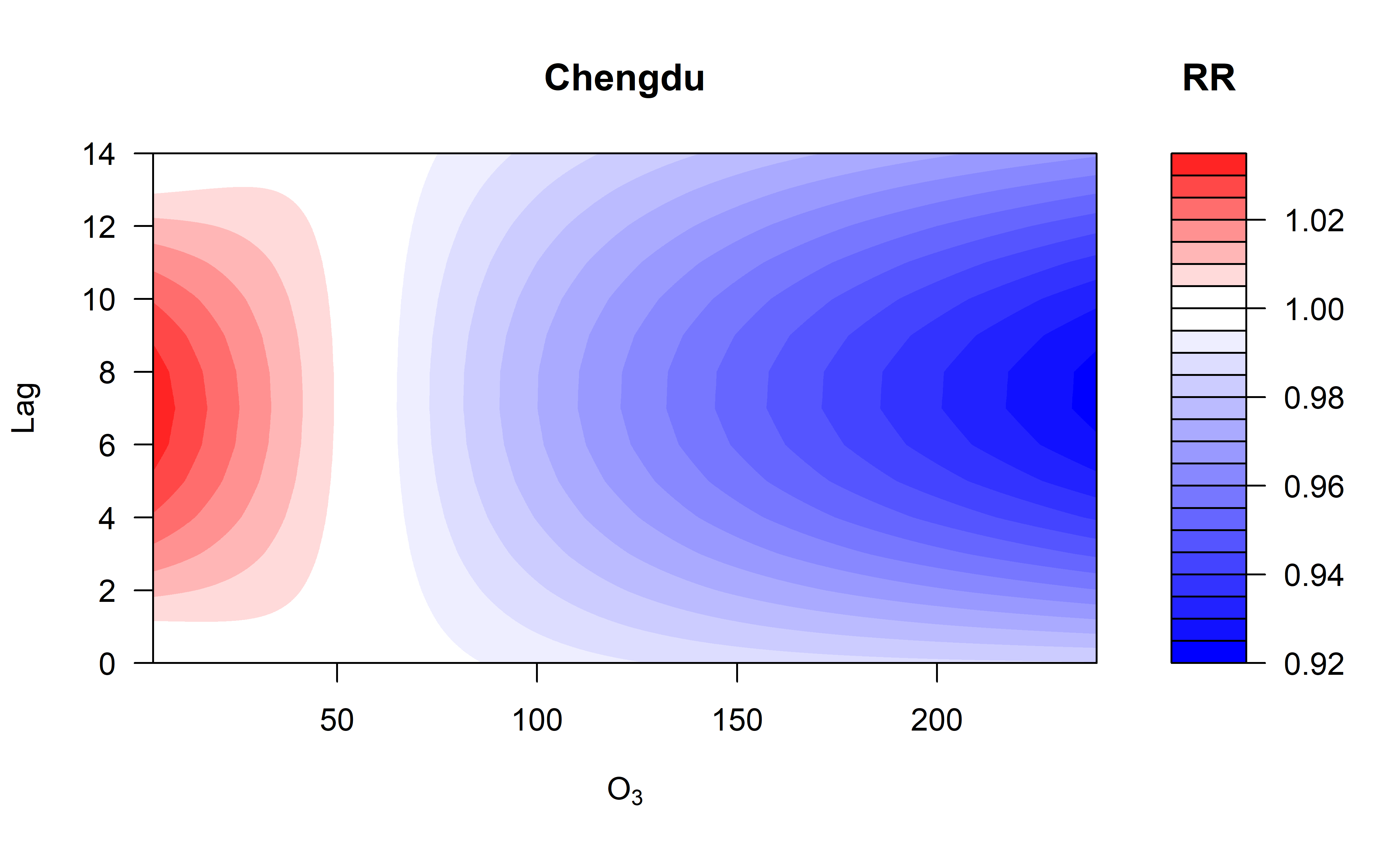

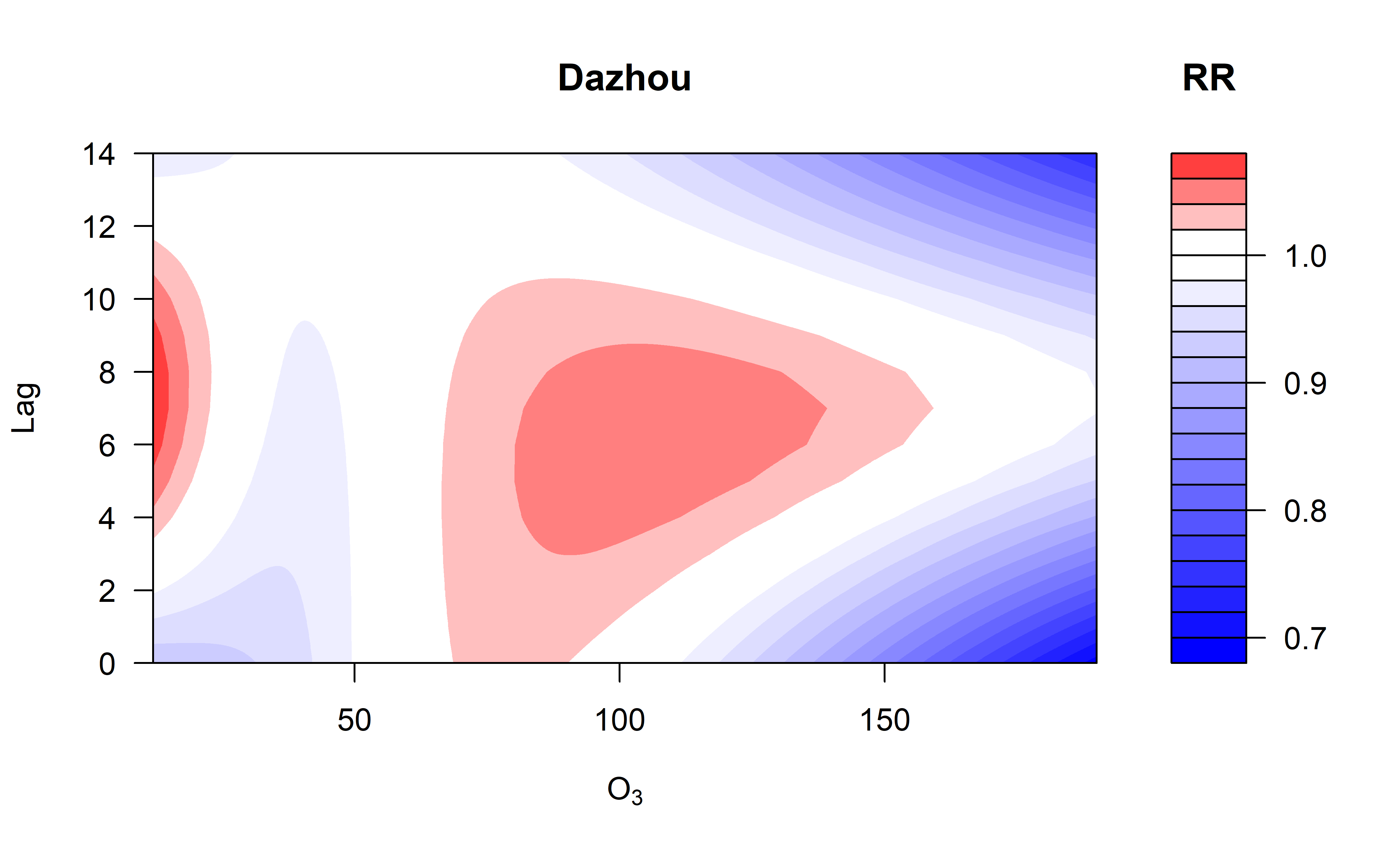

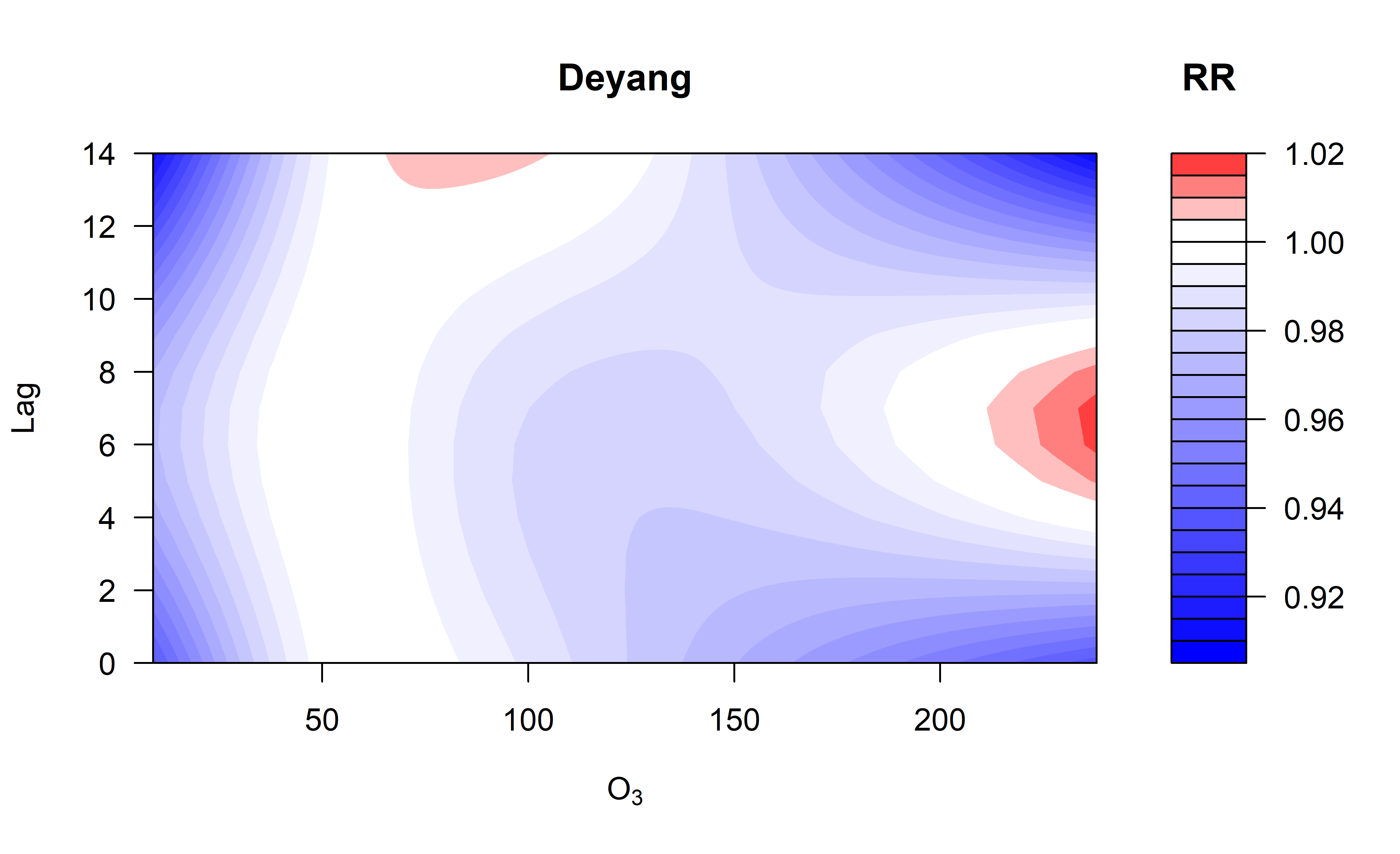

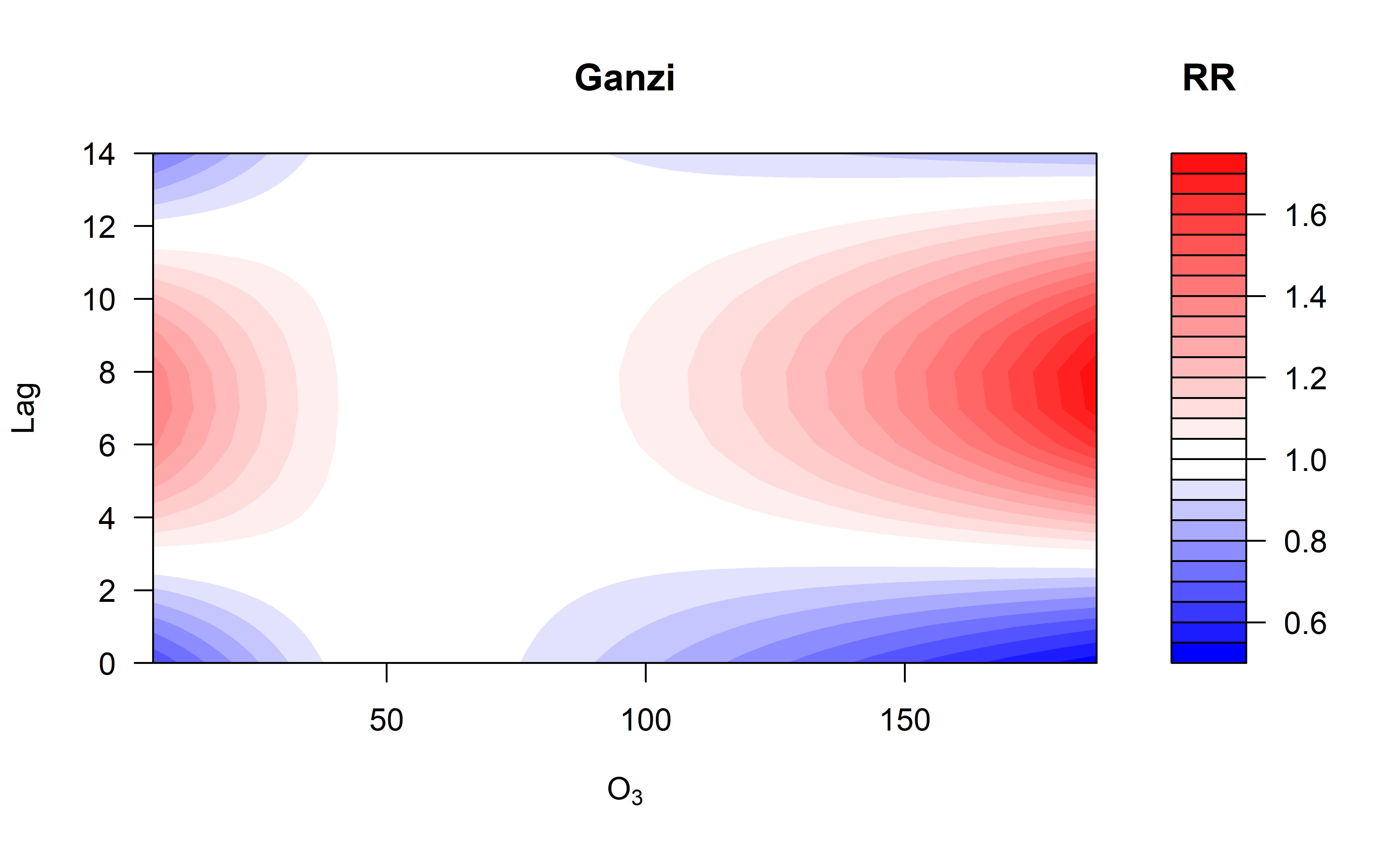

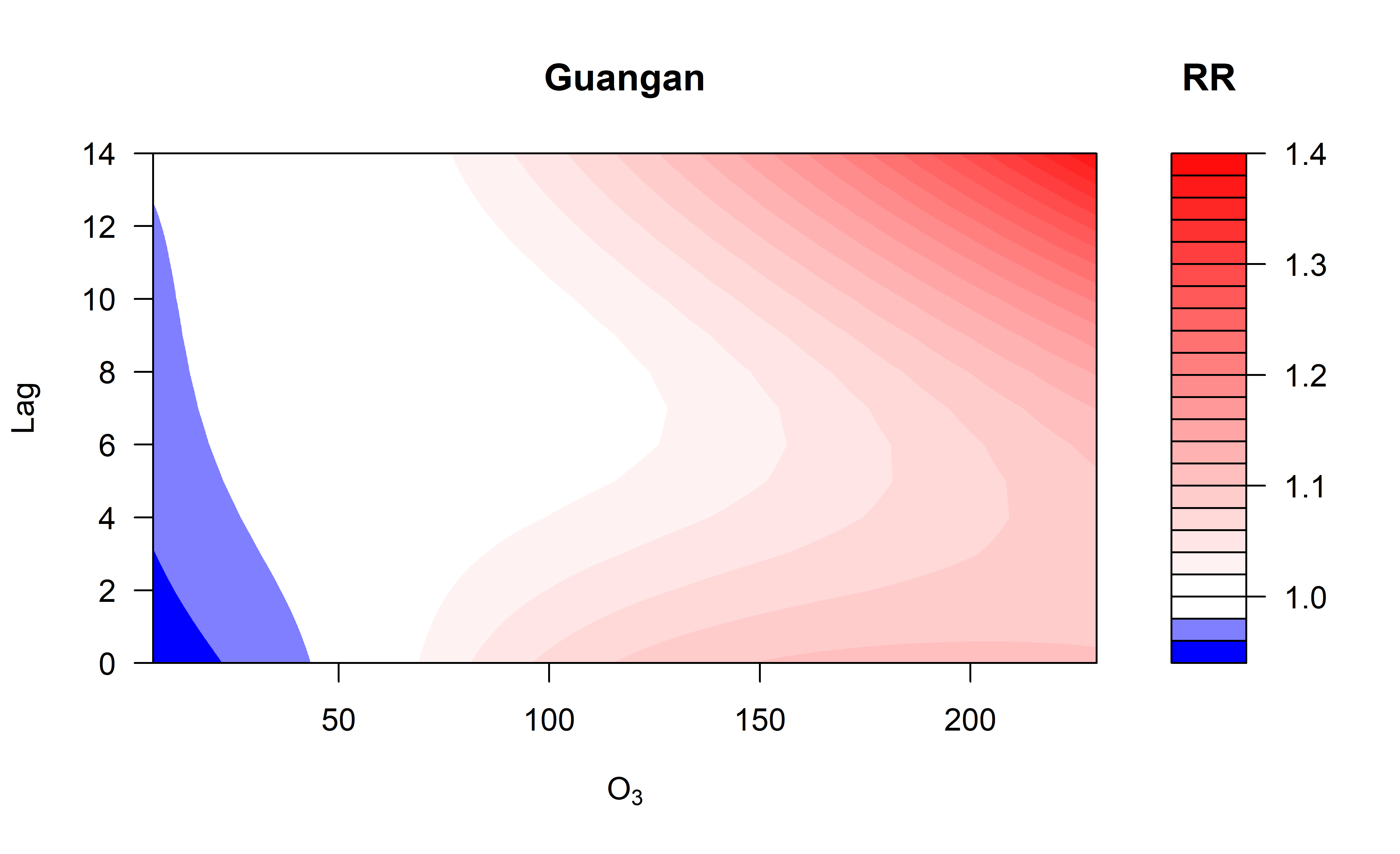

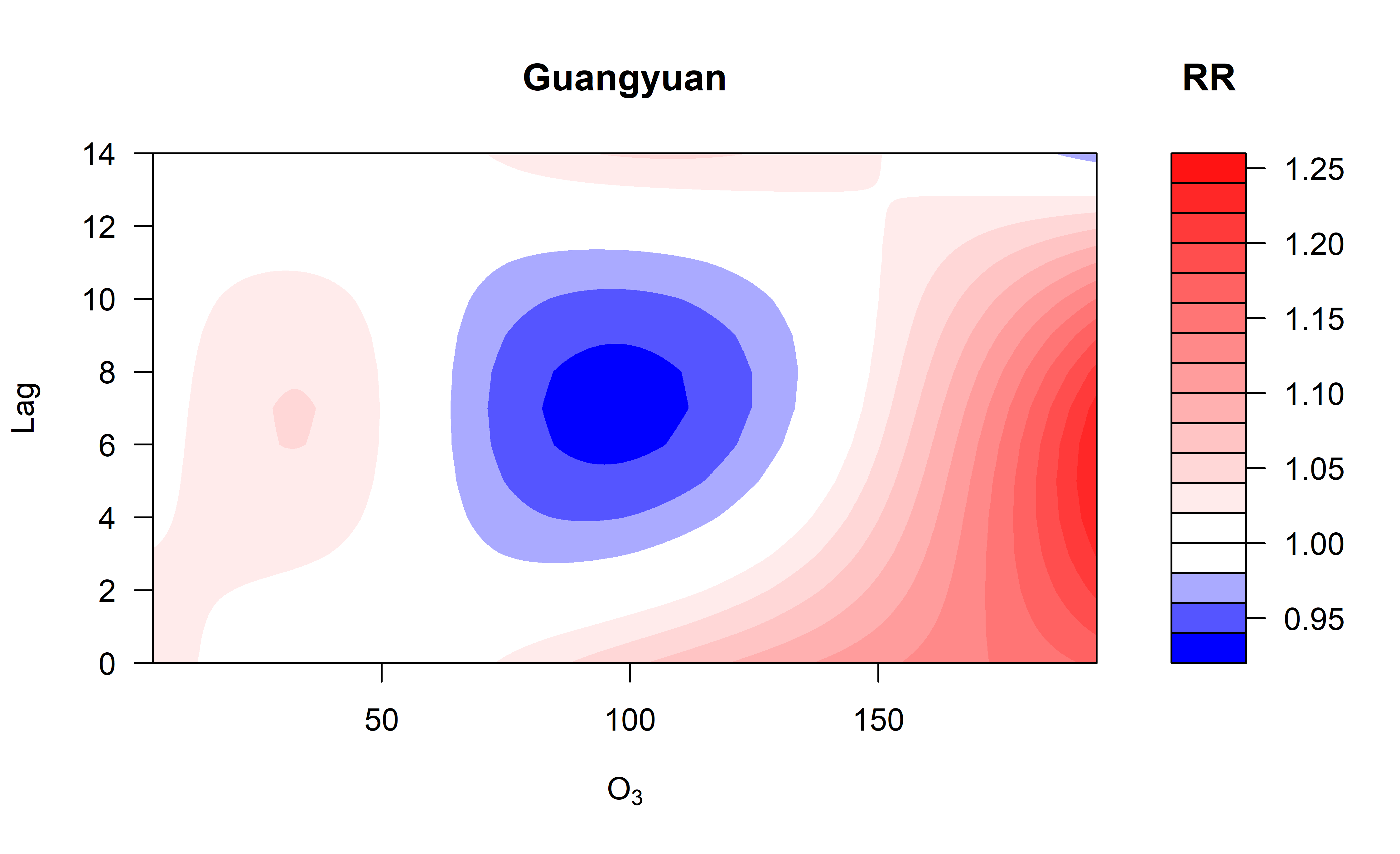

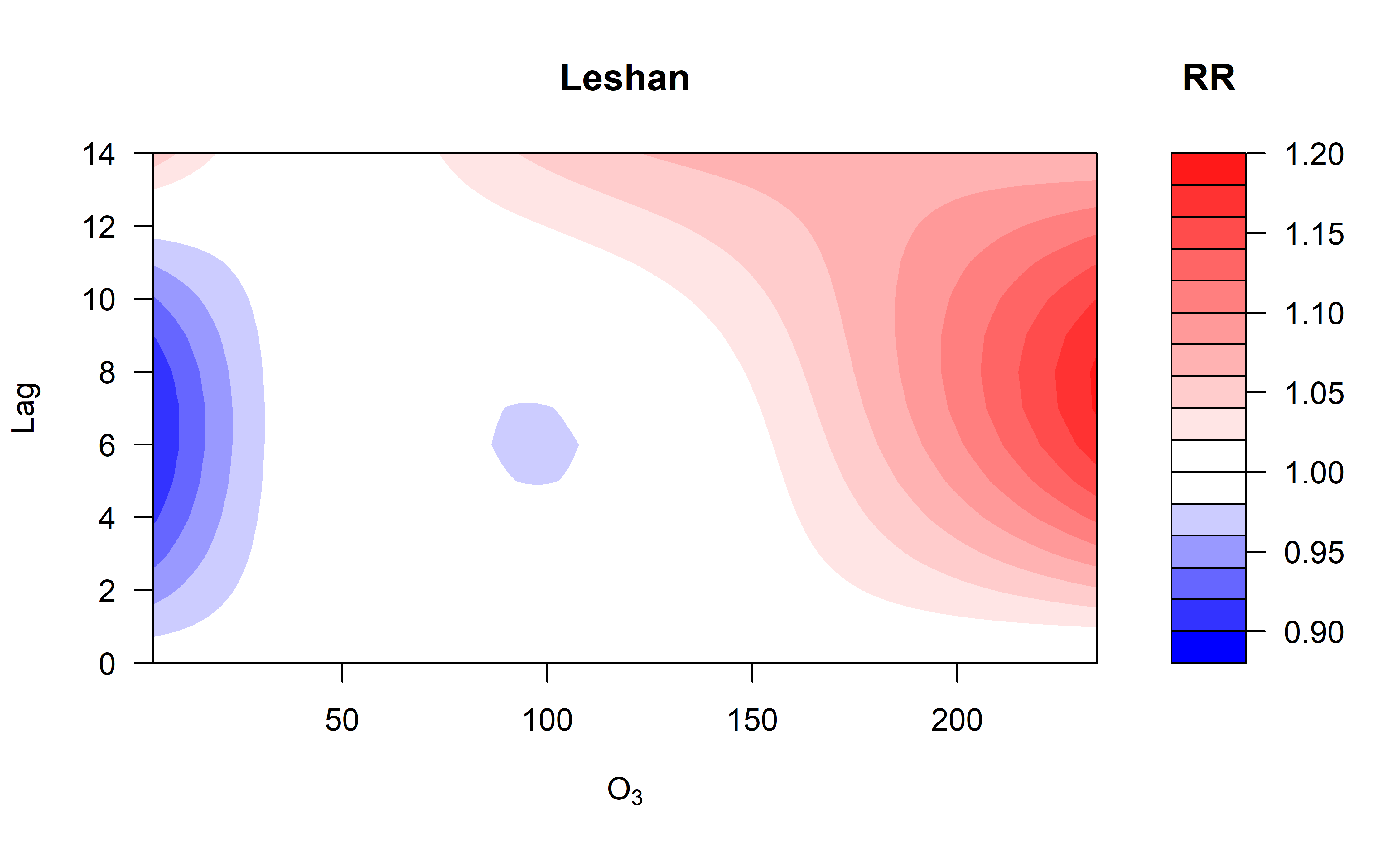

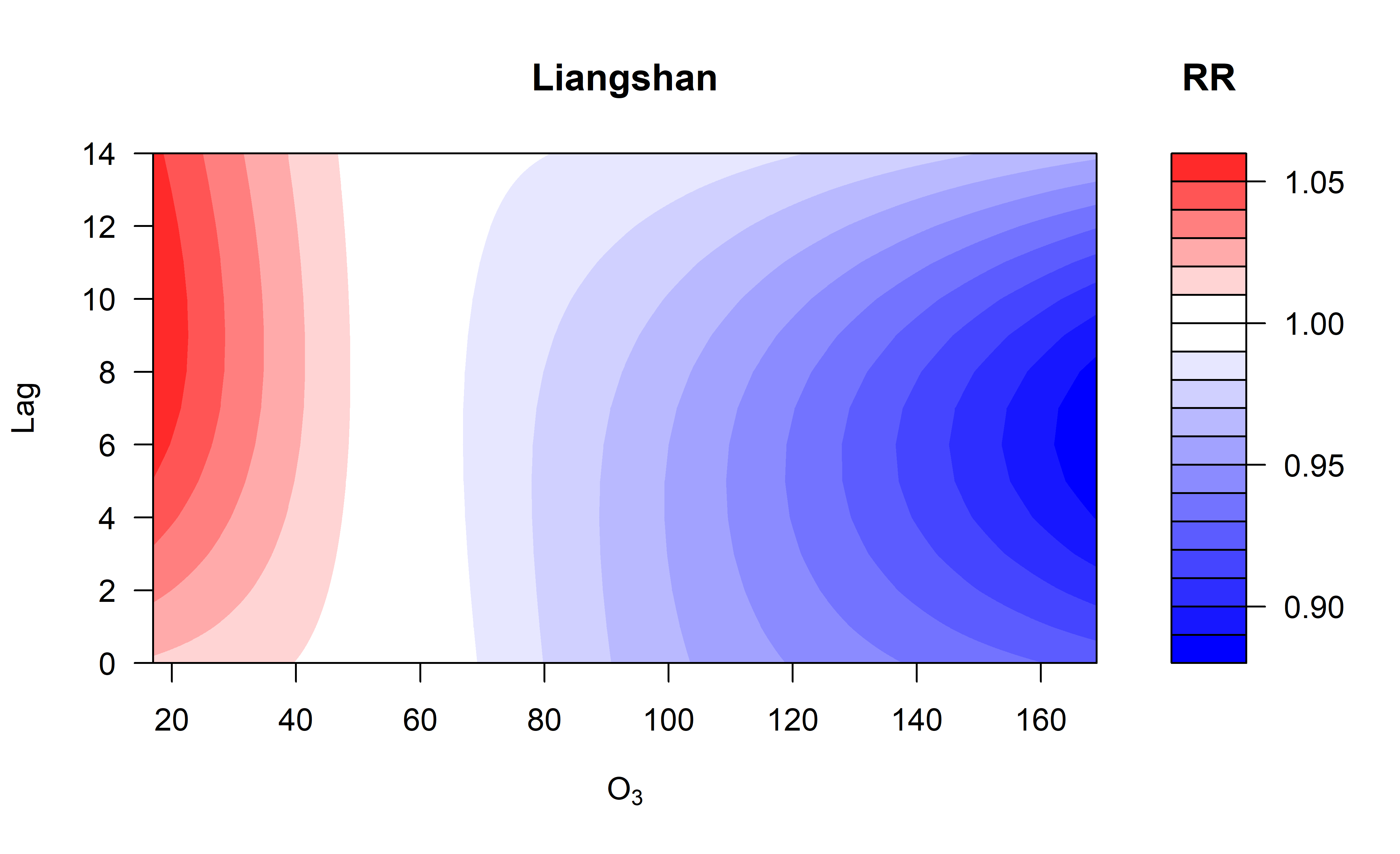

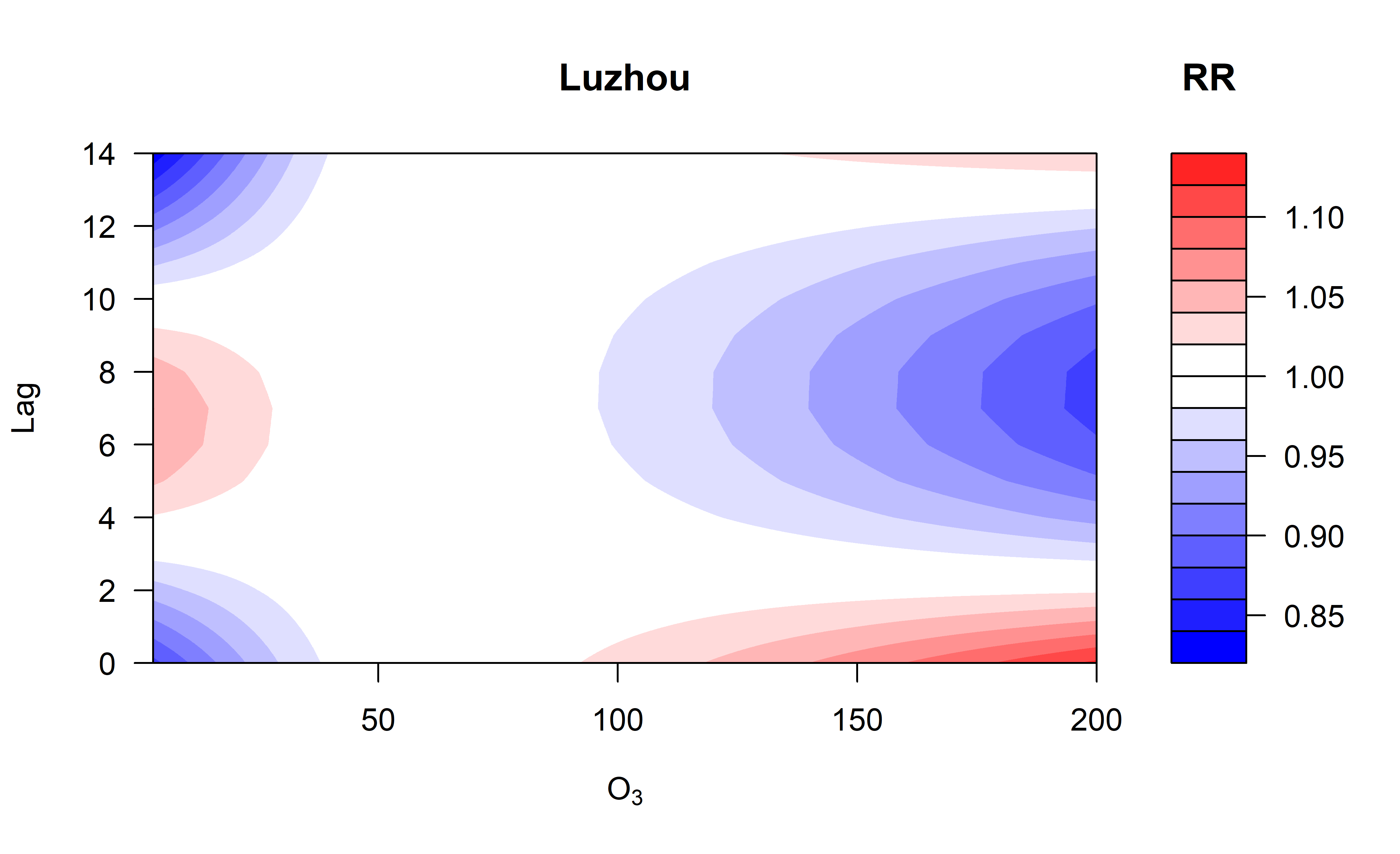

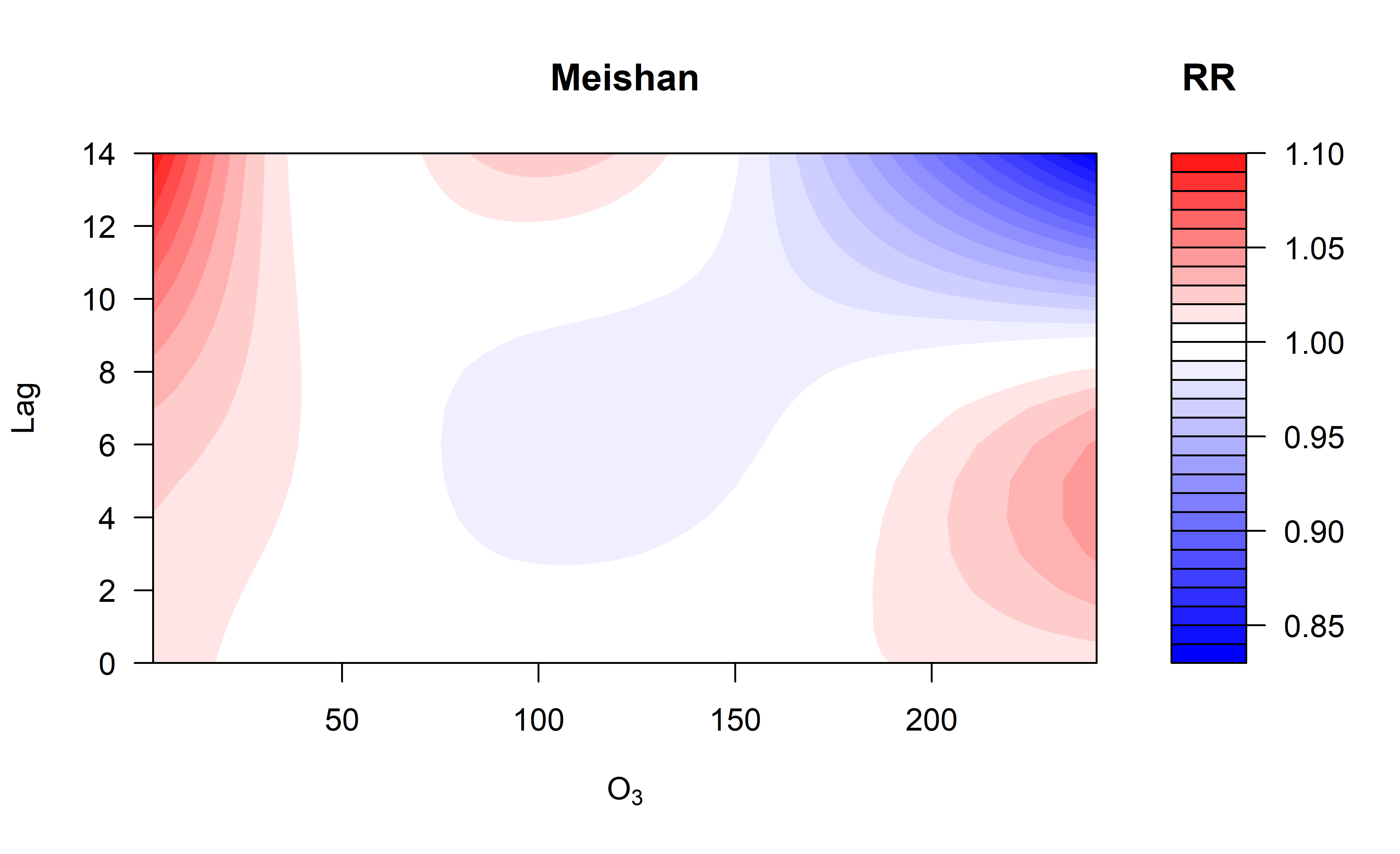

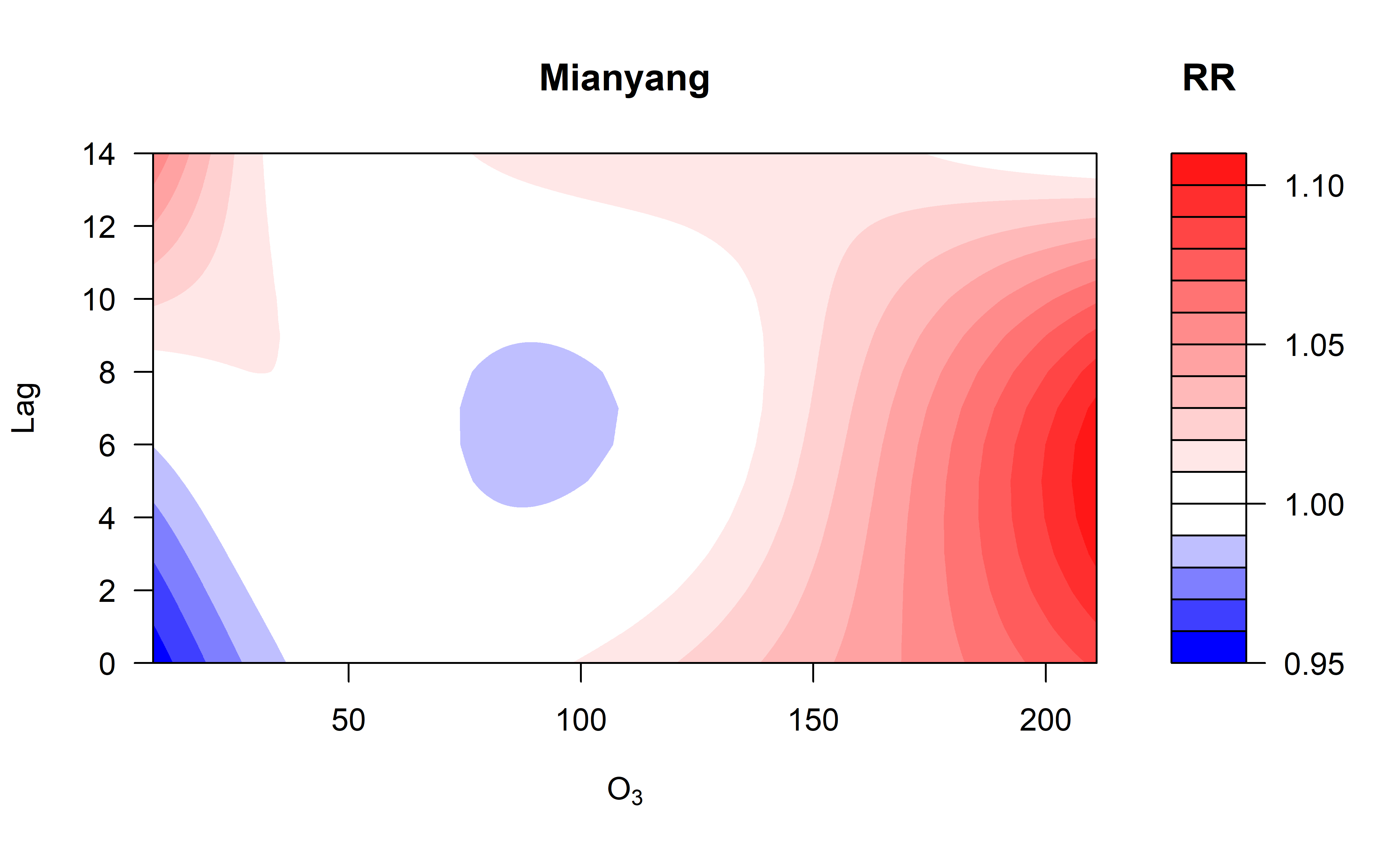

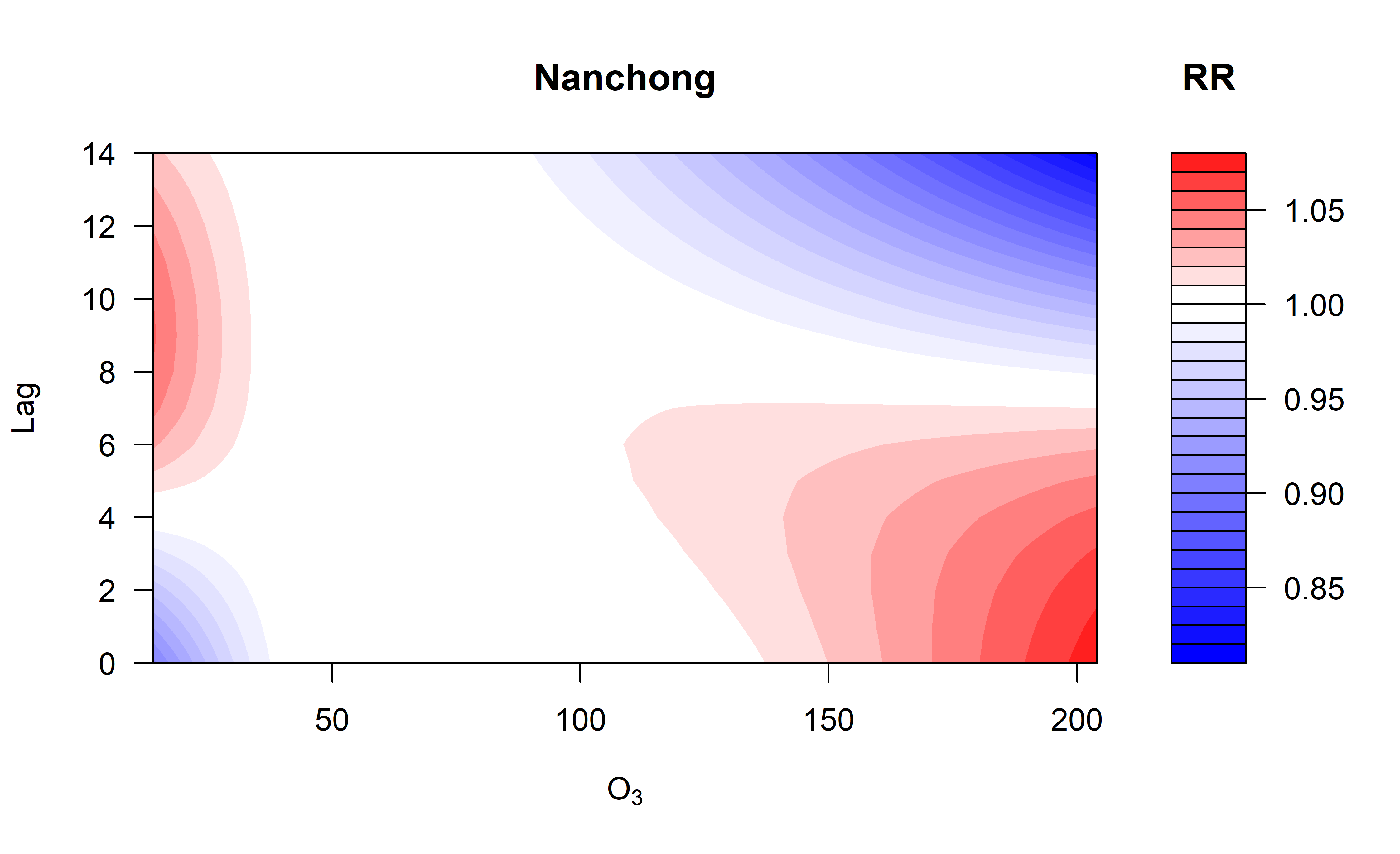

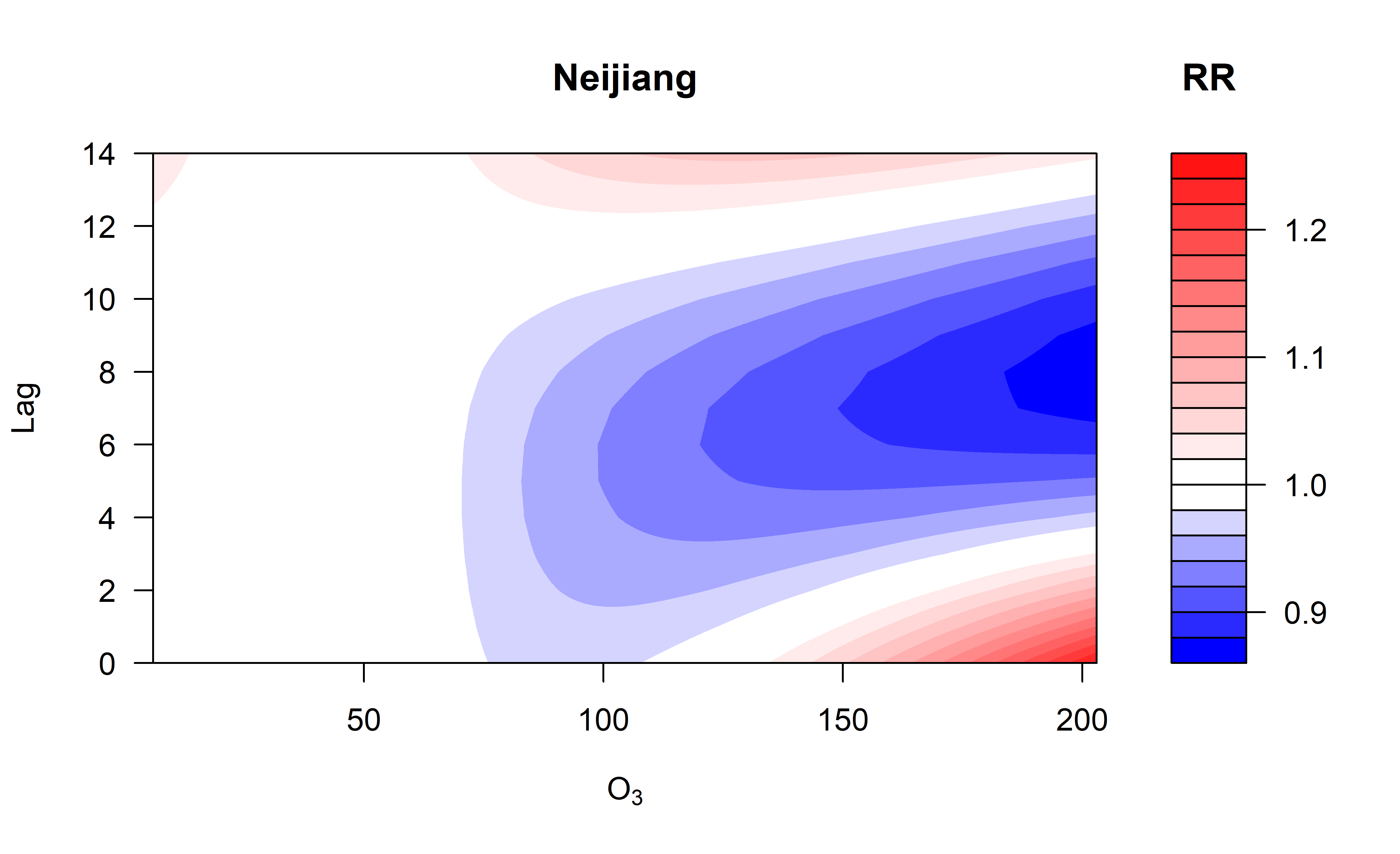


Fig. S6. Contour plots of the city-specific relationship between the risk of HFMD and O_3_ at different time lags.

**Text S1. Sensitivity Analysis**

We established uniform model structures and parameters for each city by considering prior knowledge and conducting a series of sensitivity analyses based on the multi-pollutant model. These sensitivity analyses were based on empirical data from the 21 cities in Sichuan Province. Quasi Akaike Information Criterion (QAIC) and the average of effect estimates were calculated to evaluate the model choices.

1. The choice of the dfs for controlling long-term trend and seasonality.

To control long-term trends and seasonality, a sensitivity analysis was performed by varying the dfs of time splines from 1 to 15 per year. The results are presented in Figure S7. When the dfs of natural cubic splines exceeded 8, both the average estimates of air pollutants effects and model fits remained stable. Therefore, we set the dfs per year to 8 to control long-term trends and seasonality.

Fig. S7. The overall model fits and average estimates of air pollution effects from the 21 cities for different dfs of time splines.

2. The choice of parameters for the lag-response dimension.

Splines with 3-5 dfs were commonly used to capture the lag distribution when modeling the relationship between environmental factors and infectious diseases. Therefore, we performed a series of sensitivity analyses to determine the dfs of natural cubic splines for the lagged effects of air pollutants by varying the dfs from 3 to 5. The results are presented in Table S1 and Figure S8. Natural cubic splines for the effects of air pollutants with 3 dfs show the best goodness of model fit, and different dfs yielded robust shapes of the air pollutants-HFMD curve.

In addition, we also changed the location of knots for the lag-response dimension. The results showed that the patterns of the overall pooled curve were little affected (Fig. S9). In consequence, we used natural cubic splines of 3 dfs with the knots placed at equally spaced values for lag effects which seem sufficient to express the complexity of the lag distribution in the final model.

Fig. S8. Overall relationship of HFMD counts with air pollutants at different splines with 3-5 dfs for the lag-response dimension.

Table S1. The model fit (QAICs values) for different setting of dfs in the lag-response dimension of each air pollutant.

| dfs | NO_2_ | PM_10_ | O_3_ | CO | SO_2_ |
| --- | --- | --- | --- | --- | --- |
| 3 | 102562.3 | 102562.3 | 102562.3 | 102562.3 | 102562.3 |
| 4 | 102584.1 | 102618.7 | 102645.8 | 102616.1 | 102624.1 |
| 5 | 102624.0 | 102684.6 | 102721.0 | 102661.7 | 102675.0 |

Fig. S9. Overall relationship of HFMD counts with air pollutants at different locations of knots for the lag-response dimension.

3. The choice of parameters for the exposure-response dimension.

We performed a systematic sensitivity analysis to determine the dfs of natural cubic splines for the effects of air pollution by varying the dfs from 3 to 8. The more flexible shapes of the air pollutants-HFMD curves were observed with the increase of dfs (Fig. S10). According to the overall model fit index, we found that QAIC values of the DLNMs also increase with increased dfs for each air pollutant (Table S2). Therefore, we used natural cubic splines for the exposure-response dimension with 3 dfs to achieve the best performance of the model fit.

In addition, we also changed the places of knots for the exposure-response dimension (equally spaced values, 30th and 70th percentiles, 20th and 80th percentiles 15th and 85th percentiles 10th and 90th percentiles). The shapes of the overall pooled air pollution-HFMD curves were very robust (Fig. S11). Therefore, we placed the knots for the splines of the exposure-response dimension at equally spaced values in the final model.

Fig. S10. Overall relationship of HFMD counts with air pollutants at different splines with 3-8 dfs for the exposure-response dimension.

Table S2. The model fit (QAICs values) for different setting of dfs in the exposure-response dimension of each air pollutant.

| dfs | NO_2_ | PM_10_ | O_3_ | CO | SO_2_ |
| --- | --- | --- | --- | --- | --- |
| 3 | 102562.3 | 102562.3 | 102562.3 | 102562.3 | 102562.3 |
| 4 | 102603.3 | 102573.7 | 102600.0 | 102631.9 | 102585.2 |
| 5 | 102639.9 | 102657.3 | 102628.6 | 102698.9 | 102577.4 |
| 6 | 102638.2 | 102658.7 | 102662.2 | 102744 | 102611.6 |
| 7 | 102670.2 | 102644.1 | 102660.4 | 102750.5 | 102635.1 |
| 8 | 102733.8 | 102649.0 | 102732.2 | 102776.2 | 102620.6 |

Fig. S11. Overall relationship of HFMD counts with air pollutants at different locations of knots for the exposure-response dimension.

4. The choice of terms for controlling the confounding of temperature.

We compared 9 different models for controlling the confounding of temperature by varying the association pattern (i.e., linear or nonlinear) and lag structure (i.e., how many lags should be included and how to constrain the lag structure). The detailed settings of the confounder models are shown in Table S3. Model C0 is the null model with no confounder. Models C1-C4 are based on the assumption of a linear relationship of the temperature-HFMD association pattern. Model C1 includes only one lag, which is set to the median of the incubation period. Models C2 to C4 include multiple lags but use different methods to summarize the multiple lags through simple and exponential moving weighted averages and distributed lag models. A natural cubic spline with 3 dfs is applied to constrain the distributed lag model. Similarly, models C5-C8 are based on the assumption of a nonlinear relationship of the association pattern. A natural cubic spline with 3 dfs is applied to describe the nonlinear relationship. The average estimates of air pollution effects and model fits are presented in Figure S12. The confounder model (C6), based on the assumption of a nonlinear relationship with multiple lags and using a simple moving weighted average to constrain the lag structure, has the best model fit.

Table S3. Different confounder model settings of temperature.

| Notation | Association pattern | |  | Lag structure | | Symbol |
| --- | --- | --- | --- | --- | --- | --- |
|  | Exposure | df |  | interval | constraint form |  |
| C0 | None | - |  | - | - |  |
| C1 | Linear | - |  | lag4 | - | Temp*_t_*,*_l_* |
| C2 | Linear | - |  | lags 4-10 | Simple moving average | SMA(Temp*_t_*,*_l_*) |
| C3 | Linear | - |  | lags 4-10 | Exponential moving average | EMA(Temp*_t_*,*_l_*) |
| C4 | Linear | - |  | lags 4-10 | Distributed lag model | cb(lin(Temp), ns(lag)) |
| C5 | Nonlinear | 3 |  | lag 4 | - | ns(Temp*_t_*,*_l_*) |
| C6 | Nonlinear | 3 |  | lags 4-10 | Simple moving average | ns(SMA(Temp*_t_*,*_l_*)) |
| C7 | Nonlinear | 3 |  | lags 4-10 | Exponential moving average | ns(EMA(Temp*_t_*,*_l_*)) |
| C8 | Nonlinear | 3 |  | lags 4-10 | Distributed lag model | cb(ns(Temp), ns(lag)) |

Fig. S12. Overall model fits and average estimates of air pollution effects in the 21 cities for different confounder model settings of the mean temperature.
